# Covalent organic framework membrane with hourglass-shaped nanochannels for ultrafast desalination

**DOI:** 10.1038/s41467-025-63650-5

**Published:** 2025-08-30

**Authors:** Xiaocui Wei, Yanan Liu, Fu Zhao, Tingyuan Wang, Zongmei Li, Chunyang Fan, Yuhan Yang, Yuhan Wang, Zhongyi Jiang

**Affiliations:** 1 School of Chemistry and Chemical Engineering, Collaborative Innovation Center of Ecological Civilization, Hainan University, Haikou, China; 2 Key Laboratory for Green Chemical Technology of Ministry of Education, School of Chemical Engineering and Technology, Tianjin University, Tianjin, China

**Keywords:** Organic-inorganic nanostructures, Two-dimensional materials, Pollution remediation

## Abstract

Covalent organic framework (COF) holds great potential as next-generation high-performance desalination membrane material owing to their uniform nanochannels (homo-nanochannels) and abundant functional groups, and the hierarchical structures of nanochannels should be rationally designed to break the trade-off between water permeability and ion rejection. Here, a kind of COF membrane with hourglass-shaped nanochannels is fabricated by installing amino-cyclodextrin nanoparticles (CDN) onto the mouth of COF membrane via sequential assembly. The resulting hetero-nanochannels consist of a hydrophilic conical entrance (~1.6 nm) and a hydrophobic spout (~0.5 nm), created by the CDN specific cavity, onto the homo-nanochannels of COF with intrinsic nanopores (~1.4 nm). The hydrophilic conical entrance facilitates the entry of water molecules, whereas the hydrophobic spout and the homo-nanochannels collectively enable fast water transport. Meanwhile, the amino groups on CDN endow the hetero-nanochannels with pH-responsive ability to dynamically regulate their effective size and charge. Accordingly, the optimum COF-CDN membrane exhibits high desalination performance, with a water flux of 98 L m^–2^ h^–1^, and rejection of 94% for Na_2_SO_4_ and 92% for NaCl. The COF-CDN membrane also exhibits superior operational stability (7 days) and pH cycle stability, validating the utilization of COF membrane in efficient desalination.

## Abstract (web summary)

Covalent organic frameworks (COF) are an important desalination membrane material but rational design of the hierarchical structures of nanochannels remains challenging. Here the authors fabricate a COF membrane with hourglass-shaped nanochannels by installing aminocyclodextrin nanoparticles onto the mouth of COF membrane via sequential assembly.

## Introduction

Seawater desalination, as an open-source technology for water resource utilization, can alleviate the growing global water shortage^1^. Membrane technology, owing to its advantages of high energy efficiency and easy scale-up, possesses its advantages in desalination^2–4^. Covalent organic framework (COF), as a class of nanoporous crystalline polymers^5–8^, are featured by high surface area and porosity, tunable pore structures, readily amenable surface properties as well as chemical stability, which hold great potential as next-generation desalination membranes^9,10^. The uniform nanochannels (homo-nanochannels) as well as abundant functional organic groups can be controlled through regulating the type and size of COF monomers^11,12^. However, the homo-nanochannels in COF membranes often lead to the trade-off between water permeability and ion rejection^13–15^. Moreover, the COF homo-nanochannels with diameter less than 1.0 nm are difficultly available at present^16,17^. Therefore, the hierarchical structures of nanochannels in COF membranes should be rationally designed for efficient desalination.

The hourglass-shaped nanochannels have emerged as a promising structure design, featuring a narrow spout flanked by wider entrances at both ends to balance water permeability and ion rejection^18,19^. In order to increase water permeability, the nanochannels with asymmetric wettability including a hydrophilic entrance and a hydrophobic channel body are explored to enable the capture of water as well as fast and low friction transport of water^20–22^. Because the hydrophobic channel wall can ensure fast water flow through weak wall-fluid interactions and the hydrophilic entrance can avoid the high energy barrier for water entry^23,24^. Moreover, specific charged groups are introduced into nanochannels to enhance ion rejection through the Donnan effect, enabling precise separation of ions and improving membrane performance^25^. Hence, constructing the hourglass-shaped nanochannels with asymmetric wettability and specific charged groups, may find promising applications in high-performance desalination membranes.

**Fig. 1:**
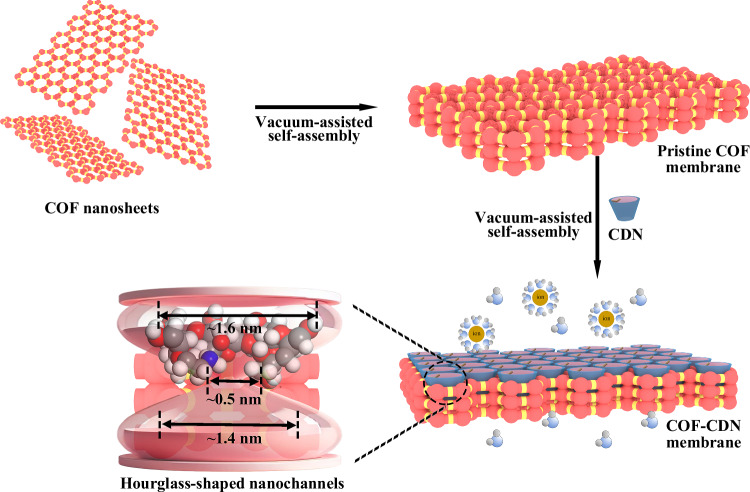
Schematic illustration of fabricating COF membranes for desalination. Illustration of constructing a COF membrane with hourglass-shaped nanochannels (COF-CDN) via sequential assembly of installing amino-cyclodextrin nanoparticles (CDN) onto the mouth of COF membrane. The resulting hourglass-shaped nanochannels consist of a hydrophilic conical entrance (~1.6 nm), a hydrophobic spout (~0.5 nm), and the homo-nanochannels of COF with intrinsic nanopores (~1.4 nm). This unique architecture enhances both water permeability and ion rejection, offering improved performance for desalination applications.

Cyclodextrin (CD) is a popular supermolecule characterized by specific cavity with a hydrophilic entrance and a hydrophobic spout, creating precisely structured pathways for mass transport and enabling the construction of low-energy-barrier nanochannels in membranes^26–29^. Hence, combining the specific cavity of CD with highly ordered homo-nanochannels of COF membranes, the hourglass-shaped nanochannels are expected to be constructed for fast and efficient desalination. Herein, we fabricate a kind of COF membrane with hourglass-shaped nanochannels by installing amino-cyclodextrin nanoparticles (CDN) onto the mouth of COF membrane via sequential assembly and evaluate its desalination performance (Fig. 1). The resulting hetero-nanochannels consist of a hydrophilic conical entrance and a hydrophobic spout from CDN as well as the homo-nanochannels of COF with intrinsic nanopores. The hydrophilic conical entrance facilitates the entry of water molecules, whereas the hydrophobic spout and the homo-nanochannels collectively enable fast water transport. Meanwhile, the amino groups on CDN endow the hetero-nanochannels with pH-responsive ability to dynamically regulate their effective size and charge. The COF-CDN membranes exhibit high water flux and salt rejection, much higher than those of the pristine COF membrane, along with superior operational stability (7 days) and enhanced pH cycle stability. Our study may inspire the design of COF membranes with hetero-nanochannels, breaking the trade-off between membrane permeability and selectivity in various chemical separations.

## Results

### Fabrication and characterization of COF-CDN membranes

**Fig. 2:**
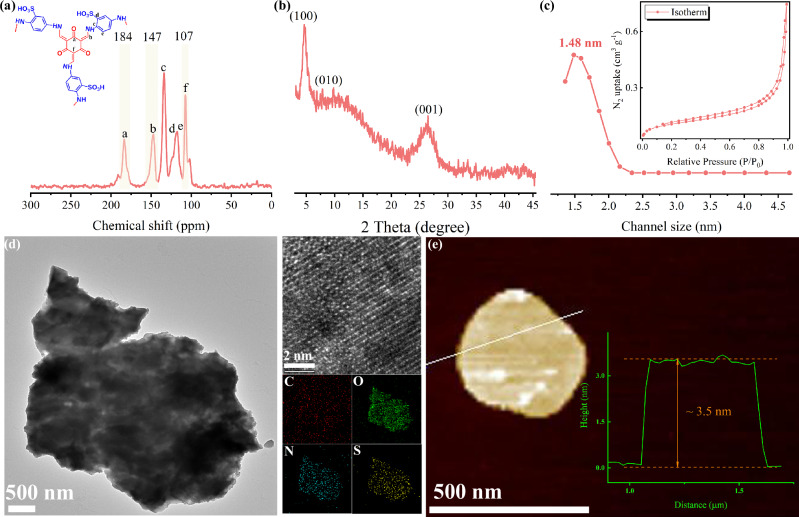
Characterization of TpPa-SO_3_H COF nanosheets. **a** Solid-state ¹³C NMR spectra; **b** XRD pattern; **c** N_2_ adsorption-desorption isotherm; **d** TEM image, HRTEM image in the upper insets and elemental mapping in the lower insets; **e** AFM image, the height data shown in the inset.

Schiff-base type COF have been extensively explored owing to their high crystallinity and chemical stability^30,31^. Here, TpPa-SO_3_H COF nanosheets were synthesized using 1,3,5-triformylphloroglucinol (Tp) and 2,5-diaminobenzenesulfonic acid (Pa-SO_3_H) via a two-phase method to obtain high-quality, well-defined nanosheets (Supplementary Fig. 1). The FT-IR spectra (Supplementary Fig. 2) of the TpPa-SO_3_H COF nanosheets revealed the disappearance of peaks corresponding to the amino groups at 3300–3450 cm^–1^ and the aldehyde groups at 2890 and 1650 cm^–1^. A new peak appeared around 1600 cm⁻¹, corresponding to the imine (–C = N–) bonds formed during the Schiff-base reaction. These spectral changes confirmed the successful construction of the TpPa-SO_3_H COF nanosheets^32^. The solid-state ^13^C NMR spectra further corroborated this observation (Fig. 2a), with characteristic peaks at 184 ppm and 147 ppm corresponding to the keto (–C = O) and imine (–C = N–) carbons, thereby verifying the formation of the β-ketoenamine structure and Schiff-base linkages^33,34^. The characteristic peaks at 115–125 ppm, corresponding to the aromatic carbons, verify that the aromatic structure was preserved. The absence of signals corresponding to aldehyde and amino groups confirmed the completion of the reaction. Combined with the FT-IR spectra, these results confirmed the successful synthesis of TpPa-SO_3_H COF nanosheets. The X-ray diffraction (XRD) technique was used to evaluate the crystallinity of TpPa-SO3H COF nanosheets (Fig.2b). The XRD pattern exhibited clear and sharp peaks at specific angles, confirming a well-ordered crystalline structure. A strong peak around 4.6° matched the (100) plane, typical for the two-dimensional layered structure of COF. Additional peaks at higher angles, like those near 25.0–30.0°, corresponded to the (001) reflections, confirming the layers stacked in an orderly manner. These patterns matched the expected XRD data for TpPa-SO_3_H COF nanosheets, validating the crystalline framework exhibited ideal crystallinity, consistent with the solvothermally synthesized TpPa-SO_3_H COF powder and previous literature (Supplementary Fig. 3)^35^. The nitrogen adsorption–desorption analysis (Fig. 2c) revealed a pore size of 1.4 nm for the TpPa-SO_3_H COF nanosheets, consistent with the predicted pore size and indicating an intrinsic long-range ordered structure.

Transmission electron microscopy (TEM) image (Fig. 2d) revealed that the TpPa-SO_3_H COF nanosheets had a transverse dimension of 1–3 µm, reflecting their large, planar structure. High-resolution TEM (HRTEM) image displayed well-defined lattice fringes, confirming the excellent crystallinity of the nanosheets, which was consistent with the XRD results. Elemental mapping confirmed that the distribution of carbon (C), oxygen (O), nitrogen (N), and sulfur (S) was uniform across the nanosheets. Additionally, atomic force microscopy (AFM) image indicated that the thickness of the nanosheets was approximately 4 nm, confirming their nanoscale dimensions and uniformity (Fig. 2e and Supplementary Fig. 4). These observations collectively validated the structural integrity and consistency of the TpPa-SO_3_H COF nanosheets. In addition, thermogravimetric (TGA) curves revealed that the nanosheets decomposed at approximately 250 °C, which demonstrated that they maintained good thermal stability up to this temperature (Supplementary Fig. 5). This indicated that the nanosheets exhibited thermal structure, retaining their structural integrity up to 250 °C.

**Fig. 3:**
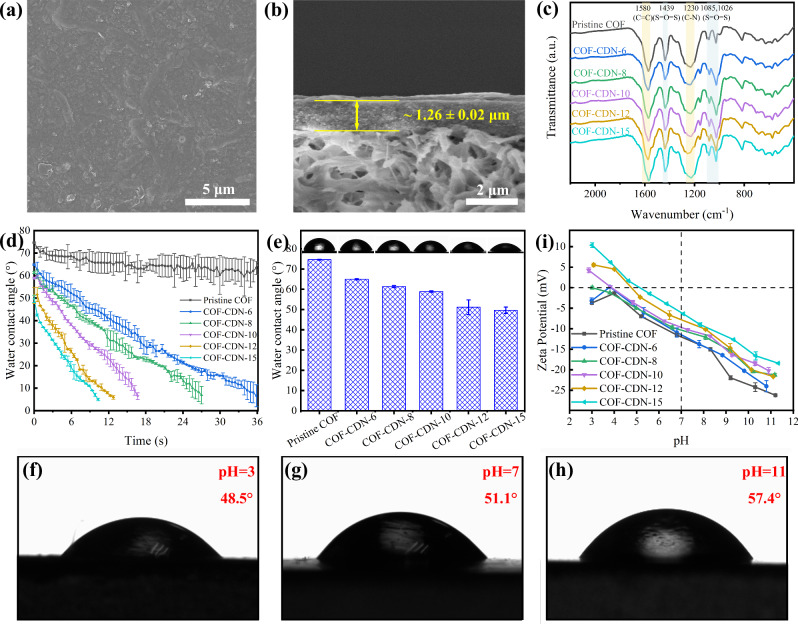
Characterization of pristine COF and COF-CDN membranes. **a** Surface SEM image and **b** cross-sectional SEM image of COF-CDN-12 membrane; **c** FT-IR spectra; **d** dynamic contact angle; **e** water contact angle in the air; the water contact angle of COF-CDN-12 membrane at different pH values, including **f** pH = 3; **g** pH = 7; **h** pH = 11; **i** zeta potential.

The COF membranes with hourglass-shaped nanochannels were fabricated by assembling COF nanosheets onto a Nylon substrate membrane (which features a porous structure and high stability, Supplementary Fig. 6), followed by the installing of amino-cyclodextrin nanoparticles (CDN) onto the mouth of COF membrane (Fig. 1). The alignment of CDN relied on hydrogen bonding and electrostatic interactions between the amino (–NH_2_) or hydroxyl (–OH) groups in CDN and the sulfonic acid (–SO_3_H) groups in the COF^36,37^. These interactions not only stabilized CDN at the COF mouth but also promoted an ordered arrangement by providing directional forces and enhancing structural organization^38,39^. The morphology of COF membranes with hourglass-shaped nanochannels (COF-CDN) was investigated using surface and cross-sectional SEM analysis (Supplementary Fig. 7–10). The pristine COF membrane, fabricated with TpPa-SO_3_H COF nanosheets without installing CDN at the nanochannel mouth, exhibited a dense surface with no visible cracks (Supplementary Fig. 7a). As the CDN content increased, the surface SEM images remained a dense structure, indicating that the increase of CDN did not generate any cracks or defects on the surface of COF-CDN membranes (Fig. 3a and Supplementary Fig. 7). Moreover, the thickness of COF-CDN membranes was observed to increase compared to the pristine COF membrane (Supplementary Figs. 8, 9). For example, the increase in membrane thickness from approximately 0.19 ± 0.01 µm for the pristine COF membrane (Supplementary Fig. 8) to about 1.26 ± 0.02 µm with the COF-CDN-12 membrane (Fig. 3b), which was attributed to the introduction of CDN. This phenomenon was clearly observed in the cross-sectional SEM images (Supplementary Fig. 8), where the separation layer thickness of COF-CDN membranes was significantly greater than that of the pristine COF membrane. Meanwhile, we fabricated a COF-CD-12 membrane by installing cyclodextrin (CD) without amino group onto the mouth of COF membrane. As shown in Supplementary Fig. 10, the surface SEM image of the COF-CD-12 membrane reveals a defect-free morphology (Supplementary Fig. 10a), and the cross-sectional SEM image showed both a defect-free morphology and a uniform thickness of approximately 0.97 ± 0.04 µm (Supplementary Fig. 10b). The amino groups enhanced the interaction between the CDN and the COF nanosheets, whereas in the absence of amino groups, as in COF-CD-12 membrane, this enhanced interaction was lacking, resulting in a thinner membrane, although it still maintained a uniform structure. Additionally, AFM analysis of the increased separation layer revealed an increase in surface roughness (Supplementary Fig. 11). The AFM images revealed that the surface roughness of COF-CDN membranes was significantly higher than that of the pristine COF membrane. The increase in both the arithmetic mean roughness (Ra) and the root mean square roughness (Rq), which indicated higher surface roughness, was attributed to the increase of CDN within the membrane (Supplementary Fig. 12). This resulted in the formation of larger and more irregular surface features, due to the heterogeneous distribution of CDN, which caused uneven surface textures during the membrane formation process^40^. The FT-IR spectra of COF-CDN membranes (Fig. 3c and Supplementary Fig. 13) demonstrated that the introduction of CDN did not interfere with the imine (–C = N–) and sulfonic acid (–SO_3_H) groups in COF nanosheets. This indicated that the chemical environment of these functional groups remained intact. To further investigate the effects of different pH environmental conditions, the membrane was soaked in 1 M NaOH and HCl for 24 h. After the treatment, the FT-IR spectra (Supplementary Fig. 13) indicated that the imine and sulfonic acid groups remained intact, thereby demonstrating the excellent chemical stability of membrane under different pH environmental conditions.

### Wettability of the COF-CDN membranes

The wettability of the membrane surface was evaluated by measuring the water contact angle (Fig. 3d-h). Figure 3d showed the dynamic contact angles of pristine COF and COF-CDN membranes as a function of time. The pristine COF membrane exhibited a slow variation in dynamic contact angle, indicating limited wettability. This behavior can be attributed to its relatively smooth surface, as evidenced by surface SEM images (Supplementary Fig. 7) and the low roughness observed in Supplementary Fig. 12, which provide fewer interaction sites for water molecules and result in slower spreading across the membrane surface. After the increase of CDN, the surface roughness increased (Supplementary Fig. 12), which, combined with the inherent hydrophilicity of the membrane, led to greater surface energy and enhanced water molecules spreading, thereby reducing the water contact angle (WCA) and improving the hydrophilicity of COF-CDN membranes (Fig. 3e). Consequently, the WCA of the COF-CDN-12 membrane decreased sharply from 51.1° to 10.9° within 10 s, whereas the WCA of the pristine COF membrane only declined from 74.2° to 65.7° in the same time. Furthermore, the hourglass-shaped nanochannels, which consisted of the hydrophilic entrance and the hydrophobic spout, reduced flow resistance^24^, and thus allowed for fast water permeation and enhanced membrane wettability, exhibiting asymmetric wettability. To further investigate the effect of different pH values on the wettability of COF-CDN membranes, the WCA of the pristine COF and COF-CDN-12 membranes was measured at different pH values (Supplementary Fig. 14 and Fig. 3f-h). The WCA of pristine COF membrane showed almost no change at pH values of 3, 7, and 11 (Supplementary Fig. 14). In contrast, the WCA of COF-CDN-12 membrane increased with the pH value, reaching approximately 48.5°, 51.1°, and 57.4° at pH values of 3, 7, and 11 (Fig. 3f-h). The increase in WCA with rising pH values was attributed to changes in the membrane surface chemistry and charge distribution. At lower pH values, the amino groups present in the COF-CDN membrane were protonated, which increased the surface hydrophilicity. As the pH increased, these amino groups deprotonated, reducing the surface affinity for water and rendering higher WCA values. Furthermore, deprotonation probably caused a shift in surface charge density and a decrease in electrostatic interactions between the membrane surface and water molecules, making the surface less hydrophilic when the aqueous solution became more alkaline.

In addition, we conducted zeta potential measurements to evaluate the surface charge behavior of COF-CDN membranes (Fig. 3i). Based on the observed changes in zeta potential, we concluded that the membrane surface charge shifts from positive at low pH value to negative at high pH value. This indicated the protonation and deprotonation of amino groups, confirming the membrane pH-responsivity and providing insights into its potential performance in separation processes under different pH values. Simultaneously, with the increase of CDN, the changes in zeta potential became more pronounced, and the isoelectric point (IEP) shifted in accordance with the CDN content. This phenomenon can be attributed to the influence of CDN in altering the surface charge characteristics of the membrane. Due to the presence of amino groups involved in charge regulation, the IEP shifted, resulting in more pronounced changes in zeta potential at different pH values.

### Desalination performance of COF-CDN membranes

**Fig. 4:**
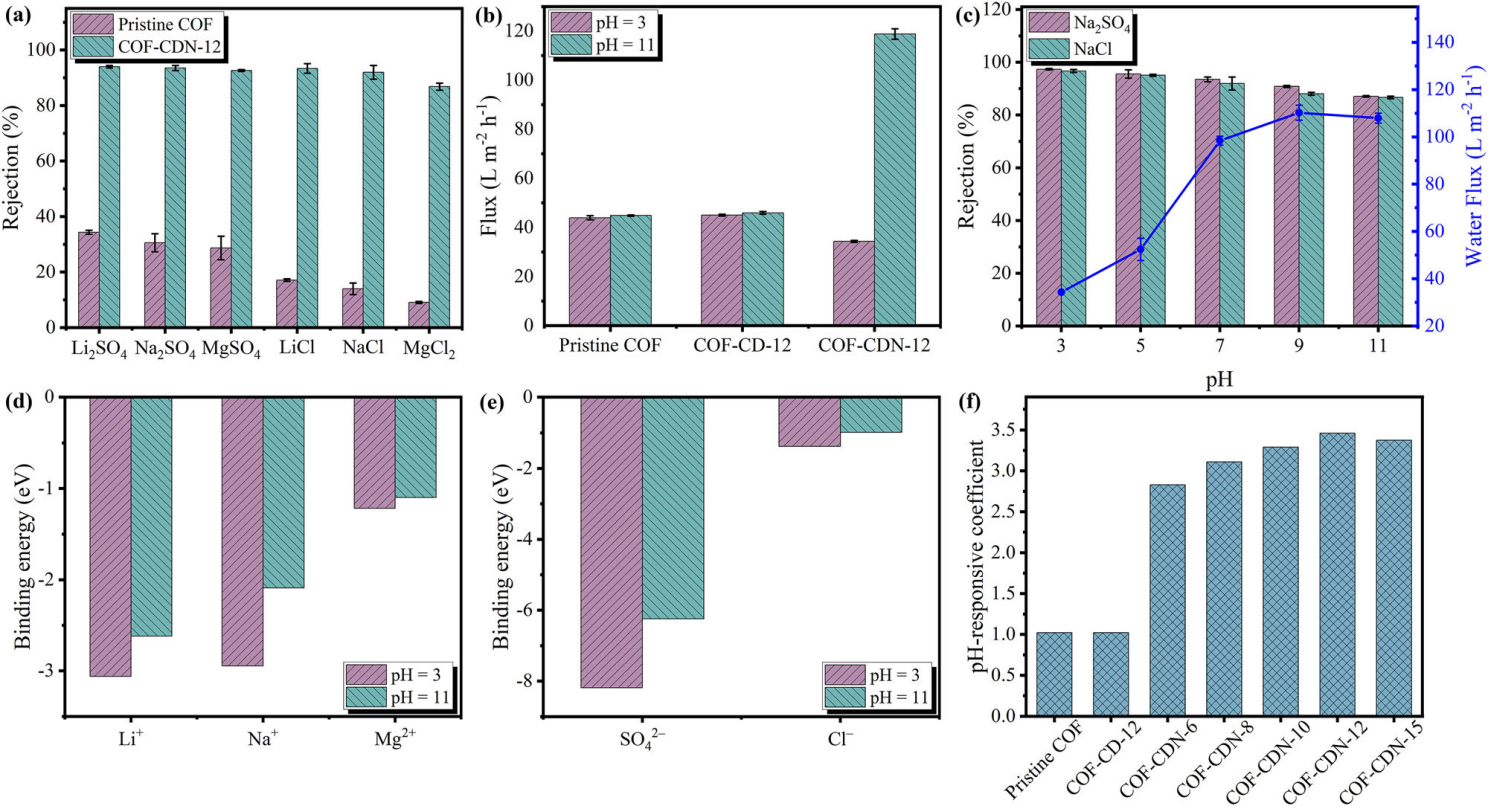
Performance of COF-CDN membranes. **a** The desalination performance of pristine COF and COF-CDN-12 membranes at pH value of 7; **b** the flux (non-normalized, measured at 2.0 bar) of pristine COF membrane, COF-CD-12 membrane and COF-CDN-12 membrane at pH values of 3 and 11; **c** the desalination performance of COF-CDN-12 membranes at pH values of 3 and 11; Error bars represent the standard deviation from three independent experiments. Binding energy between **d** cations and **e** anions and CDN at different pH values calculated by DFT; **f** the pH-responsive coefficient for water flux of COF-CDN membranes. The pH-responsive coefficient was defined as the ratio of the water flux at a pH value of 11 to that at a pH value of 3^47,48^.

**Fig. 5:**
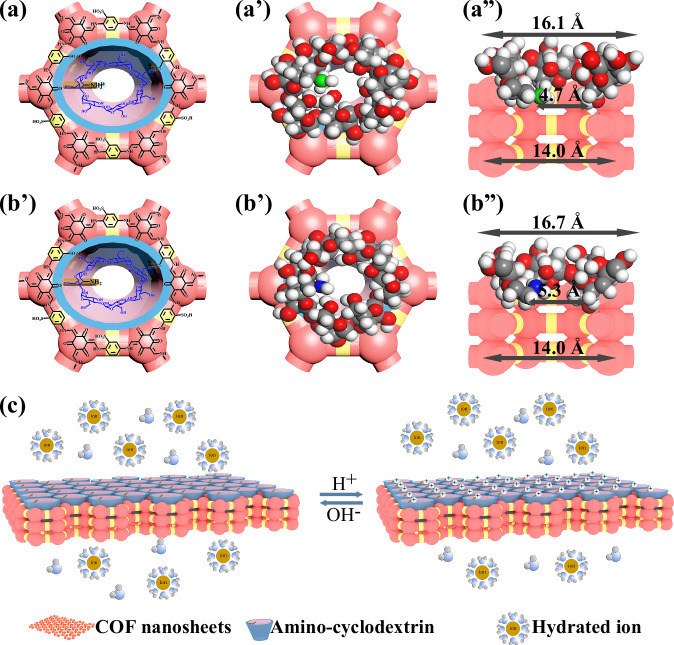
Hourglass-shaped nanochannels structure of COF-CDN membranes. **a** Protonated and **b** deprotonated states of the hourglass-shaped nanochannels. The colors of the atoms are represented as follows: gray for carbon (C), white for hydrogen (H), red for oxygen (O), blue for nitrogen (N), and green for positively charged nitrogen (N^+^). Channel sizes were calculated using Materials Studio simulation, with specific measurements provided in (a”) and (b”); **c** pH-responsive protonation-deprotonation transition behavior of the COF-CDN membranes.

The water flux (non-normalized, measured at 2.0 bar) and salt rejection of COF membrane with hourglass-shaped nanochannels (COF-CDN) were investigated by dead-end filtration cell (Supplementary Fig. 16). First, we evaluated the desalination performance using a series of membranes with different CDN contents in pure water and aqueous solutions of six different salts (Li_2_SO_4_, Na_2_SO_4_, MgSO_4_, LiCl, NaCl and MgCl_2_) at a constant operating pressure of 2.0 bar, as shown in Fig. 4a and Supplementary Fig. 17. The pristine COF membrane exhibited a water flux of 45 L m^–2^ h^–1^ (Supplementary Fig. 17), with a low rejection of less than 40% (Fig. 4a and Supplementary Fig. 17). Such a low rejection of pristine COF membrane was attributed to the size sieving, as the hydration diameters of salt ions (Supplementary Table 1) were smaller than the intrinsic nanopore size of the TpPa-SO_3_H COF nanosheets (1.4 nm). In contrast, COF-CDN membranes, such as COF-CDN-12 membrane, exhibited significantly increased permeance and rejection, with a water flux of 98 L m^–2^ h^–1^ (Supplementary Fig. 17) and rejection over 90% (Fig. 4a and Supplementary Fig. 17). The high-water flux of COF-CDN membranes resulted from a combination of structural features and improved surface properties. As shown in Fig. 1 and Fig. 5, the resulting hetero-nanochannels consisted of CDN-specific cavity (hydrophilic conical entrance and hydrophobic spout) and COF with intrinsic nanopores (homo-nanochannels). The hydrophilic conical entrance facilitated the entry of water by lowering the critical intrusion pressure, while the gradually narrowing geometry guided flow and prevented turbulence. The hydrophobic spout, together with the homo-nanochannels, created a slip boundary that minimized viscous friction, thereby enabling low-resistance and fast water transport^18^. At the same time, the increased membrane hydrophilicity (Fig. 3e) and surface roughness (Supplementary Fig. 12) provided additional interaction sites, while hydroxyl groups in CDN added more polar sites and fortify water–membrane interactions, collectively reducing resistance, allowing water molecules to spread more easily, and increasing membrane permeance. As shown in Supplementary Fig. 18, the flux recovery rate (FRR) exceeded 90% for both humic acid (HA) and bovine serum albumin (BSA), demonstrating excellent antifouling performance. Notably, the introduction of CDN further improved the antifouling properties, with the FRR of the COF-CDN-12 membrane reaching 94.1% (HA) and 95.8% (BSA). The synergy between the membrane surface structure and its hydrophilicity facilitated water permeation, ultimately resulting in higher water flux. The outstanding rejection of COF-CDN membranes was attributed to the hydrophobic spout of the CDN cavity diameter (~0.5 nm) smaller than the hydration diameters of salt ions (Supplementary Table 1). The membrane exhibited consistent ion rejection performance in a mixed salt solution (NaCl and MgCl_2_, 1000 ppm each), comparable to that observed in single salt solutions (Supplementary Fig. 19). Building upon this, a two-stage filtration test was performed to further evaluate its potential under more realistic conditions. After two successive filtrations, the salt concentration in the final permeate was reduced from 1000 ppm to approximately 3 ppm with total salt rejection of 99.7%, meanwhile, our COF-CDN membrane possesses a water flux of 98 L m^–2^ h^–1^ measured at 2.0 bar. This result demonstrates that the COF-CDN membrane achieves deep desalination with high water flux when operated in a staged configuration, indicating its promise for practical application. After soaking in 100 ppm sodium hypochlorite solution for 24 h, the membrane preserves its structural integrity (FT-IR, Supplementary Fig. 20) and maintains stable desalination performance (with water flux and salt rejection are consistent with those before soaking, Supplementary Fig. 21). This was attributed to the chemically stable framework of COF nanosheets, which lacked oxidation-prone amide groups commonly found in polyamide membranes, that were susceptible to chlorine attack^41,42^. The π-conjugated aromatic framework of the COF nanosheets provided oxidative robustness, which remained stable under hypochlorite conditions^30,43^. The FT-IR spectra showed no signs of bond cleavage or functional group degradation, supporting the structural integrity of both the COF and the CDN. These results confirmed that the COF-CDN membrane exhibited excellent chlorine tolerance.

### pH-responsive property of COF-CDN membranes

The amino groups on CDN in COF-CDN membranes endow the nanochannels with pH-responsive property, allowing dynamic regulation of their effective size and charge. Given the significance of this pH-responsive behavior in membrane performance, the water flux (non-normalized, measured at 2.0 bar) and rejection for Na_2_SO_4_ and NaCl under different pH values were investigated at a constant operating pressure of 2.0 bar to evaluate the pH-responsive property of COF-CDN membranes (Fig. 4b, c and Supplementary Figs. 22, 23). For the pristine COF membrane without installing CDN at the nanochannel mouth, the water flux of 45 L m^–2^ h^–1^ and rejection of 31% for Na_2_SO_4_ and 14% for NaCl remained unchanged at pH values of 3 and 11, indicating that the pristine COF membrane lacked pH-responsive property (Fig. 4b). Similarly, the COF-CD-12 membrane by installing CD without amino groups onto the mouth of COF membrane did not exhibit pH-responsive property in water flux and rejection for Na_2_SO_4_ and NaCl (Fig. 4b and Supplementary Fig. 24). As shown in Fig. 4b, c, the COF-CDN-12 membrane exhibited the tunable water flux and rejection for NaCl under different pH values, as pH value increased from 3 to 11, the water flux was notably increased from 34 L m^–2^ h^–1^ to 108 L m^–2^ h^–1^, while rejection for NaCl was decreased from 97% to 87%. In addition, the water flux and rejection for Na_2_SO_4_ of the COF-CDN-12 membrane, as well as other COF-CDN membranes (Supplementary Fig. 22-23), exhibited pH-responsive properties at different pH values. Under acidic conditions, the amino groups in CDN were protonated, causing the CDN specific cavity to shrink^44^, with the hydrophilic conical entrance size of 16.1 Å and the hydrophobic spot size of 4.7 Å (Fig. 5a). Consequently, the pore of COF-CDN membranes either decreased in effective size or entered a “closed” state, resulting in lower water flux (34 L m^–2^ h^–1^) and higher salt rejection (97%) (Fig. 5c). Additionally, the positively charged membrane surface (Fig. 3i), resulting from protonated amino groups, induced a pronounced Donnan exclusion effect. This electrostatic phenomenon was particularly significant for multivalent anions such as SO_4_^2−^, which experienced stronger repulsive forces compared to monovalent anions like Cl^−^. The initial attraction of divalent anions to the positively charged membrane surface was proposed to result in the formation of a concentrated counterion layer, which subsequently amplified the repulsion of additional incoming anions from the solution. This mechanism was considered to contribute to the consistently higher rejection of Na_2_SO_4_ relative to NaCl under acidic conditions. Under neutral or alkaline conditions, the amino groups in CDN were deprotonated, leading to the extension of the CDN specific cavity^45,46^, with the hydrophilic conical entrance size of 16.7 Å and the hydrophobic spot size of 5.3 Å (Fig. 5b). Consequently, the pore of COF-CDN membranes either increased in effective size or entered an “open” state, resulting in higher water flux (108 L m^–2^ h^–1^) and lower salt rejection (87%) (Fig. 5c). Despite the reduced positive charge at pH value of 11 (Fig. 3i), the rejection for Na_2_SO_4_ remained higher than for NaCl due to the larger hydration diameter and multivalent nature of the SO_4_^2−^ ions (Supplementary Table 1). These characteristics contributed to greater steric hindrance and resistance to transport through the nanochannels compared to monovalent Cl^−^ ions, maintaining higher rejection for Na_2_SO_4_.

To further account for the energy barriers introduced by CDN-ion interactions, DFT calculations were conducted to quantify the binding energy between the membrane and various salt ion. As shown in Fig. 4d, e, the influence of pH on membrane performance became particularly evident when comparing the two states. Under acidic conditions (pH = 3), the amino groups on CDN were protonated, thereby fortifying ion–membrane interactions. The calculated binding energies under pH 3 were –3.06 eV for Li^+^, –2.95 eV for Na^+^, –1.22 eV for Mg^2+^, –8.19 eV for SO_4_^2−^, and –1.38 eV for Cl^−^. The enhanced affinity raised the energy barrier for ion passage and contributed to improving ion rejection. Conversely, under alkaline conditions (pH = 11), the amino groups were deprotonated, which weakened these interactions and effectively lowered the steric and electrostatic resistance within the nanochannels. As a result, ions were more readily transported through the membrane. The calculated binding energies under pH 11 were –2.62 eV for Li^+^, –2.09 eV for Na^+^, –1.10 eV for Mg^2+^, –6.24 eV for SO_4_^2−^, and –0.98 eV for Cl^−^. Moreover, the binding energies between CDN and the cations consistently followed the order Li^+^ > Na^+^ > Mg^2+^ (Fig. 4d) at both pH 3 and pH 11, whereas for anions, the order was SO_4_^2− ^> Cl^−^ (Fig. 4e). This binding hierarchy aligns well with the experimentally observed rejection sequence, indicating that ions exhibiting stronger affinity toward the membrane were more effectively retained. Meanwhile, DFT calculations considering both cation and anion interactions under protonated and deprotonated states indicated that ion rejection was controlled by interactions involving both cations and anions, rather than only by Donnan exclusion (Supplementary Fig. 25). The MD simulation results (Supplementary Fig. 26a) provided visualized evidence of water and salt transport through hourglass-shaped nanochannels. While water diffusion was reduced in the protonated state compared to the deprotonated state, no salt ion transport was detected under either condition. The interactions between water/ions and the functional groups as they passed through the hourglass-shaped nanochannels formed by COF-CDN were analyzed (Supplementary Fig. 26b, c). Stronger interactions (i.e., more negative values) in the protonated state hindered water/salt molecular transport, which accounted for the experimentally observed low water flux and high salt rejection under acidic conditions.

To quantitatively evaluate the pH-responsive property of COF-CDN membranes, a pH-responsive coefficient was defined as the ratio of the water flux (Fig. 4d) or Na_2_SO_4_ rejection (Supplementary Fig. 27) at a pH value of 11 to that at a pH value of 3^47,48^. The pH-responsive coefficient for both water flux and Na_2_SO_4_ rejection of the pristine COF membrane and COF-CD-12 membrane was merely 1, indicating that these parameters was almost independent of pH value (Fig. 4d and Supplementary Fig. 22). With increasing CDN content, the pH-responsive coefficient for water flux gradually increased from 2.8 for the COF-CDN-6 membrane to 3.5 for the COF-CDN-12 membrane, and then decreased to 3.4 for the COF-CDN-15 membrane (Fig. 4d). This was because the separation layer of the COF-CDN-15 membrane became thicker (Supplementary Fig. 9), and dense interfacial assembly tended to cause partial misalignment of the hourglass-shaped nanochannels, leading to a decrease in membrane water flux (Supplementary Fig. 17). The flux was normalized by multiplying it with membrane thickness to eliminate the effects of thickness variations^49^. With the increase of CDN content, the formation of hourglass-shaped nanochannels was enhanced, thereby facilitating fast and low-resistance water transport, as confirmed by monotonically increasing thickness-normalized flux (Supplementary Fig. 28), despite the increased membrane thickness and partial misalignment of the hourglass-shaped nanochannels. However, with increasing CDN content, the pH-responsive coefficient for Na_2_SO_4_ rejection decreased, indicating that the rejection was dependent on pH value (Supplementary Fig. 27). The results indicated that the amino groups in CDN successfully conferred pH-responsive property to COF-CDN membranes, with the ability to regulate the membrane effective size increasing as the CDN content increased.

### Stability of COF-CDN membranes

**Fig. 6:**
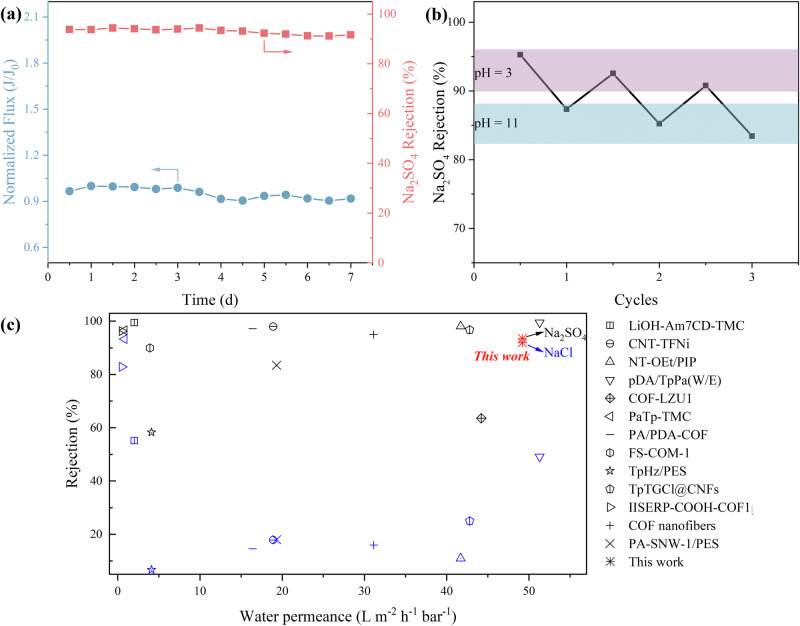
Stability of COF-CDN membranes. **a** Long-term testing of COF-CDN-12 membrane for Na_2_SO_4_ solution using a cross-flow filtration cell at a constant operating pressure of 2.0 bar; **b** Na_2_SO_4_ rejection of COF-CDN-12 membrane when the pH shifted from 3 to 11 after 3 cycles; **c** the performance of COF-CDN-12 membrane, as compared to the state-of-the-art membranes (Supplementary Table 2). The red point represents this work, while the black and blue points indicate the rejection of Na_2_SO_4_ and NaCl, respectively.

To evaluate the stability of COF-CDN membranes, long-term performance and pH cycling tests were conducted (Fig. 6 and Supplementary Figs. 29, 30). The operational stability of the COF-CDN-12 membrane was assessed through long-term testing in a Na_2_SO_4_ solution using a cross-flow filtration cell (Fig. 6a). Throughout the 7-day testing period, the membrane exhibited stable flux and consistent rejection, consistently achieving over 90% rejection for Na_2_SO_4_. The surface and cross-sectional SEM images of COF-CDN-12 membrane after long-term testing showed a relatively uniform surface, indicating that the membrane maintained its integrity without significant damage or fouling (Supplementary Fig. 29). The preserved performance over extended operation demonstrated that the COF-CDN-12 membrane possessed excellent stability and reliable selectivity. Furthermore, after three cycles of pH value shifting from 3 to 11, the COF-CDN-12 membrane maintained consistent water flux, with no significant decrease in performance observed at either pH value of 3 or 11, as shown in Supplementary Fig. 30a. In contrast, as shown in Fig. 6b and Supplementary Fig. 30b-d, rejection for Li_2_SO_4_, Na_2_SO_4_, LiCl, NaCl exhibited a slight decline at either pH value of 3 or 11, due to partial retention of salt ions within the membrane pores^50,51^. The combination of water flux stability and the limited rejection decrease demonstrated the reversible and reliable pH-responsive property of the COF-CDN-12 membrane, confirming its robust structural integrity, which was crucial for maintaining stable separation under fluctuating pH values. The combination of long-term operational stability and reversible pH-responsive underscores the membrane capability to maintain optimal separation while resisting performance degradation. Moreover, the COF-CDN-12 membrane exhibited the enhanced separation performance compared to the state-of-the-art membranes reported in the literature (Fig. 6c and Supplementary Table 2). Its water flux and ion rejection surpass those of many existing desalination membranes, breaking the trade-off between membrane permeability and selectivity and positioning it at the forefront of current desalination membranes. These findings indicate the applicability of the COF-CDN membrane in separation processes requiring dynamic control, such as smart filtration.

## Discussion

In this study, a kind of COF membrane with hourglass-shaped nanochannels is designed and fabricated. The hydrophilic conical entrance facilitates the entry of water molecules, whereas the hydrophobic spout and the homo-nanochannels collectively enable fast water transport. Meanwhile, the amino groups on CDN endow the hetero-nanochannels with pH-responsive ability to dynamically regulate their effective size and charge: as the pH value shifts from 11 to 3, the effective size decreases from 5.3 Å to 4.7 Å, and the hetero-nanochannels switch from neutral to positively charged. As a result, the optimum membrane, COF-CDN-12 membrane, exhibited a high-water flux of 98 L m^–2^ h^–1^ and rejection of over 90% for various salt solutions, much higher than those of the pristine COF membrane. Moreover, the membrane exhibits superior operational stability (7 days) and pH cycle stability. Our study provides an efficient route to constructing hetero-nanochannels in COF membranes, breaking the trade-off between membrane permeability and selectivity in various chemical separations.

## Methods

### Synthesis of COF nanosheets

The nanosheets used for the preparation of COF membranes with hourglass-shaped nanochannels were synthesized by a two-phase method. 21.0 mg of Tp was added into octanoic acid (20.0 mL) to form solution A (oil phase). 28.2 mg of Pa-SO_3_H was added to deionized water (30.0 mL) to form solution B (water phase). Then, solution A was carefully added dropwise onto the top of solution B, and the reaction was kept under static conditions at 20 °C for 7 days. The collected water phase was dialyzed in deionized water for 3 days, yielding TpPa-SO_3_H COF nanosheets with a final concentration of 1.0 mg mL^–1^.

### Preparation of COF-CDN membranes

0.9 mL of COF nanosheets dispersion was diluted in 10.0 mL of ethanol and then assembled onto a nylon substrate membrane under a pressure of 0.4 bar to prepare a pristine COF membrane. Next, a specific volume of CDN was assembled onto the mouth of COF membrane under a pressure of 0.4 bar to prepare a COF-CDN-X membrane, where *X* refers to the volume content of CDN relative to COF nanosheets (*X* = 6, 8, 10, 12, 15). Additionally, 12 mL of β-CD (CD) was assembled onto the mouth of COF membrane under the same pressure to prepare the COF-CD-12 membrane. The resultant pristine COF, COF-CD-12, and COF-CDN-X membranes were left to dry at room temperature for no less than 8 h before performing the tests. The entire process proceeds under ambient temperature and modest vacuum pressure without specialized equipment, making it readily adaptable to larger substrates.

### Performance of COF-CDN membranes

The performance of COF membranes with hourglass-shaped nanochannels in terms of water flux and salt rejection was evaluated using 1000 mg L^−1^ solutions of Li_2_SO_4_, Na_2_SO_4_, MgSO_4_, LiCl, NaCl and MgCl_2_, employing a dead-end filtration cell (Supplementary Fig. 16b). For the pH-responsive property, solutions of Na_2_SO_4_ and NaCl at pH values of 3, 5, 7, 9, and 11 were prepared, with the pH adjusted to the desired using 1 M HCl and NaOH. The test was conducted with pressure provided by high-purity N_2_, and the airtightness of the system was confirmed before starting the test. To ensure stable permeance, the system was operated for 0.5 h at 2.5 bar, and then tested at 2.0 bar. The water flux (J, L h^−1^ m^−2^) and rejection (R, %) were calculated using the following:

1 $$J=\frac{V}{A\times \Delta t}$$

2 $$R=\left(1-\frac{{C}_{p}}{{C}_{f}}\right)\times 100\%$$

Where *V* (L), *A* (m^2^), and Δ*t* (h) were the permeate fluid volume, the effective filtration area and the operation time; *C*_*p*_ (mg L^–1^) and *C*_*f*_ (mg L^–1^), the concentrations of the filtrate and feed solutions, were measured using an ion chromatography system (IC, Dionex ICS-600).

In this study, all water flux was reported as non-normalized flux (L h^−1^ m^−2^) under a constant pressure of 2.0 bar, unless otherwise specified. Normalized flux was calculated and used solely for performance interpretation in specific sections, such as Fig. 6a for the evaluation of stability, Supplementary Fig. 18 for the evaluation of antifouling properties, and Supplementary Fig. 28 for the elimination of membrane thickness variation effects.

To evaluate the stability of the membrane, a long-term test was conducted using a cross-flow filtration cell (Supplementary Fig. 16a). During the experiment, the flow velocity was maintained at 72 mL h^−1^, and the effective filtration area was 2.01 × 10^−4^ m^2^. A 1000 mg L^–1^ Na_2_SO_4_ solution (1 L) was used, with periodic replenishment to maintain a constant volume. The experiment was carried out at an operating pressure of 2.0 bar over 7 days, with filtrate volume and concentrations recorded every 12 h to determine flux and rejection.

## Supplementary information

Supplementary information

Transparent Peer Review file

## Source data

Source data

## Data Availability

All data supporting the findings of this study are available within the article and the Supplementary Information file, or available from the corresponding authors upon request. Source data are provided with this paper.
