# Supplementary material for: Covalent organic framework membrane with hourglass-shaped nanochannels for ultrafast desalination
Source: Nat Commun. 2025 Aug 30;16:8125. doi: 10.1038/s41467-025-63650-5 (PMC12398615; doi:10.1038/s41467-025-63650-5)
Supplement: Supplementary file 1 — Supplementary information [file 41467_2025_63650_MOESM1_ESM.pdf]

Supplementary Information

Covalent organic framework membrane with hourglass-shaped nanochannels for ultrafast desalination

Xiaocui Wei[a], Yanan Liu[a*], Fu Zhao[a], Tingyuan Wang[a], Zongmei Li[a], Chunyang Fan[a], Yuhan Yang[a], Yuhan Wang[a], Zhongyi Jiang[a,b*]

[a] School of Chemistry and Chemical Engineering, Collaborative Innovation Center of Ecological Civilization, Hainan University, Haikou, 570228, China

[b] Key Laboratory for Green Chemical Technology of Ministry of Education, School of Chemical Engineering and Technology, Tianjin University, Tianjin 300072, China

[*] Corresponding author. School of Chemistry and Chemical Engineering, Hainan University, Haikou, 570228, China

E-mail: zhyjiang@tju.edu.cn; liuyanan@hainanu.edu.cn

Supplementary Information content:

Supplementary Text

Number of pages: 44 (S1 to S44)

Number of figures: 30 (Supplementary Fig. 1 to Supplementary Fig. 30)

Number of tables: 2 (Supplementary Table 1 to Supplementary Table 2)

References

Supplementary Figures and Tables:

Supplementary Figure 1. Synthesis route and chemical structures of COF nanosheets.

Supplementary Figure 2. FT-IR spectra of COF nanosheets, Tp, and Pa-SO_3H.

Supplementary Figure 3. XRD pattern of the COF powder.

Supplementary Figure 4. AFM image and height data of COF nanosheets.

Supplementary Figure 5. TGA curves of COF nanosheets.

Supplementary Figure 6. SEM image of the Nylon substrate membrane.

Supplementary Figure 7. Surface SEM images of difference membranes.

Supplementary Figure 8. Cross-sectional SEM images of difference membranes.

Supplementary Figure 9. The thickness of difference membranes.

Supplementary Figure 10. SEM image of the COF-CD-12 membrane.

Supplementary Figure 11. 2D and 3D AFM images of difference membranes.

Supplementary Figure 12. The surface roughness of difference membranes.

Supplementary Figure 13. FT-IR spectra of COF-CDN membranes.

Supplementary Figure 14. The WCA of pristine COF membrane at different pH values.

Supplementary Figure 15. Zeta potential of the Nylon substrate membrane.

Supplementary Figure 16. Schematic illustration of the filtration cell.

Supplementary Figure 17. Desalination performance of different membranes.

Supplementary Figure 18. Time-dependent normalized flux of different membranes.

Supplementary Figure 19. Rejection of COF-CD-12 membrane in different conditions.

Supplementary Figure 20. FT-IR spectra of COF-CDN-12 membrane before and after treatment with hypochlorite solution.

Supplementary Figure 21. Desalination performance of the COF-CDN-12 membrane after treatment with sodium hypochlorite solution.

Supplementary Figure 22. Water flux of COF-CDN membranes at different pH values.

Supplementary Figure 23. Rejection of COF-CDN membranes at different pH values.

Supplementary Figure 24. Rejection of COF-CD-12 membrane at different pH values.

Supplementary Figure 25. Binding energy calculated by DFT.

Supplementary Figure 26. MD simulations the hourglass-shaped nanochannels.

Supplementary Figure 27. pH-responsive coefficient for rejection of membranes.

Supplementary Figure 28. Thickness-normalized water flux of different membranes.

Supplementary Figure 29. Morphology of the membrane after long-term testing.

Supplementary Figure 30. Flux and rejection of the membrane after three pH cycles.

Supplementary Table 1. Hydrated diameter of salt ions.

Supplementary Table 2. Comparison of separation performance.

Supplementary Text

Materials and chemicals

1,3,5-Triformylphloroglucinol (Tp) was purchased from Jilin Chinese Academy of Sciences-Yanshen Technology Co., Ltd. 2,5-Diaminobenzenesulfonic acid (Pa-SO_3H) was obtained from Tokyo Chemical Industry Co., Ltd. 6-deoxy-6-amino-β-cyclodextrin (CDN) and β-cyclodextrin (CD) were gotten from Shandong Binzhou Zhiyuan Biotechnology Co., Lt. Octanoic acid, sodium hydroxide (NaOH), lithium sulfate (Li_2SO_4), sodium sulfate (Na_2SO_4), magnesium sulfate ($MgSO_4$), lithium chloride (LiCl), sodium chloride (NaCl), magnesium chloride ($MgCl_2$), humic acid (HA) and albumin from bovine serum (BSA) were all purchased from Shanghai Aladdin Biochemical Technology Co., Ltd. Nylon substrate membrane with a pore size of 0.2 μm was obtained from Tianjin Jinteng Experiment Equipment Co., Ltd. It was soaked in 1 M NaOH solution at 60°C for 1 h before use.

Synthesis of COF powders

The synthesis of COF powders was carried out using solvothermal reaction, with the following specific steps: 1,3,5-triformylphloroglucinol (Tp, 0.10 mmol), 2,5-diaminobenzenesulfonic acid (Pa-SO_3H, 0.15 mmol), 1,4-dioxane (8 mL), mesitylene (2 mL), and acetic acid (3 M, 0.1 mL) were mixed and added into a pyrex tube. After being sonicated for 0.5 h, the mixture was then frozen in liquid nitrogen. Subsequently, three freeze-pump-thaw cycles were performed for degassing treatment. The pyrex tube was sealed and then heated at 120°C for 3 d. When the reaction was completed, the red precipitate was collected from the pyrex tube, which was washed with acetone, ethanol, and deionized water to remove unreacted monomers. Finally, the precipitate was vacuum-dried at 120°C for 12 h to obtain COF powders.

Characterization of COF-CDN membranes

Fourier transform infrared (FT-IR, INVENIO, Germany) spectroscopy and solid-state ^{13}C nuclear magnetic resonance (ss-NMR, AVANCE III HD, Germany) provided insight into the chemical structure of the COF nanosheets. The crystallinity and

structural integrity of COF nanosheets were evaluated using X-ray diffraction (XRD, Mini Flex 600, Japan). Nitrogen adsorption-desorption isotherms (Micrometrics ASAP 2460, USA) further contributed to understanding the porosity of the COF nanosheets. The samples were activated by degassing at 120°C for 8 h, and the pore size distributions were derived from the sorption data using non-local density functional theory (NLDFT). Morphology of COF-CDN membranes was investigated using scanning electron microscopy (SEM, Verios G4 UC, USA), while the thickness of the COF nanosheets and roughness of COF-CDN membranes were assessed using atomic force microscopy (AFM, Bruker Dimension Icon, Germany). Detailed surface morphology of the COF nanosheets was evaluated using images obtained from transmission electron microscopy (TEM) and energy-dispersive X-ray spectroscopy (EDS) with Talos F200X G2 (USA). Thermal stability of the COF nanosheets were evaluated using thermogravimetric analysis measurements (TGA, HITACHI STA 200, Japan). Water contact angle (WCA, OCA 15EC, Germany) and Zeta potential (SurPASS, Austria) of COF-CDN membranes were also measured. The membranes after treatment in solutions at pH=3, 7, and 11 for 24 h were used to investigate the effect of different pH values on membrane wettability.

Antifouling properties of COF-CDN membranes

The antifouling properties of COF-CDN membranes were investigated using HA and BSA solution (1000 ppm). Initially, to ensure stable permeance, water was filtered through the membrane for 0.5 h at 2.5 bar, followed by another 0.5 h of water filtration at 2.0 bar, and the initial water flux was recorded as J_{w1} (L m^{-2} h^{-1}). Subsequently, a model foulant solution (HA or BSA) was filtered through the membrane for 1.0 h at 2.0 bar, and the resulting flux was recorded as J_p (L m^{-2} h^{-1}). Finally, the membrane that had been subjected to the foulant filtration test was cleaned with deionized water for 0.5 h, followed by another 0.5 h of water filtration at 2.0 bar, with the resulting flux recorded as J_{w2} (L m^{-2} h^{-1}). The antifouling properties were evaluated using the flux recovery rate (*FRR*), defined as follows:

$$FRR = \frac{J_{W2}}{J_{W1}} \times 100\% \quad (1)$$

Density functional theory calculation

Quantum chemical calculations were performed using the Gaussian 09W software package[1] based on density functional theory (DFT). All molecular geometries were fully optimized at the hybrid B3LYP functional level employing the 6-31G (d, p) basis set, without imposing symmetry constraints. The optimized geometries were confirmed as global minima by frequency calculations, which indicated the absence of imaginary frequencies. Additionally, dispersion interactions were accounted for using Grimme's D3 correction method (B3LYP+D3). The basis set superposition error (BSSE) was evaluated and corrected to accurately estimate intermolecular interaction energies.

Molecular dynamics simulations

All-atom molecular dynamics (MD) simulations were performed using the Gromacs-2023.6 software package[2] with the general AMBER force field[3] and RESP charges[4]. The simulated system was initialized as a relaxed liquid configuration at 298 K. Prior to the equilibrium MD simulations, energy minimization was conducted using a steepest descent algorithm with a convergence criterion set to a gradient of 100 kJ mol^{-1} nm^{-1}. Subsequently, the system underwent equilibration through a 3000 ps NVT simulation maintained at 298 K by employing a Nose´-Hoover thermostat[5]. Periodic boundary conditions were applied in all three spatial dimensions. Long-range electrostatic interactions were calculated using the Particle Mesh Ewald (PME) method[6,7] with a relative tolerance of 1×10^{-6} and a real-space cut-off distance of 1 nm. The van der Waals interactions were truncated at the same cut-off. Bond lengths involving hydrogen atoms were constrained using the LINCS algorithm[8]. A leap-frog integrator[9] with a 2×10^{-3} ps time step was used to advance the simulation. Additionally, a virtual wall was implemented along the z-direction to prevent water molecules from crossing the boundaries of the simulation box.

Supplementary Figures and Tables:

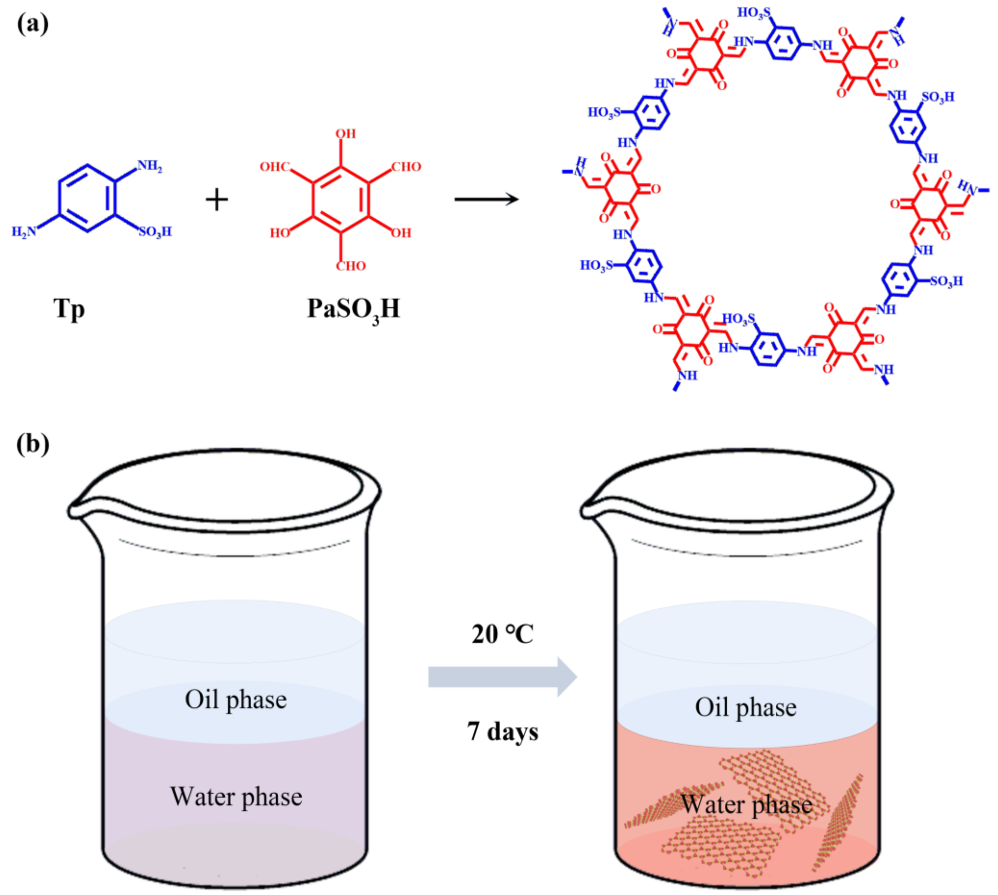

Supplementary Figure 1. Synthesis route and chemical structures of TpPa-SO_3H COF nanosheets.

Note: The nanosheets used for the preparation of COF membranes with hourglass-shaped nanochannels were synthesized by a two-phase method. Tp was added into octanoic acid to form solution A (oil phase). And Pa-SO_3H was added into deionized water to form solution B (water phase). Then, solution A was carefully added dropwise onto the top of solution B, and the reaction was kept under static conditions at 20°C for 7 d. The collected water phase was dialyzed in deionized water for 3 d, yielding TpPa-SO_3H COF nanosheets with a final concentration of 1.0 mg mL^{-1}.

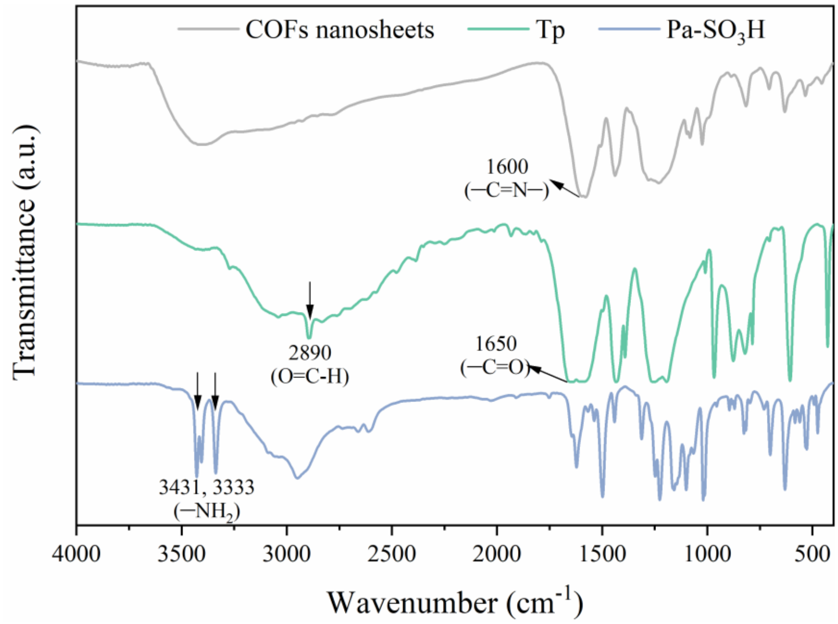

Supplementary Figure 2. FT-IR spectra of COF nanosheets, Tp, and Pa-SO_3H.

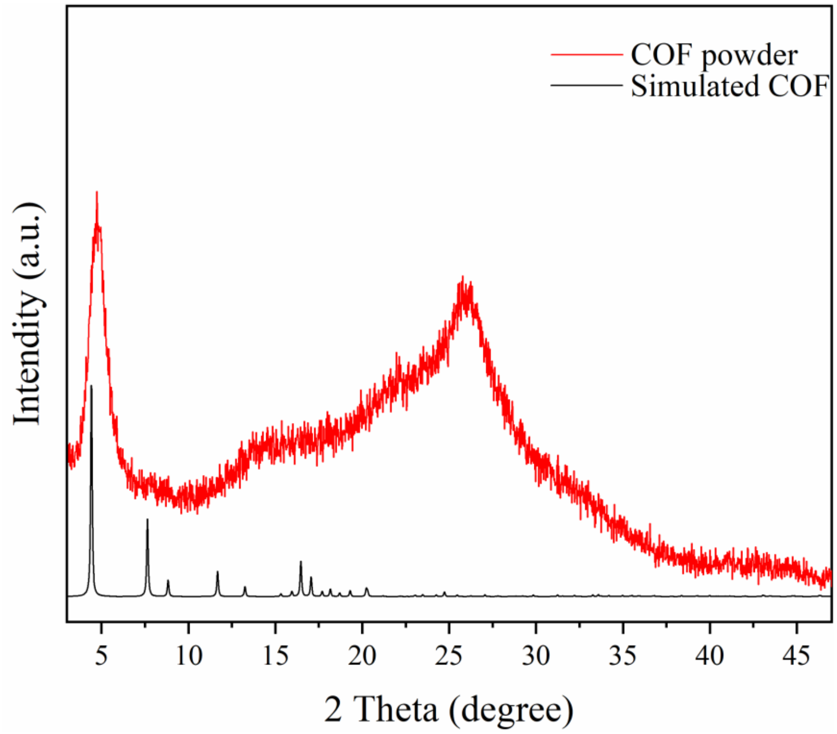

Supplementary Figure 3. XRD pattern of the COF powder synthesized by the solvothermal reaction.

Note: The red line represented the XRD pattern of TpPa-SO_3H COF powder, where prominent peaks were observed at 4.7° and 26.9°, corresponding to the (100) plane and the π-π stacking (001) plane, respectively. The slight deviation from the ideal crystal structure was consistent with previous literature[10]. The high relative intensity of the (100) plane indicated that the COF powder possessed a well-ordered crystalline structure. Furthermore, the positions of the (100) and (001) planes in the TpPa-SO_3H COF powder were consistent with the angles reported in the literature[11].

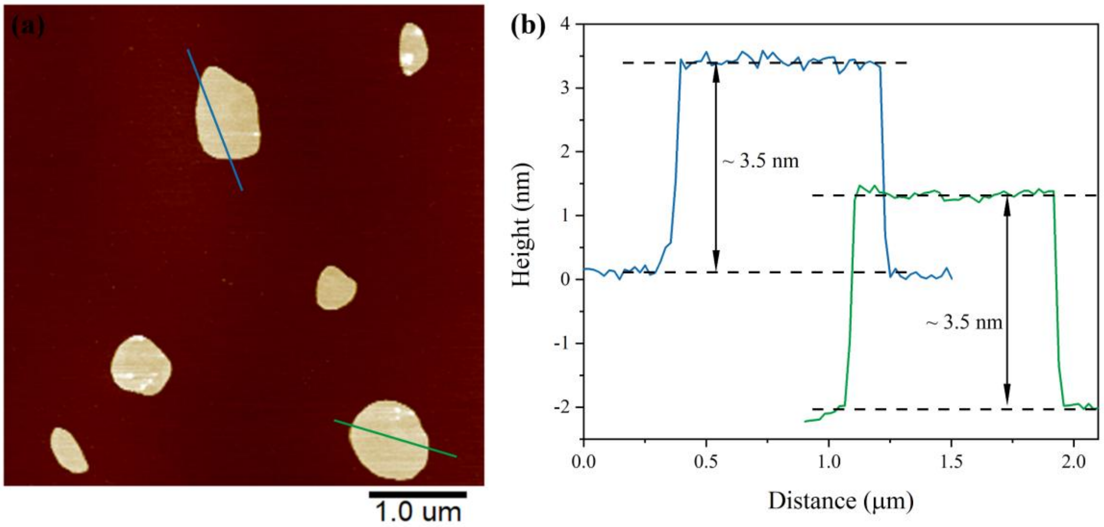

Supplementary Figure 4. (a) AFM image and (b) height data of TpPa-SO_3H COF nanosheets.

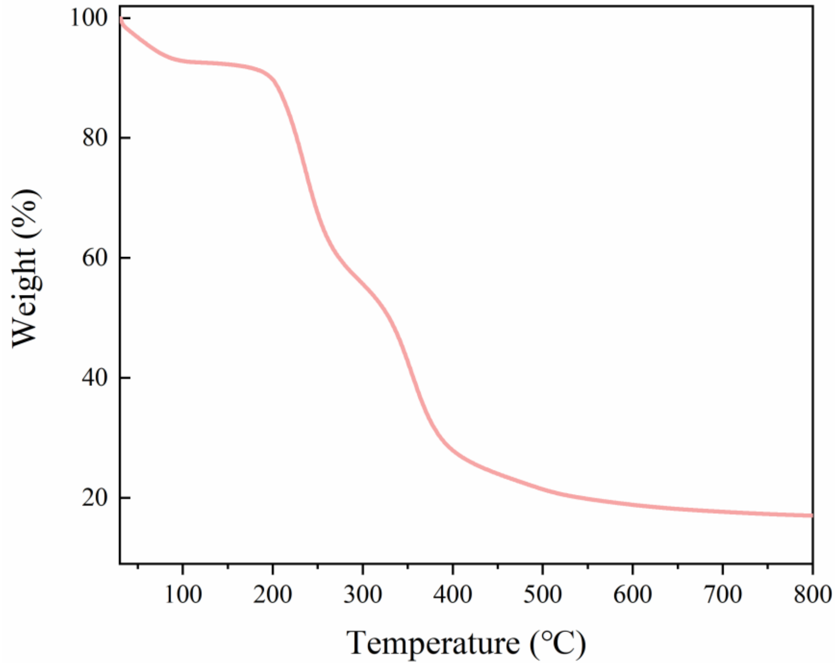

Supplementary Figure 5. TGA curves of TpPa-SO_3H COF nanosheets.

Note: The weight loss observed below 150 °C was attributed to the release of solvent molecules confined within the channels. TpPa-SO_3H COF nanosheets remained stable up to around 250 °C, demonstrating good thermal stability and indicating that the nanosheets maintained a stable chemical structure under heat.

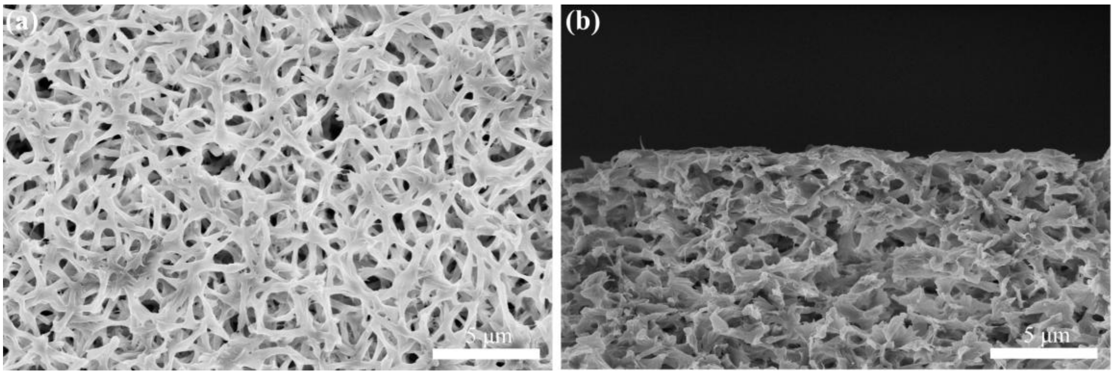

Supplementary Figure 6. (a) Surface SEM image and (b) cross-sectional SEM image of the Nylon substrate membrane.

Note: The Nylon substrate membrane, with an equivalent aperture of 0.2 μm, also featured surface cavities or pores in the micrometer range. The stacking and intertwining of fibers created a porous network, offering low-resistance pathways that facilitated high water flux through the membrane.

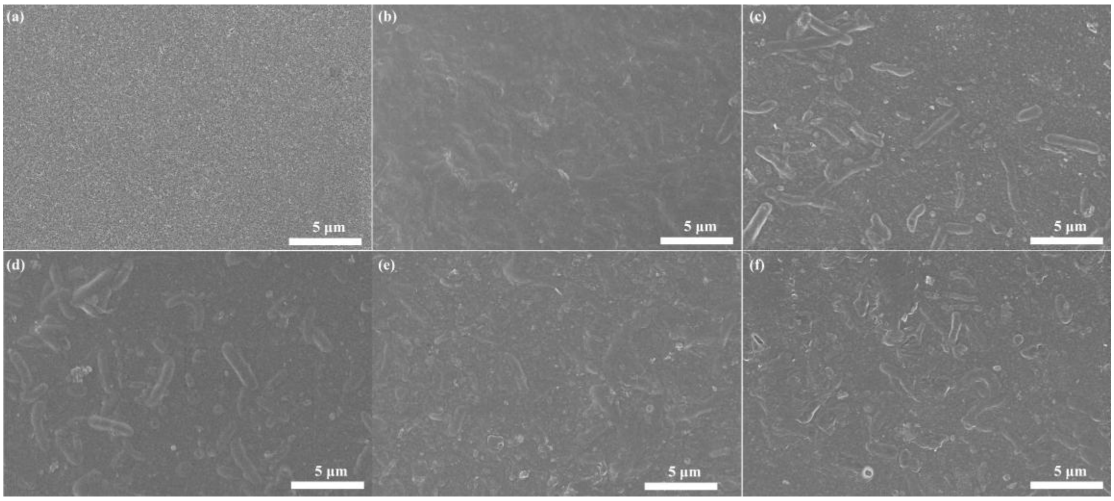

Supplementary Figure 7. Surface SEM images of pristine COF and COF-CDN membranes, including (a) pristine COF membrane; (b) COF-CDN-6 membrane; (c) COF-CDN-8 membrane; (d) COF-CDN-10 membrane; (e) COF-CDN-12 membrane; (f) COF-CDN-15 membrane.

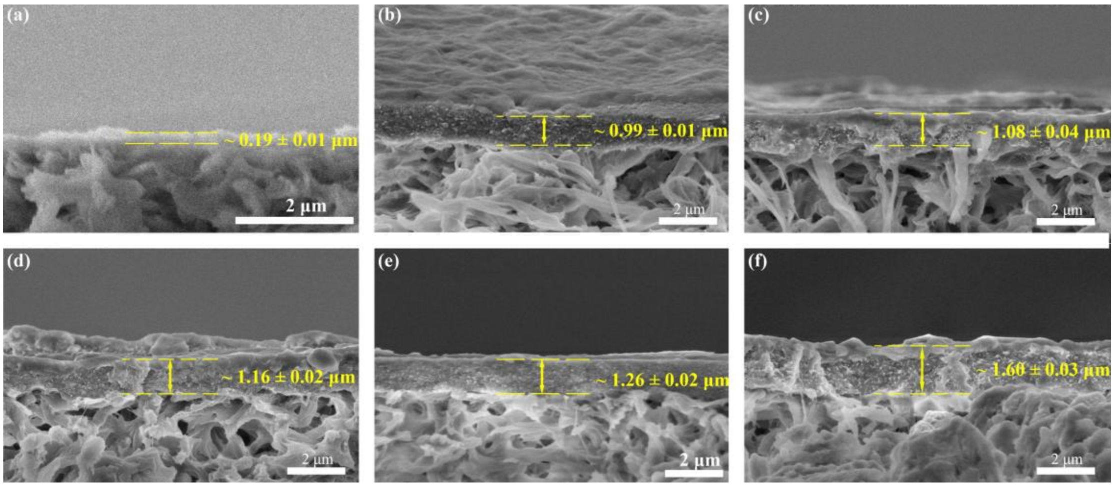

Supplementary Figure 8. Cross-sectional SEM images of pristine COF and COF-CDN membranes, including (a) pristine COF membrane; (b) COF-CDN-6 membrane; (c) COF-CDN-8 membrane; (d) COF-CDN-10 membrane; (e) COF-CDN-12 membrane; (f) COF-CDN-15 membrane.

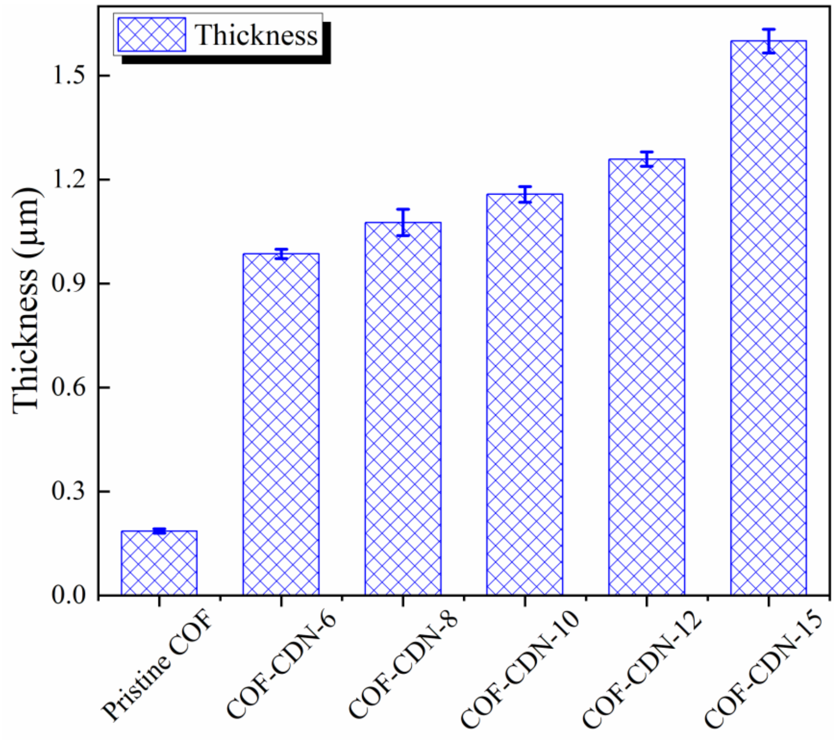

Supplementary Figure 9. The thickness of pristine COF membrane, COF-CDN-6 membrane, COF-CDN-8 membrane, COF-CDN-10 membrane, COF-CDN-12 membrane, and COF-CDN-15 membrane. Error bars represent the standard deviation from three independent experiments.

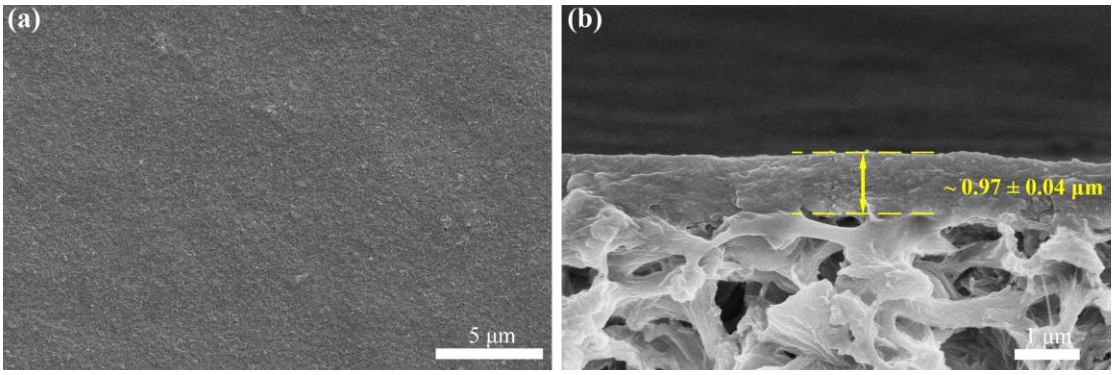

Supplementary Figure 10. (a) Surface SEM image and (b) cross-sectional SEM image of the COF-CD-12 membrane.

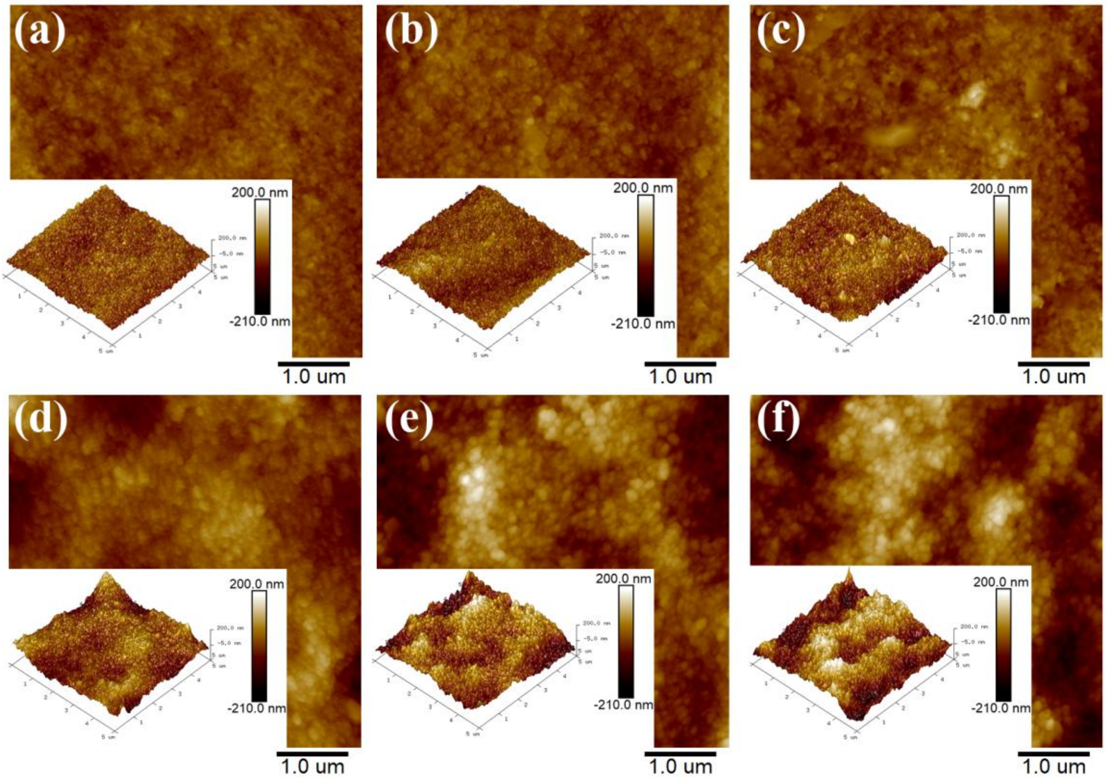

Supplementary Figure 11. 2D and 3D AFM images of pristine COF and COF-CDN membranes, including (a) pristine COF membrane; (b) COF-CDN-6 membrane; (c) COF-CDN-8 membrane; (d) COF-CDN-10 membrane; (e) COF-CDN-12 membrane; (f) COF-CDN-15 membrane.

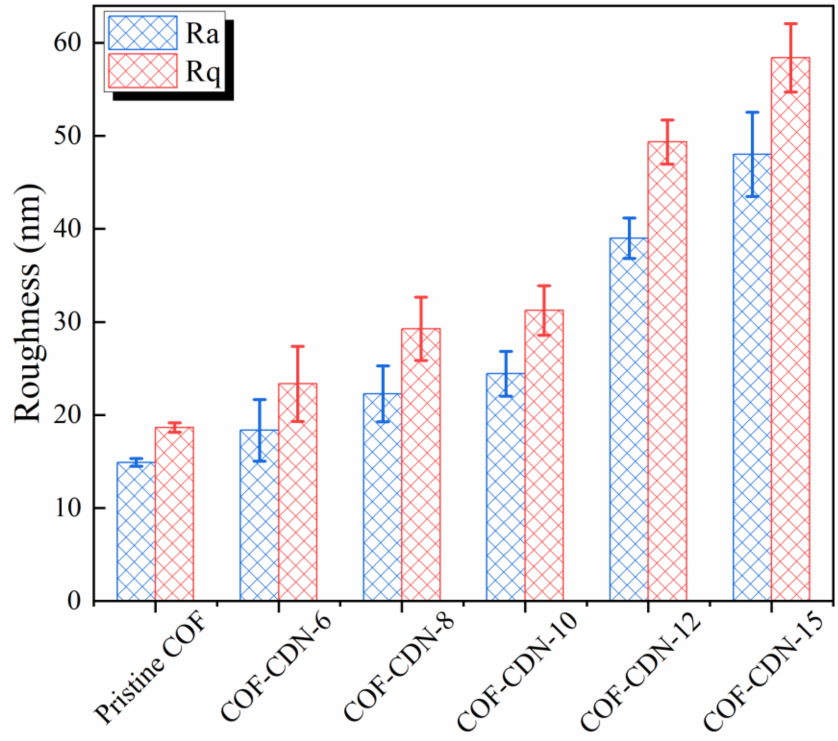

Supplementary Figure 12. The surface roughness of pristine COF membrane, COF-CDN-6 membrane, COF-CDN-8 membrane, COF-CDN-10 membrane, COF-CDN-12 membrane, and COF-CDN-15 membrane. Error bars represent the standard deviation from three independent experiments.

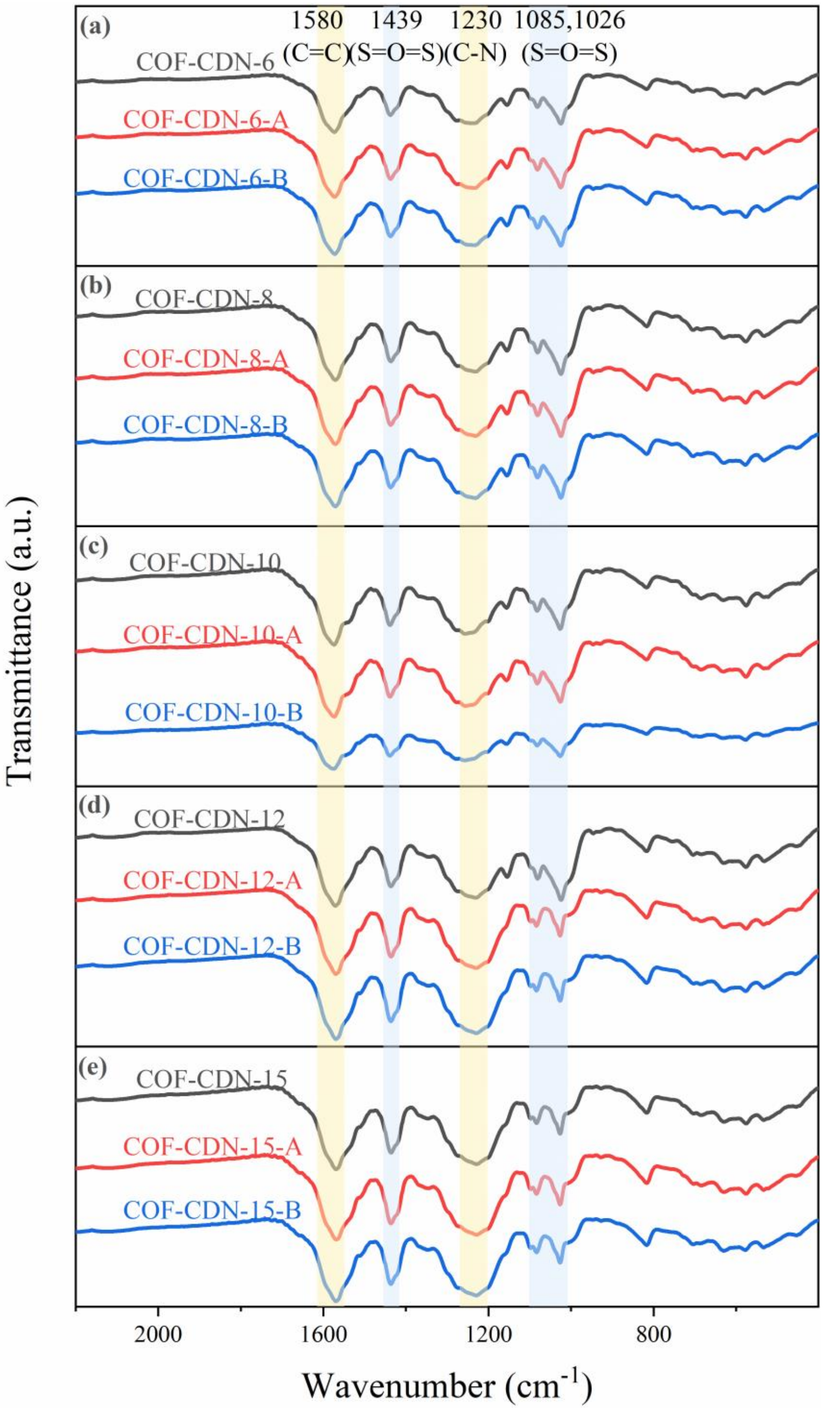

Supplementary Figure 13. FT-IR spectra of COF-CDN membranes after treatment with 1 M NaOH (COF-CDN-X-B) and HCl (COF-CDN-X-A) for 24 hours, including (a) COF-CDN-6 membrane; (b) COF-CDN-8 membrane; (c) COF-CDN-10 membrane; (d) COF-CDN-12 membrane; (e) COF-CDN-15 membrane.

Note: After the treatment, FT-IR spectra indicated that the functional groups remained intact, thereby demonstrating the excellent chemical stability of membrane under different pH environmental conditions.

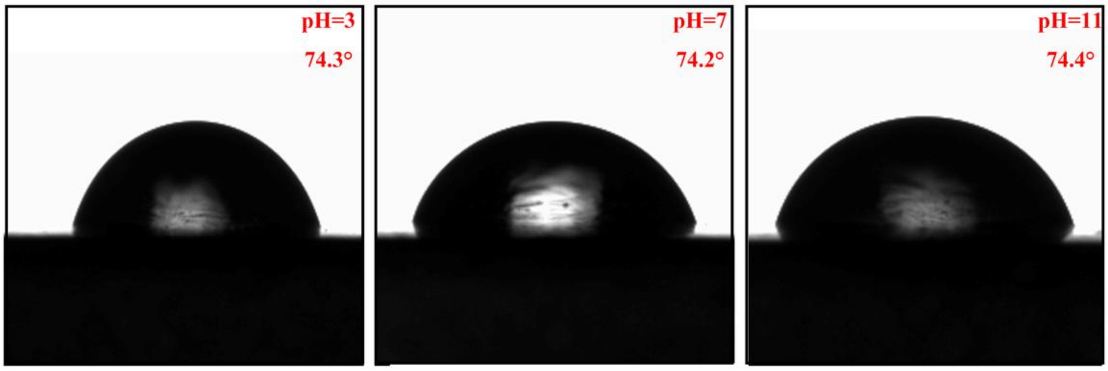

Supplementary Figure 14. The water contact angles of pristine COF membrane at different pH values, including pH=3, pH=7, and pH=11.

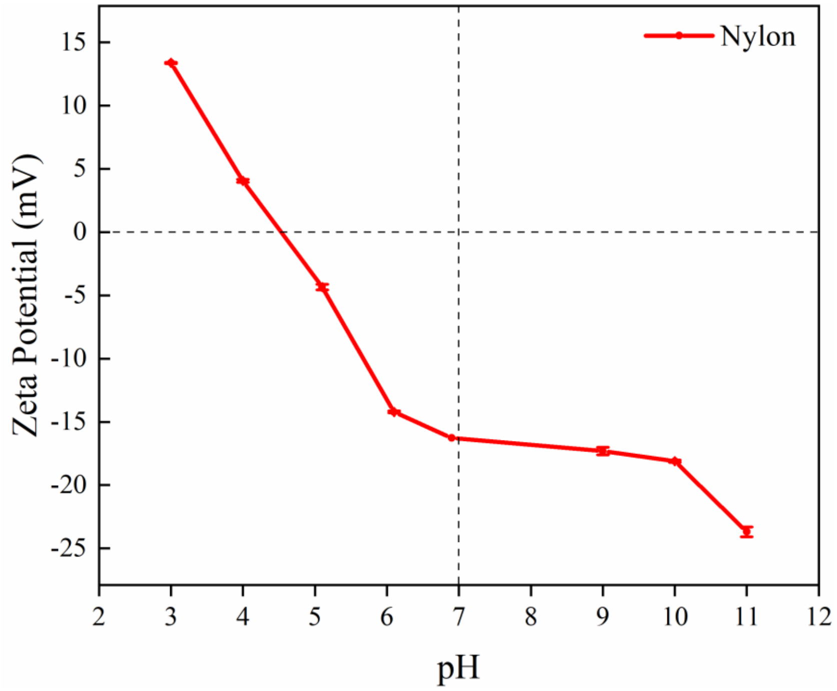

Supplementary Figure 15. Zeta potential of the Nylon substrate membrane.

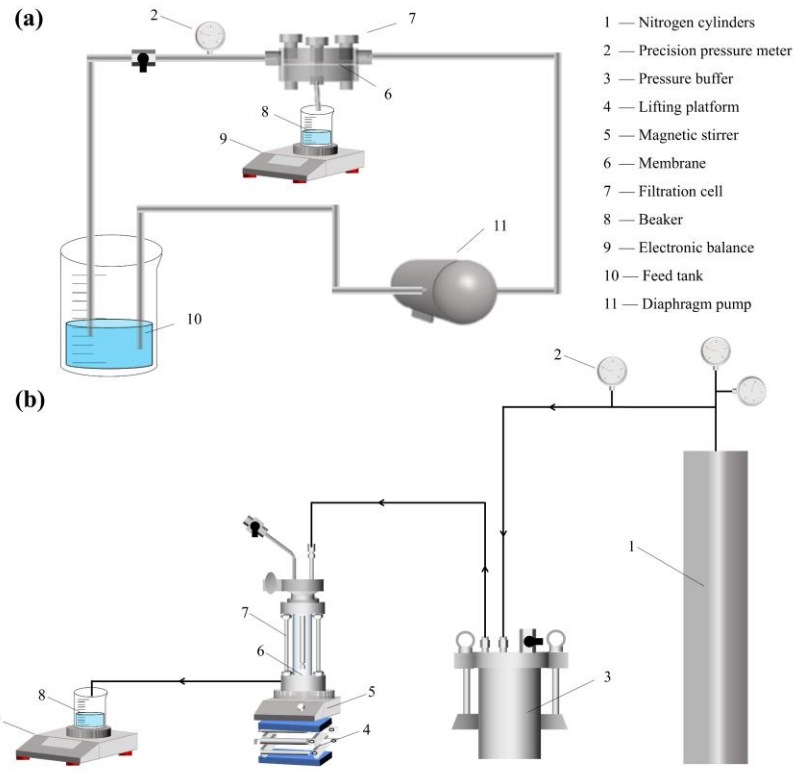

Supplementary Figure 16. Schematic illustration of the filtration cell, (a) cross-flow filtration cell and (b) dead-end filtration cell.

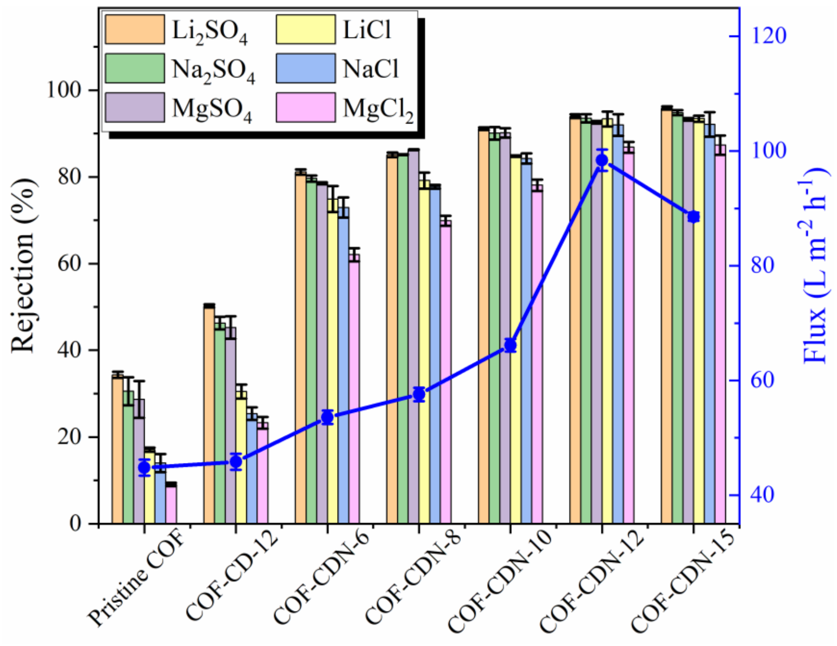

Supplementary Figure 17. The desalination performance of pristine COF membrane, COF-CD-12 membrane, COF-CDN-6 membrane, COF-CDN-8 membrane, COF-CDN-10 membrane, COF-CDN-12 membrane, and COF-CDN-15 membrane. Error bars represent the standard deviation from three independent experiments.

Note: A series of COF-CDN membranes with varying CDN contents were prepared. As the CDN content increased from 6 mL to 12 mL, the water flux (non-normalized, measured at 2.0 bar) increased from 54 to 98 L m^{-2} h^{-1}, and rejection increased to 94% for Li_2SO_4, 94% for Na_2SO_4, 93% for $MgSO_4$, 93% for LiCl, 92% for NaCl and 87% for $MgCl_2$. The observed rejection sequence displayed only a weak correlation with the fully hydrated ionic diameters, indicating that the cations undergo partial, rather than complete, dehydration within the hourglass-shaped nanochannels. Li^+ and Na^+ retained a substantial fraction of their hydration shells, and their larger hydrated diameters therefore result in the highest exclusion for Li^+, followed by Na^+. By contrast, Mg^{2+}—although it possessed the largest hydrated diameter in solution—lost a greater proportion of its hydration water in the hydrophobic constriction, acquired a smaller effective diameter, and consequently traversed the membrane more readily, resulting in the lowest rejection among the examined salts. However, further increase of CDN content led to a decline in water flux to 89 L m^{-2} h^{-1} and only a little increase of rejection. This phenomenon was likely caused by the excessive aggregation of CDN within the

nanochannels, which obstructed water flow and hindered efficient mass transport. Hence, the rejection showed a slight improvement, the overall water permeability decreased. However, the introduction of CDN initially increased the permeability of the COF-CDN membrane, reflecting the synergistic effects of the specialized nanochannel structures and enhanced surface properties, before declining due to the aggregation.

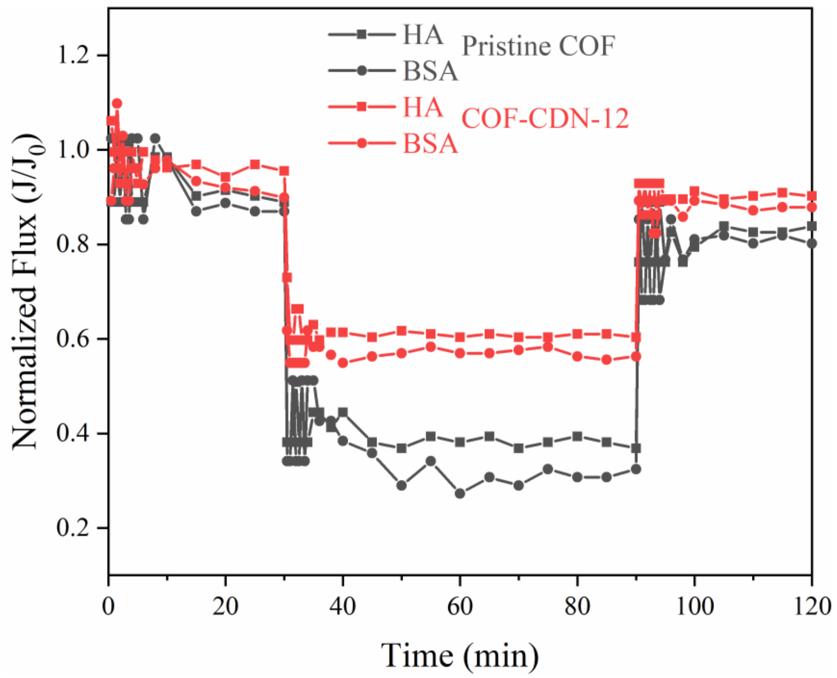

Supplementary Figure 18. Time-dependent normalized flux of the pristine COF and COF-CDN-12 membranes using different model foulant solutions.

Note: Antifouling properties of the pristine COF and COF-CDN-12 membranes were evaluated using the flux recovery rate (FRR) after filtrating model foulant solutions of humic acid (HA) or bovine serum albumin (BSA) (Supplementary **Fig. 18**). FRR of the pristine membrane was 92.7% (HA) and 92.3% (BSA), with the introduction of CDN on membrane (COF-CDN-12), the antifouling properties of the membranes increased, FRR of the COF-CDN-12 membrane was 94.1% (HA) and 95.8% (BSA). Notably, the introduction of CDN further improved the antifouling properties, which was attributed to the enhanced surface hydrophilicity and the formation of a stable hydration layer through strong hydrogen bonding between the hydrophilic groups on CDN and water molecules. This hydration layer acted as an effective antifouling barrier by preventing foulant adsorption[12], while the increased surface roughness further reinforced it by enlarging the membrane surface area and providing additional water-binding sites[13].

Supplementary Table 1. The diameter and hydrated diameter of salt ions.

Ions	Diameter (nm)	Hydrated Diameter (nm)
Lithium (Li^{+})	0.120	0.764
Sodium (Na^{+})	0.190	0.716
Magnesium (Mg^{2+})	0.130	0.856
Sulfate (SO_4^{2-})	0.580	0.758
Chloride (Cl^{-})	0.362	0.664

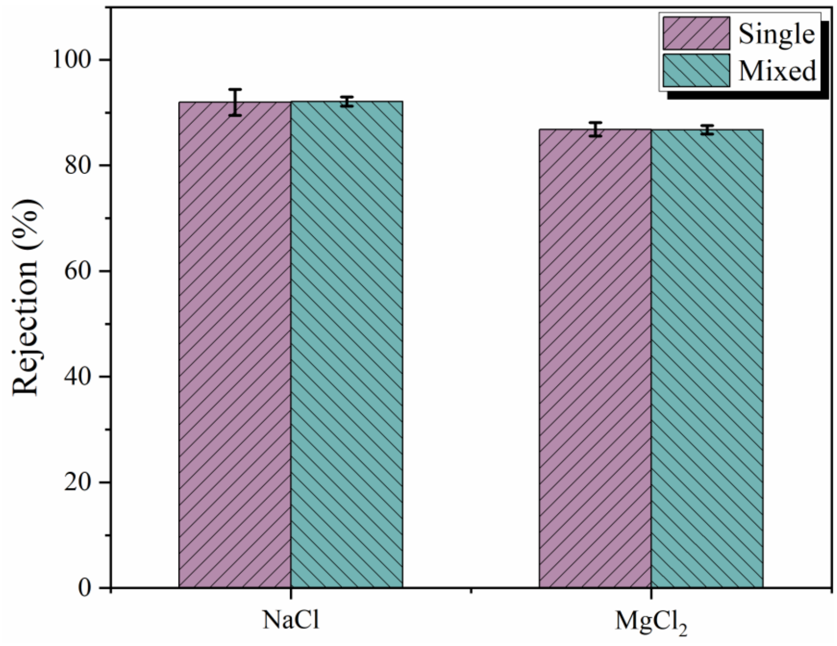

Supplementary Figure 19. Rejection of the COF-CDN-12 membrane in single and mixed salt solutions. Error bars represent the standard deviation from three independent experiments.

Note: To further simulate realistic desalination conditions, we performed separation tests using a mixed salt solution of NaCl and $MgCl_2$ (each at a concentration of 1000 ppm). The rejection obtained from the mixed salt experiments were consistent with those observed in single salt solutions, indicating the membrane effective ion rejection performance even in the mixed salt solution.

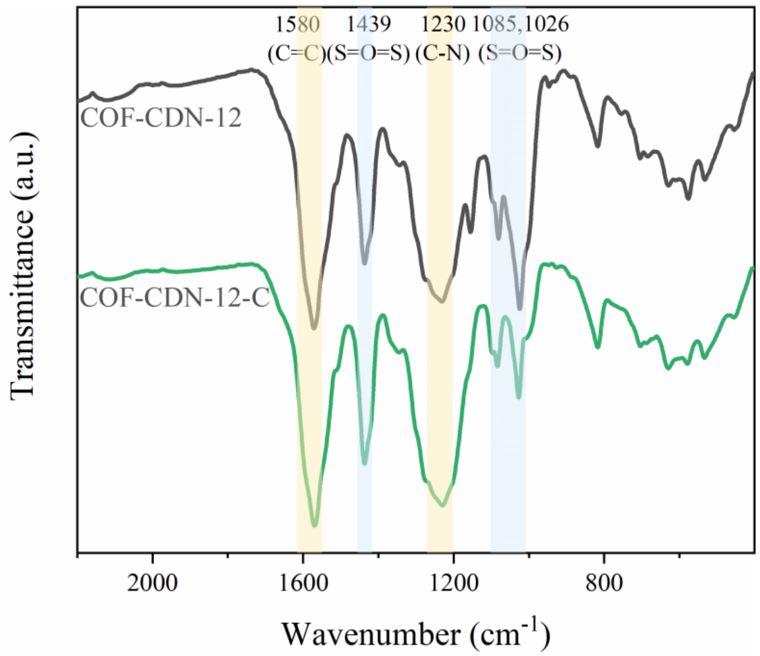

Supplementary Figure 20. FT-IR spectra of COF-CDN-12 membrane before (COF-CDN-12) and after (COF-CDN-12-C) treatment with 100 ppm sodium hypochlorite solution for 24 hours.

Note: After the treatment, FT-IR spectra indicated that the functional groups remained intact, thereby demonstrating the excellent chemical stability of membrane under oxidative conditions.

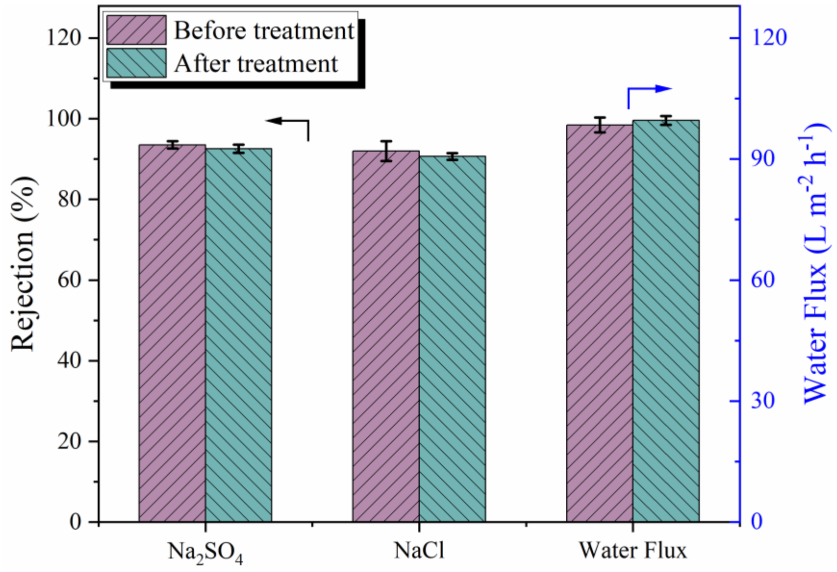

Supplementary Figure 21. Desalination performance of the COF-CDN-12 membrane before and after treatment with 100 ppm sodium hypochlorite solution for 24 hours. Error bars represent the standard deviation from three independent experiments.

Note: To further evaluate the chlorine tolerance, we conducted desalination tests after treatment with 100 ppm sodium hypochlorite solution for 24 hours. The water flux and salt rejection obtained after soaking were consistent with those measured before treatment, indicating that the membrane maintained its effective performance under chlorinated conditions.

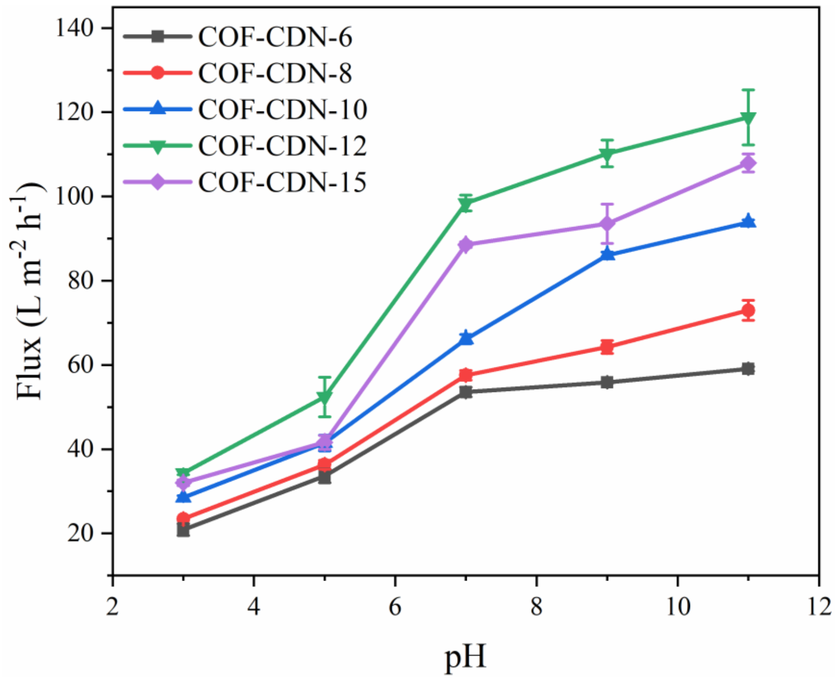

Supplementary Figure 22. Water flux (non-normalized, measured at 2.0 bar) of COF-CDN membranes at different pH values. Error bars represent the standard deviation from three independent experiments.

Note: The water flux of COF-CDN membranes was evaluated at different pH values ranging from 3 to 11. As shown in Supplementary **Fig. 22**, the water flux of all membranes increased with increasing pH of the feed solution. The water flux of the COF-CDN-12 membrane was increased from 34 L m^{-2} h^{-1} to 108 L m^{-2} h^{-1} as the pH value increased from 3 to 11. In contrast, the COF-CDN-6 membrane exhibited the lowest flux, with the water flux shifting from 21 L m^{-2} h^{-1} to 59 L m^{-2} h^{-1} as the pH value increased from 3 to 11. Membranes with higher CDN content (COF-CDN-12 membrane and COF-CDN-15 membrane) demonstrated greater water flux compared to those with lower CDN content (COF-CDN-6 membrane and COF-CDN-8 membrane), suggesting that higher CDN introduction enhanced permeability at different pH values. This behavior was due to increased hydrophilicity or changes in the membrane pore structure with increasing pH, resulting from the deprotonation of functional groups within the membrane.

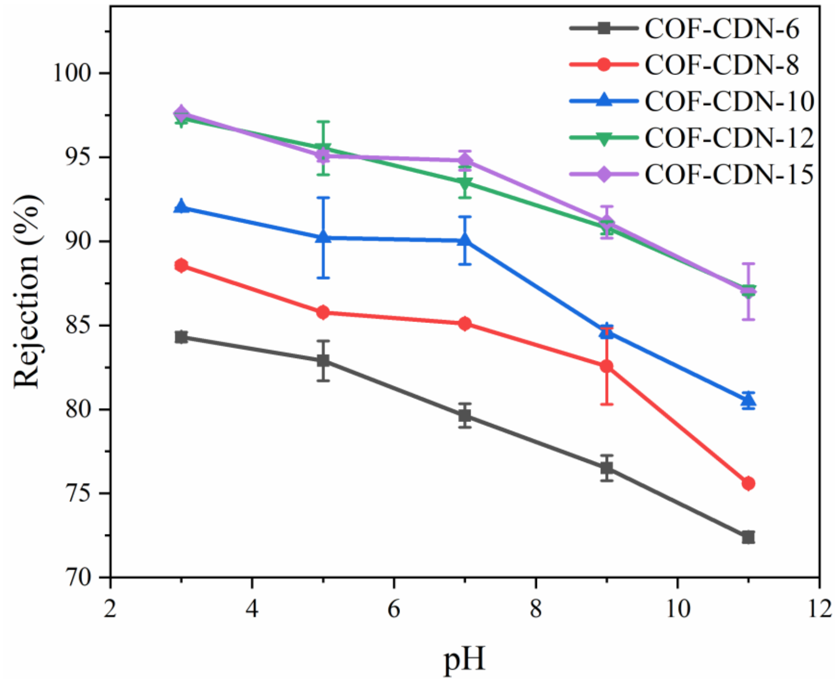

Supplementary Figure 23. Na_2SO_4 rejection of COF-CDN membranes at different pH values. Error bars represent the standard deviation from three independent experiments.

Note: The Na_2SO_4 rejection of COF-CDN membranes was evaluated at different pH values ranging from 3 to 11. As shown in Supplementary **Fig. 23**, the Na_2SO_4 rejection of all membranes decreased with increasing pH of the feed solution. The COF-CDN-15 membrane exhibited the highest rejection, decreasing slightly from 98% to 88% as the pH increased from 3 to 11. In contrast, the COF-CDN-6 membrane showed the lowest rejection, with rejection decreasing from 84% to 72% as the pH increased from 3 to 11. The observed decrease in Na_2SO_4 rejection at higher pH levels could be attributed to the deprotonation of amino groups in CDN, leading to increased effective size or reduced electrostatic interactions, thereby impacting the Na_2SO_4 rejection. Overall, these findings indicated that COF-CDN membranes with higher CDN content exhibited better Na_2SO_4 rejection under varying pH values, demonstrating their potential for efficient pH-responsive property.

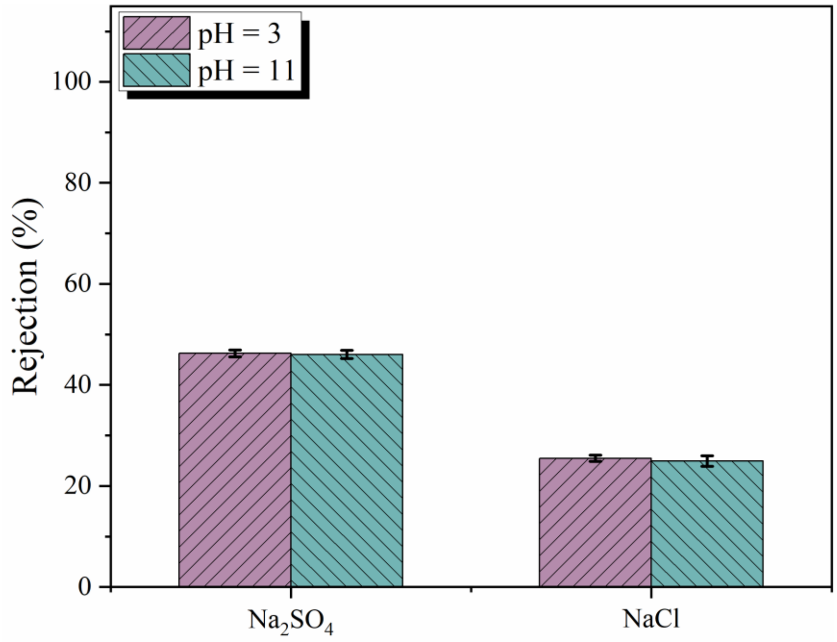

Supplementary Figure 24. Rejection of COF-CD-12 membrane at pH values of 3 and 11. Error bars represent the standard deviation from three independent experiments.

Note: For the COF-CD-12 membrane by installing CD without amino groups onto the mouth of COF membrane, the rejection of 46% for Na_2SO_4 and 25% for NaCl remained unchanged at pH values of 3 and 11, indicating that the COF-CD-12 membrane lacked pH-responsive property. These results demonstrated that the amino group plays a key role in the pH-responsive property by dynamically regulating of the effective size and charge.

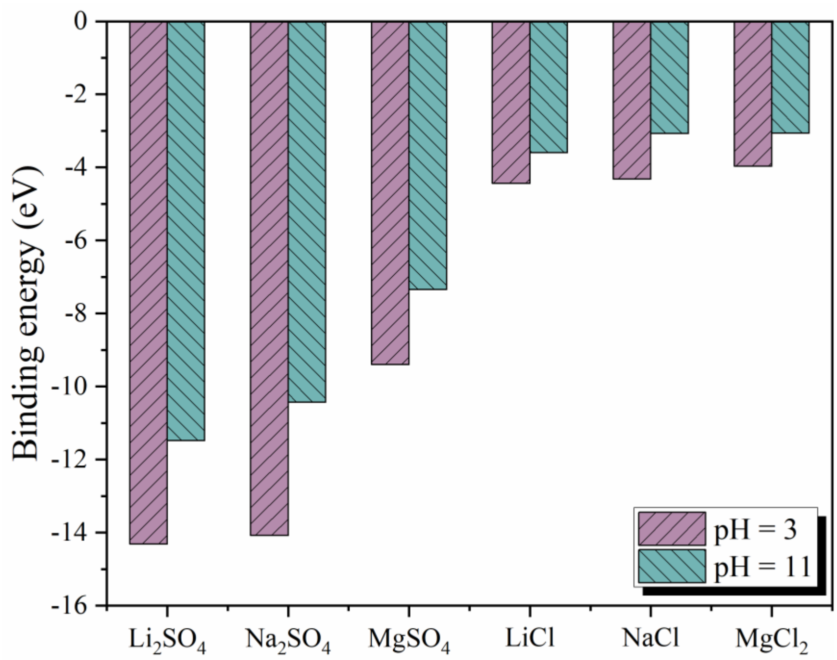

Supplementary Figure 25. Binding energy between salt and CDN at different pH values calculated by DFT.

Note: Meanwhile, DFT calculations were conducted considering both cation and anion interactions under protonated and deprotonated states. The overall binding energy sequence (Li_2SO_4 > Na_2SO_4 > $MgSO_4$ > LiCl > NaCl > $MgCl_2$) was calculated, reflecting the combined contributions of anion and cation affinities toward the membrane. These results indicated that ion rejection was controlled by interactions involving both cations and anions, rather than only by Donnan exclusion. This behavior was attributed to the fact that, after protonation ($-NH_2$ to $-NH_3^+$), the membrane surface became positively charged, which not only repelled cations but also attracted anions, particularly divalent anions such as SO_4^{2-}. The electrostatic attraction of anions could lead to the formation of a negatively charged anion concentration layer at the membrane surface, which might subsequently enhance the rejection of additional anions from the solution.

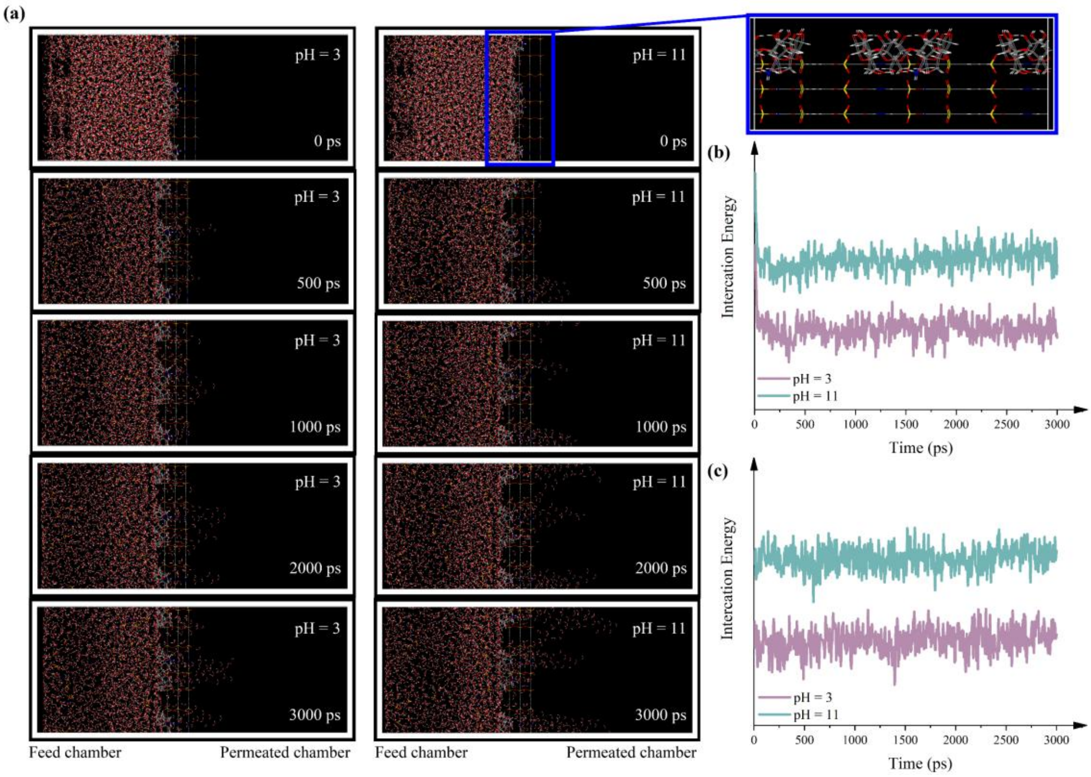

Supplementary Figure 26. (a) Simulation snapshots of Na_2SO_4 solution transporting through the hourglass-shaped nanochannels at 0 ps, 500 ps, 1000 ps, 2000 ps and 3000 ps. Interaction energy between the membranes and (b) water or (c) Na_2SO_4 solution.

Note: To validate these transport characteristics, all-atom MD simulations were performed to explicitly track the movement of water molecules and ions across the COF-CDN membrane separating the feed and permeate reservoirs. The results showed that, under neutral or alkaline conditions (i.e., with deprotonated hourglass-shaped nanochannels), water molecules were able to rapidly diffuse through the hourglass-shaped nanochannels, with a large number of molecules traversing from the feed side to the permeate side within a short simulation period. In contrast, under acidic conditions (i.e., with protonated hourglass-shaped nanochannels), only a few water molecules were observed to pass through the channel during the same timeframe (Supplementary **Fig. 26**a), consistent with the experimentally observed low water flux and high salt rejection under acidic conditions.

Moreover, no salt ions were observed to permeate the membrane within the simulation timescale under either condition, which was consistent with the experimentally observed high salt rejection. The interactions between functional groups

and water/salt molecules as they passed through the hourglass-shaped nanochannels formed by COF-CDN were analyzed (Supplementary **Fig. 26**b-c). The results revealed that both water and Na_2SO_4 exhibited stronger interaction energy (i.e., more negative values) with the protonated hourglass-shaped nanochannels. These stronger interactions hindered the passage of water molecules and salt ions through the hourglass-shaped nanochannels, thereby contributing to the experimentally observed lower water flux and higher salt rejection under acidic conditions.

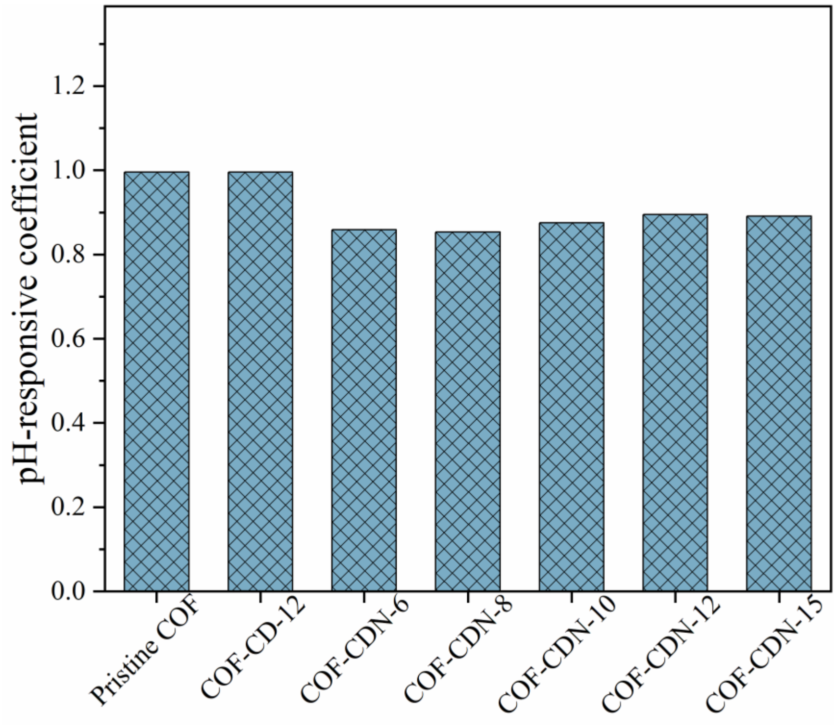

Supplementary Figure 27. pH-responsive coefficient for Na_2SO_4 rejection.

Note: The pH-responsive coefficient was defined as the ratio of the Na_2SO_4 rejection at a pH value of 11 to that at a pH value of 3[14,15]. The pH-responsive coefficient for Na_2SO_4 rejection of the pristine COF membrane and COF-CD-12 membrane was close to 1, indicating that the Na_2SO_4 rejection was largely independent of pH values. However, after the introduction of CDN, the pH-responsive coefficient for Na_2SO_4 rejection decreased, indicating that the rejection became dependent on pH values. After protonation (-NH_2 to -NH_3^+), the membrane surface became positively charged, which not only rejected cations but also attracted anions. This attraction could lead to the formation of a negatively charged anion concentration layer at the membrane surface, which could subsequently enhance the rejection of additional anions from the solution. In addition, under acidic conditions, the amino groups in CDN were protonated, causing the CDN cavity to shrink, with the hydrophilic conical entrance size of 16.1 Å and the hydrophobic spot size of 4.7 Å. Consequently, the pore of COF-CDN membranes either decreased in effective size or enter a "closed" state. This combination of effects led to reduced water flux and increased rejection at lower pH values. In contrast, under neutral or alkaline conditions, the amino groups in CDN were deprotonated, leading to a reduced surface charge and an extension of the CDN cavity. Consequently, the pores of

COF-CDN membranes entered an “open” state, with the hydrophilic conical entrance size of 16.7 Å and the hydrophobic spot size of 5.3 Å, exposing more space for water and salt permeation and leading to increased water flux and reduced rejection at higher pH values.

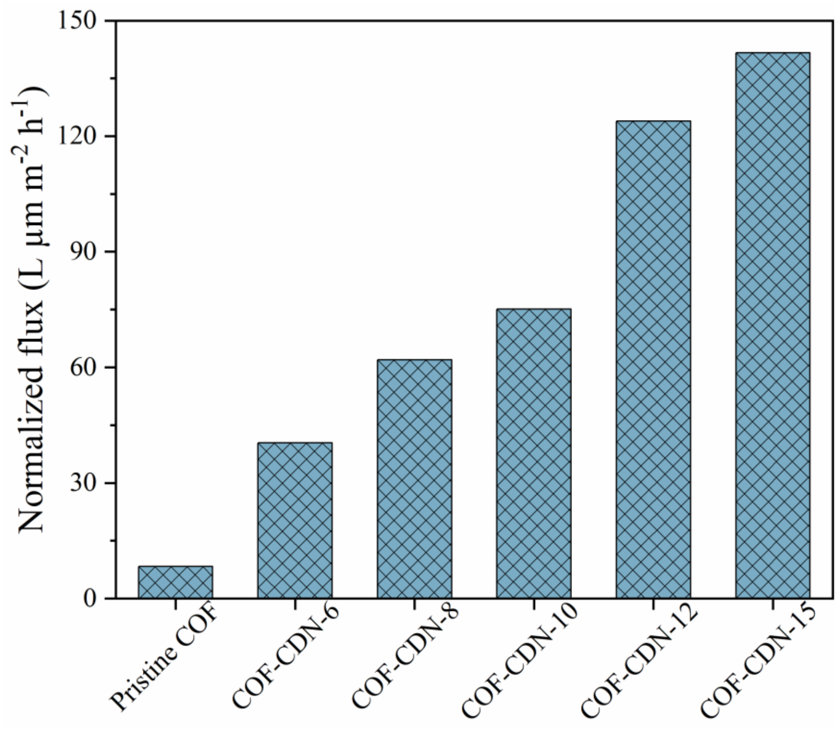

Supplementary Figure 28. Thickness-normalized water flux of different membranes.

Note: The flux was normalized by multiplying it by the membrane thickness to eliminate the effects of thickness variations. With the increase of CDN content, the formation of hourglass-shaped nanochannels was enhanced, accompanied by improved membrane hydrophilicity. These hourglass-shaped nanochannels effectively reduced the mass transport resistance, thereby facilitating fast and low-resistance water transport, as evidenced by monotonically increasing thickness-normalized flux. When an excessive amount of CDN was introduced (COF-CDN-15), dense interfacial assembly was likely to cause partial misalignment of the hourglass-shaped nanochannels. The misalignment of the nanochannels disrupted the continuity of the water transport pathways, leading to larger transport resistance and lower membrane separation performance, as reflected by the initial increase and subsequent decrease in the water flux (Supplementary **Fig. 17**).

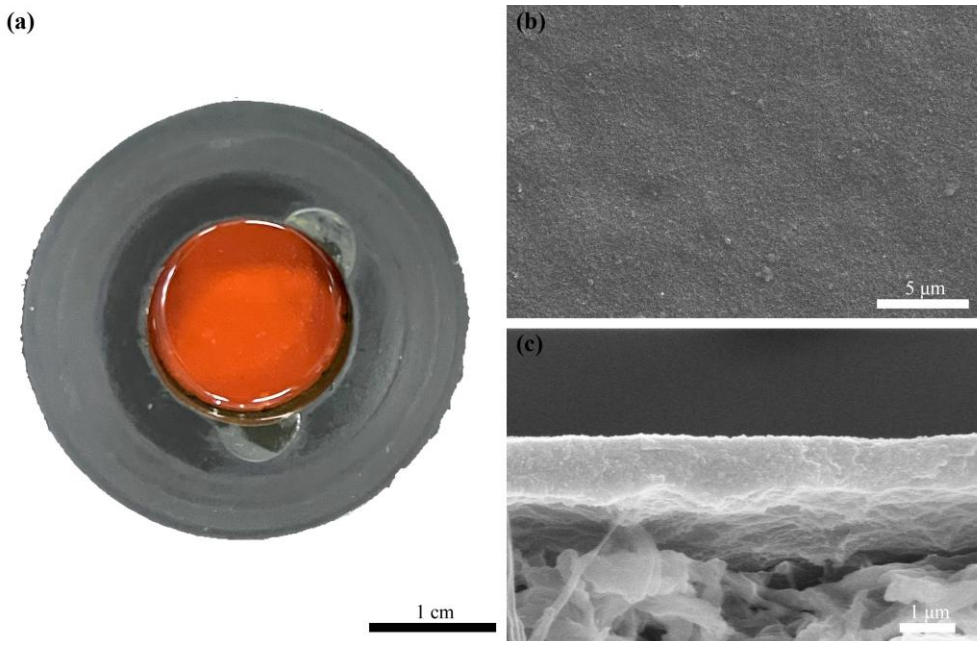

Supplementary Figure 29. (a) Digital photo, (b) surface and (c) cross-sectional SEM images of COF-CDN-12 membrane after long-term testing.

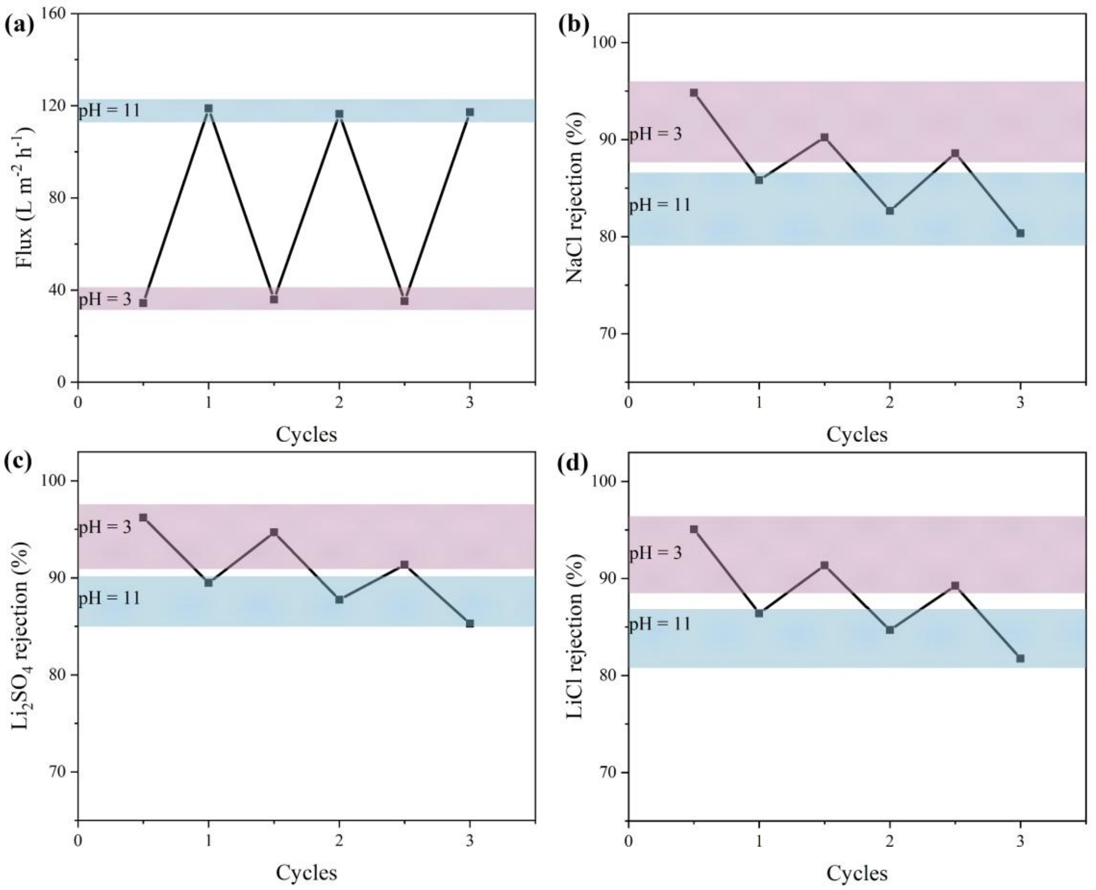

Supplementary Figure 30. (a) Flux (non-normalized, measured at 2.0 bar), (b) NaCl, (c) Li_2SO_4 and (d) LiCl rejection of COF-CDN-12 membrane when the pH shifted from 3 to 11 after 3 cycles.

Note: As shown in Supplementary **Fig. 30**a, the COF-CDN-12 membrane maintained consistent water flux, with no significant decrease in performance observed at either pH value of 3 or 11. As shown in **Fig. 6**b and Supplementary **Fig. 30**b-d, a slight decline in rejection was observed at pH 3 or pH 11 during the cycling test, in which the pH value was shifted between 3 to 11. This was attributed to partial retention of salt ions within the membrane pores. Specifically, when the pH shifted from 3 to 11, the pores of COF-CDN membranes entered an “open” state, allowing salt ions to pass through more easily. Some of these salt ions were retained within the membrane pores. When the pH returned from 11 to 3, these residual salt ions interfered with the complete re-protonation of COF-CDN membranes, resulting in a slight decline in rejection during the subsequent cycle.

Supplementary Table 2. Separation performance of the COF-CDN membrane compared with other membranes reported in the literature.

Membrane	Water permeance ($L\ m^{-2}\ h^{-1}\ bar^{-1}$)	Rejection (%)		Ref.
		Na_2SO_4	NaCl	
LiOH-Am7CD-TMC	2.0	99.5	55.2	16
CNT-TFNi	18.9	98.0	17.9	17
NT-OEt/PIP TMC	41.7	98.1	11.0	17
pDA/TpPa(W/E)	51.3	99.5	49.2	18
COF-LZU1	44.2	63.6	-	19
PaTp-TMC	0.8	96.6	93.3	20
PA/PDA-COF	16.4	97.2	14.6	21
FS-COM-1	3.9	90.0	-	22
TpHz/PES	4.1	58.3	6.7	23
$TpTG_{Cl}$@CNFs	42.8	96.8	25.0	24
IISERP-COOH-COF1	0.5	96.3	82.9	25
COF nanofibers	31.1	95.0	16.0	26
PA-SNW-1/PES	19.3	83.5	18.0	27
COF-CDN	49.2	93.5	91.9	This work

References

1 Carpenter, J. E. Extension of Lewis structure concepts to open-shell and excited-state molecular species. *University of Wisconsin--Madison* (1987).

2 B. Hess, C. Kutzner, D. van der Spoel & E. Lindahl. GROMACS 4: Algorithms for Highly Efficient, Load-Balanced, and Scalable Molecular Simulation. *J. Chem. Theory Comput.* **4**, 435–447 (2008).

3 J. Wang, R. M. Wolf, J. W. Caldwell, P. A. Kollman & D. A. Case. Development and testing of a general amber force field. *J. Comput. Chem.* **25**, 1157–1174 (2004).

4 C. I. Bayly, P. Cieplak, W. Cornell & P. A. Kollman. A Well-Behaved Electrostatic Potential Based Method Using Charge Restraints for Deriving Atomic Charges: The RESP Model. *J. Phys. Chem.* **97**, 10269–10280 (1993).

5 Berendsen, H. J. C., Postma, J. P. M., van Gunsteren, W. F., DiNola, A. & Haak, J. R. Molecular dynamics with coupling to an external bath. *The Journal of Chemical Physics* **81**, 3684-3690 (1984).

6 Abascal, J. L. & Vega, C. A general purpose model for the condensed phases of water: TIP4P/2005. *J Chem Phys* **123**, 234505 (2005).

7 Astrakas, L. G., Gousias, C. & Tzaphlidou, M. Structural destabilization of chignolin under the influence of oscillating electric fields. *Journal of Applied Physics* **111** (2012).

8 Hess, B., Bekker, H., Berendsen, H. J. & Fraaije, J. G. LINCS: A Linear Constraint Solver for Molecular Simulations. *J. Comput. Chem.* **18**, 1463-1472 (1997).

9 Van Gunsteren, W. F. & Berendsen, H. J. C. A Leap-frog Algorithm for Stochastic Dynamics. *Molecular Simulation* **1**, 173-185 (2007).

10 Chandra, S. *et al.* Interplaying intrinsic and extrinsic proton conductivities in covalent organic frameworks. *Chemistry of Materials* **28**, 1489-1494 (2016).

11 Peng, Y. *et al.* Mechanoassisted synthesis of sulfonated covalent organic frameworks with high intrinsic proton conductivity. *ACS Applied Materials & Interfaces* **8**, 18505-18512 (2016).

12 Huang, H. *et al.* Strong Surface Hydration and Salt Resistant Mechanism of a New Nonfouling Zwitterionic Polymer Based on Protein Stabilizer TMAO. *J Am Chem Soc* **143**, 16786-16795 (2021).

13 Wang, Y. *et al.* Thermo-adaptive interfacial solar evaporation enhanced by dynamic water gating. *Nat Commun* **15**, 6157 (2024).

14 Zhao, G. & Zhu, H. Self‐regulating cross‐linked graphene oxide membranes with stable retention properties over a wide pH range. *Advanced Materials Interfaces* **7**, 1901535 (2019).

15 Guo, Q. *et al.* pH-responsive nanofiltration membrane containing chitosan for dye separation. *J. Membr. Sci.* **635**, 119445 (2021).

16 Hong, S. *et al.* Precision ion separation via self-assembled channels. *Nature Communications* **15**, 3160 (2024).

17 Han, S. *et al.* Microporous organic nanotube assisted design of high performance nanofiltration membranes. *Nature Communications* **13**, 7954 (2022).

18 Zhang, Y. *et al.* Molecularly soldered covalent organic frameworks for ultrafast precision sieving. *Science Advances*, eabe8706 (2021).

19 Yin, C., Fang, S., Shi, X., Zhang, Z. & Wang, Y. Pressure-modulated synthesis of self-repairing covalent organic frameworks (COFs) for high-flux nanofiltration. *J. Membr. Sci.* **618**, 118727 (2021).

20 Kong, F.-x., Yue, L., Yang, Z., Sun, G. & Chen, J.-f. Cross-Linked Covalent Organic Framework-Based Membranes with Trimesoyl Chloride for Enhanced Desalination. *ACS Applied Materials & Interfaces* **13**, 21379-21389 (2021).

21 Wu, M. *et al.* Ultrathin nanofiltration membrane with polydopamine-covalent organic framework interlayer for enhanced permeability and structural stability. *J. Membr. Sci.* **576**, 131-141 (2019).

22 Li, Y. *et al.* Laminated self-standing covalent organic framework membrane with uniformly distributed subnanopores for ionic and molecular sieving. *Natrue Communications* **11**, 599 (2020).

23 Wang, R., Wei, M. & Wang, Y. Secondary growth of covalent organic

frameworks (COFs) on porous substrates for fast desalination. *J. Membr. Sci.* **604**, 118090 (2020).

24 Yang, H. *et al.* Covalent organic framework membranes through a mixed-dimensional assembly for molecular separations. *Natrue Communications* **10**, 2101 (2019).

25 Liu, C., Jiang, Y., Nalaparaju, A., Jiang, J. & Huang, A. Post-synthesis of a covalent organic framework nanofiltration membrane for highly efficient water treatment. *Journal of Materials Chemistry A* **7**, 24205-24210 (2019).

26 Zhang, Z., Shi, X., Wang, R., Xiao, A. & Wang, Y. Ultra-permeable polyamide membranes harvested by covalent organic framework nanofiber scaffolds: a two-in-one strategy. *Chemical Science* **10**, 9077-9083 (2019).

27 Wang, C. *et al.* Covalent organic framework modified polyamide nanofiltration membrane with enhanced performance for desalination. *J. Membr. Sci.* **523**, 273-281 (2017).
