# Supplementary material for: Covalent organic framework membrane with hourglass-shaped nanochannels for ultrafast desalination
Source: Nat Commun. 2025 Aug 30;16:8125. doi: 10.1038/s41467-025-63650-5 (PMC12398615; doi:10.1038/s41467-025-63650-5)
Supplement: Supplementary file 2 — Transparent Peer Review file [file 41467_2025_63650_MOESM2_ESM.pdf]

nature portfolio

Peer Review File

Covalent organic framework membrane with hourglass-shaped nanochannels for ultrafast desalination

Corresponding Author: Professor Zhongyi Jiang

This file contains all reviewer reports in order by version, followed by all author rebuttals in order by version.

Version 0:

Reviewer comments:

Reviewer #1

(Remarks to the Author)
This study reports the fabrication of a covalent organic framework (COF) membrane with hourglass-shaped nanochannels by incorporating amino-cyclodextrin nanoparticles (CDN) at the nanochannel entrances. By engineering a hydrophilic conical entrance and a hydrophobic spout, the membrane demonstrated both high pure water flux and salt rejection rate exceeding 90% across various salt solutions. The authors investigated the role of pH-responsive amino groups in the CDN component in regulating the effective size and charge state of the nanochannels to enhance water flux and ion rejection. While this work is interesting and provides valuable insights into the design of COF channels, the experimental design, material characterization, and modelling work do not provide sufficient information about the water and ion transport mechanisms. Furthermore, key aspects such as scalability and long-term performance stability under realistic membrane desalination conditions (high pressure, complex feed compositions) remain unaddressed. Nevertheless, apart from the enhanced desalination performance, another notable strength of this work is the potential chlorine resistance of the proposed membrane design, which could offer significant advantages over conventional polyamide-based membranes.

1. The method used to achieve the specific structure with well-aligned CDN onto the COF membranes was not clearly explained. Is there direct evidence or characterization data to confirm the alignment and incorporation of CDN into the membrane?
2. The reported increase in membrane thickness by approximately 10 times (from 0.19 to 1.26 microns) raises concerns about the potential increase in resistance. How does this significant change in thickness affect water flux and salt rejection? Specifically, how do the improvements in hydrophilicity and the hourglass-shaped channels with reduced channel size counterbalance the increased resistance caused by the thicker membrane?
3. Following the above, the modelling of the channel size in the two states of COF-CDN membranes does not sufficiently explain the observed enhancements in water flux and salt rejection compared to the COF membrane alone. To provide stronger support for the desalination performance, it would be more informative to include a detailed analysis of the interactions between functional groups and water/salt molecules as they pass through the hourglass channels formed by COF-CDN with varying properties across the channels. Additionally, molecular dynamics (MD) simulations of water and salt transport across these membranes over time could offer more direct insights. The change in thickness between COF membranes and COF-CDN membranes should be factored in. Current modelling of the change in pore size of amino-cyclodextrin at different pH, which is known and previously reported, adds limited value.
4. Generally, the increase in the roughness and amino groups on the surface could lead to more significant fouling, while increased hydrophilicity would reduce the surface interaction with the foulants. It is suggested that fouling experiments should be performed to determine these effects.
5. The capillary effect induced by the differences in hydrophilicity in Line 197 is unclear. Is this effect supported experimentally or consistent with the findings from the literature?
6. The FT-IR analysis, water contact angle, and zeta potential measurements conducted using different sets of samples with varying CDN loadings. In FT-IR, the CDN loading is 4, 7, 10, 13, 16, while it is 8, 10, 12, 15 for water contact angle, and 6, 12, 15 for zeta potential. The same range of samples should be used to aid comparison. Additionally, please check if "COF-CD" in Fig 3c should read "COF-CDN"
7. It is obvious that the salt rejection decreases at each cycle in the crossflow filtration test. The authors should revise line 348 to reflect this.
8. The membrane desalination performance in realistic conditions (relevant pressure, mixed salt solutions) should be evaluated.

9. Line 295-300: The explanation of ion transmembrane behavior using the Donnan effect in this section may not be entirely accurate. After pH adjustment, the functional groups on the membrane surface change from $-NH_2$ to $-NH_3^+$, resulting in a positively charged membrane surface. This surface charge should affect both anions and cations: for cations, higher-valent cations such as Mg^{2+} would experience greater electrostatic repulsion; for anions, higher-valent anions such as SO_4^{2-} would exhibit stronger attraction. Due to the strong electrostatic attraction of divalent anions by the membrane, compared to monovalent anions, a stronger negatively charged anion concentration layer may form on the membrane surface, which would further repel anions in the solution. To achieve a more comprehensive interpretation, both cation and anion interactions should be considered simultaneously.
10. Line 236-247: The discussion of ion rejection in this section with the related Supporting Information Figure 17 requires further verification. According to the membrane pore size and hydrated ion diameters provided in the manuscript, ions should permeate the membrane through a dehydration mechanism. Based on this mechanism, sodium ions (Na^+) should exhibit the highest rejection rate due to their dehydrated diameter, followed by magnesium ions (Mg^{2+}), and lithium ions (Li^+). However, the data presented in the article indicate the highest rejection rate for Li^+. If hydrated diameters alone governed rejection rates, Mg^{2+} (with the largest hydrated size) should show the highest rejection, followed by Li^+ and then Na^+ (smallest hydrated size). This discrepancy may arise from unaccounted factors, such as ion dehydration energy due to varying surface properties across the ion channels.
11. Line 319-322: The discussion provided regarding the decreased pure water flux of the COF-CDN-15 membrane, attributed primarily to increased membrane thickness, may overlook additional factors. Previous data in the article show significant variations in membrane thickness. To isolate thickness effects, we recommend normalizing the flux by membrane thickness to compare the material performance under equivalent thickness conditions. A quick calculation based on the results in the manuscript reveal that the water flux of thickness-corrected COF-CDN-15 membranes remains lower than that of other membranes. While the data confirms COF-CDN-15's superior hydrophilicity compared to other membranes, this enhancement alone cannot fully account for the observed flux reduction. It may be worth exploring whether other factors, such as decreases in membrane pore size or increased thickness (both adding hydraulic resistance), contribute to this phenomenon.
12. Overall, to provide a more accurate interpretation of the excellent desalination performance, computational simulation on the changes to the energy barrier and ion transport rate induced by CDN along with recently reported electrochemical characterization techniques for molecule/ion transport may provide more useful information.

Other comments:
13. Please check Supplementary Figure 22 as both b and c are figures for LiCl rejection.
14. Please check and confirm whether the labels (Tp and PaSO3H) in Supplementary Figure 1 correspond to their respective FT-IR spectra.
15. In Supplementary Figure 13, the data for COF-CDN-6-A and COF-CDN-6 in Figure (a), as well as COF-CDN-6 and COF-CDN-6-A in Figure (c), appear to be very similar.
16. It was not clear how the wettability test at different pH levels was performed.
17. The details of the crossflow velocity and membrane area used for crossflow filtration experiments are missing and should be provided.
18. The water flux data corresponding to Fig. 6b should be provided.
19. Could you authors comment on the scalability of COF-CDN membrane fabrication process?
20. The overall quality of the manuscript's writing needs improvement to ensure clarity and readability.

Reviewer #2

(Remarks to the Author)
[Editor Note: The following review was listed as "Remarks to Editor"]

The manuscript by Wei et al. presents a well-executed study on the development of a covalent organic framework (COF) membrane with hourglass-shaped hetero-nanochannels for high-performance desalination. By incorporating amino-cyclodextrin nanoparticles (CDN) onto COF membranes through sequential assembly, the authors successfully engineer nanochannels featuring a hydrophilic conical entrance and a hydrophobic spout, which enhance water transport while maintaining effective ion rejection. The introduction of pH-responsive functionality further enables dynamic regulation of channel size and charge, optimizing membrane performance. Moreover, the membrane demonstrates excellent stability over a seven-day operational period. The authors should be commended for their thorough characterization of the membrane.

However, several minor revisions are required to improve the manuscript:

Figure Quality: Several figures, including Figures 1, 2, and 5, are of low resolution and appear blurry when zoomed in for details. Additionally, Figure 3(a) and Supplementary Information (SI) Figure 7 are overly light, making it difficult to discern membrane morphology. Higher-quality images should be provided to enhance clarity.

Technical Clarification (Lines 296–299): The claim that the positively charged membrane repels anions requires further clarification, as this is not consistent with expected electrostatic interactions. More insight is needed to explain why the membrane exhibits a higher rejection rate in acidic environments.

Data Interpretation and Characterization (Lines 350–354, Figure 6(b), and SI Figure 22): The manuscript states that rejection shows "no significant decrease," whereas the data clearly indicate a decline. A 1% difference is substantial in the context of desalination membranes, where even small variations can impact performance. This discrepancy should be corrected, and

further explanation is required regarding the observed decline in rejection rate during pH shift cycles. Additional membrane characterization would be beneficial in elucidating this behavior.

Reviewer #3

(Remarks to the Author)
[Editor Note: See Attached]

I co-reviewed this manuscript with one of the reviewers who provided the listed reports. This is part of the Nature Communications initiative to facilitate training in peer review and to provide appropriate recognition for Early Career Researchers who co-review manuscripts.

Reviewer #4

(Remarks to the Author)
In this work, the authors designed and constructed hetero-nanochannels by incorporating amino-cyclodextrin nanoparticles (CDN) into the mouth of COF membrane to pursue ultrafast desalination. The resulting nanochannels consist of a wide hydrophilic conical entrance and a narrow hydrophobic spout onto cylindrical nanochannels. The COF membrane with such hetero-nanochannels achieves high desalination performance and significantly breaks the trade-off between membrane permeability and selectivity. This work is quite innovative and interesting, stimulating the thinking about the design of advanced membrane structures. This manuscript is well organized, and well-written. Some minor issues should be addressed before the manuscript is accepted.

1. The membrane name (COF-CD-4, COF-CD-7...) in Fig. 3c is different from that (COF-CDN-4, COF-CDN-7...) used elsewhere in this manuscript, which should be unified and corrected.

2. The quality of Fig. 4 needs to be improved, as the color and pattern appear pixelated or blurry in the provided image.

3. The experimental details for the activation (degassing) of the COF nanosheets in the nitrogen adsorption-desorption experiment should be added.

4. A slight deviation is observed between the simulated and experimental XRD patterns of the COF powder, particularly in the (100) peak position. The explanations should be provided.

5. As a comparison, the rejection data of COF-CD-12 should be offered, and explanations about the effect of the amino group on the rejection should be provided.

Version 1:

Reviewer comments:

Reviewer #1

(Remarks to the Author)
The authors have addressed all concerns raised by the reviewers satisfactorily. I recommend that the paper be accepted for publication.

Reviewer #2

(Remarks to the Author)
In this manuscript, the authors the synthesize and characterize a COF-based membranes and test it for water flux and salt rejection. There are a few issues with this manuscript:

1) The salt rejection values are not great, and correspond to a NF-type membrane. It is not clear what such a membrane is useful for. It is too low for seawater (and was not evaluated under seawater conditions), and low salt rejecting membranes are abundantly available from many commercial vendors. Not clear where such a membrane fits into the existing landscape, nor what is the point of using it (assuming it can be manufactured at scale, which is not explored here).

2) The flux data is unclear. At one point, the authors state that they normalize the flux by thickness. However, in other figures (e.g., Figure 4) the flux is listed as not normalized, although the values are very similar to the normalized values. Which is it? Also, flux should be expressed as permeability (e.g., LMH/bar) in these kinds of studies.

3) There is no mention of chlorine tolerance. This is an important point, and really the only reason such a material may be useful (assuming it has some tolerance). This was called out by a reviewer but was not addressed, suggesting there is an issue?

4) The data in Figure 6c is not clear. It is not reasonable to compare NaCl and Na2SO4 rejection - the latter will always have

far higher rejection.

I guess I just don't "get" what the point of this paper is.

Reviewer #3

(Remarks to the Author)
I co-reviewed this manuscript with one of the reviewers who provided the listed reports. This is part of the Nature Communications initiative to facilitate training in peer review and to provide appropriate recognition for Early Career Researchers who co-review manuscripts.

Reviewer #4

(Remarks to the Author)
The authors have adequately addressed my previous concerns. I believe the paper is now in a suitable form for publication.

Version 2:

Reviewer comments:

Reviewer #2

(Remarks to the Author)
The authors have addressed my concerns.

Reviewer #3

(Remarks to the Author)
I co-reviewed this manuscript with one of the reviewers who provided the listed reports. This is part of the Nature Communications initiative to facilitate training in peer review and to provide appropriate recognition for Early Career Researchers who co-review manuscripts.

Open Access This Peer Review File is licensed under a Creative Commons Attribution 4.0 International License, which permits use, sharing, adaptation, distribution and reproduction in any medium or format, as long as you give appropriate credit to the original author(s) and the source, provide a link to the Creative Commons license, and indicate if changes were made.
In cases where reviewers are anonymous, credit should be given to 'Anonymous Referee' and the source.
The images or other third party material in this Peer Review File are included in the article's Creative Commons license, unless indicated otherwise in a credit line to the material. If material is not included in the article's Creative Commons license and your intended use is not permitted by statutory regulation or exceeds the permitted use, you will need to obtain

permission directly from the copyright holder.
To view a copy of this license, visit https://creativecommons.org/licenses/by/4.0/

Response to comments

Reviewer #1: This study reports the fabrication of a covalent organic framework (COF) membrane with hourglass-shaped nanochannels by incorporating amino-cyclodextrin nanoparticles (CDN) at the nanochannel entrances. By engineering a hydrophilic conical entrance and a hydrophobic spout, the membrane demonstrated both high pure water flux and salt rejection rate exceeding 90% across various salt solutions. The authors investigated the role of pH-responsive amino groups in the CDN component in regulating the effective size and charge state of the nanochannels to enhance water flux and ion rejection. While this work is interesting and provides valuable insights into the design of COF channels, the experimental design, material characterization, and modelling work do not provide sufficient information about the water and ion transport mechanisms. Furthermore, key aspects such as scalability and long-term performance stability under realistic membrane desalination conditions (high pressure, complex feed compositions) remain unaddressed. Nevertheless, apart from the enhanced desalination performance, another notable strength of this work is the potential chlorine resistance of the proposed membrane design, which could offer significant advantages over conventional polyamide-based membranes.

Response: We would like to thank the reviewer for the highly positive remarks and valuable guidance. We have carefully revised the manuscript and the supplementary information, and have addressed each point in detail. We hope that this version of the manuscript and the supplementary information can enhance the clarity and scientific depth of our study.

Comment R1-1: The method used to achieve the specific structure with well-aligned CDN onto the COF membranes was not clearly explained. Is there direct evidence or characterization data to confirm the alignment and incorporation of CDN into the membrane?

Response R1-1: Thank the reviewer for the valuable guidance. In this study, we fabricated the COF membranes with hourglass-shaped nanochannels by assembling

COF nanosheets onto a Nylon substrate membrane, followed by the installing of CDN onto the mouth of COF membrane. This assembly method enables the anchoring of CDN at the mouth of the COF, resulting in geometry reminiscent of an hourglass. The successful alignment of CDN on the COF relies on hydrogen bonding and electrostatic interactions between amino (–NH_2) or hydroxyl (–OH) groups in CDN and the sulfonic acid (–SO_3H) in the COF (*Angew. Chem. Int. Ed.*, *2025, 64, e202415454*, *Chem. Soc. Rev., 2024, 53, 2738–2760*). These interactions not only promote the stabilization of CDN onto the mouth of COF membrane (*J. Am. Chem. Soc., 2020, 142(30), 12987–12994*) but also facilitate a relatively ordered arrangement (*J. Am. Chem. Soc., 2006, 128, 12307–12313*), as the hydrogen bonds provide directional driving forces and the specific functional group interactions further enhance structural ordering at the mouth of the COF membrane. While we acknowledge that direct structural characterization evidence cannot be provided in the current version of the manuscript, the consistency of our experimental results and the well-documented interactions between –NH_2 or –OH groups in CDN and–SO_3H in the COF strongly support the feasibility of this alignment. We also acknowledge that there is a certain probability that not all channels will achieve such an ideal distribution. For example, when an excessive amount of CDN is introduced (COF-CDN-15), dense interfacial assembly may lead to partial misalignment of the hourglass-shaped nanochannels. Such misalignment may disrupt the continuity of hourglass-shaped transport pathways, leading to larger transport resistance and lower membrane separation performance. In the revised manuscript, we have added relevant discussion about this, which could be found below.

Page 7, Lines 138-143

"The alignment of CDN relied on hydrogen bonding and electrostatic interactions between the amino (–NH_2) or hydroxyl (–OH) groups in CDN and the sulfonic acid (–SO_3H) groups in the COF[36,37]. These interactions not only stabilized CDN at the COF mouth but also promoted an ordered arrangement by providing directional forces and enhancing structural organization[38,39]." **was added**.

Page 17, Lines 369-370

"and dense interfacial assembly tended to cause partial misalignment of the hourglass-shaped nanochannels" **was added**.

Page S35

"When an excessive amount of CDN was introduced (COF-CDN-15), dense interfacial assembly was likely to cause partial misalignment of the hourglass-shaped nanochannels. The misalignment of the nanochannels disrupted the continuity of the water transport pathways, leading to larger transport resistance and lower membrane separation performance, as reflected by the initial increase and subsequent decrease in the water flux (Supplementary **Fig. 17**)." **was added**.

Comment R1-2: The reported increase in membrane thickness by approximately 10 times (from 0.19 to 1.26 microns) raises concerns about the potential increase in resistance. How does this significant change in thickness affect water flux and salt rejection? Specifically, how do the improvements in hydrophilicity and the hourglass-shaped channels with reduced channel size counterbalance the increased resistance caused by the thicker membrane?

Response R1-2: Thank the reviewer for the valuable guidance. While the membrane thickness increases significantly (from 0.19 to 1.26 μm), which could lead to an increase in resistance, we conduct a thickness-normalized water flux analysis to eliminate the effect of thickness and compare membrane performance under equivalent thickness conditions. That is, we have normalized the flux by multiplying it with the membrane thickness (*ACS Nano, 2022, 16, 2407−2418*, *Nat. Commun., 2021, 12, 507*). As shown in **Figure R1-2**, with the increase of CDN content, the formation of hourglass-shaped nanochannels is enhanced, accompanied by improved membrane hydrophilicity. The hourglass-shaped nanochannels effectively reduce the transport resistance, as evidenced by monotonically increasing thickness-normalized flux. Specifically, the wide hydrophilic entrance of the nanochannel facilitates water capture and substantially lowers the critical intrusion pressure (*Angew. Chem. Int. Ed., 2025,*

64, e202418896, Nat. Nanotechnol., 2022, 17, 188–193, Chem. Soc. Rev., 2018, 47, 322-356, Chem. Soc. Rev., 2010, 39, 1073–1095, Nature, 2001, 414, 188-190). The gradually converging conical region then guides the water flow smoothly, avoiding recirculation or turbulence typically associated with abrupt geometrical transitions. Once the water enters the narrow hydrophobic spout, weak wall–membrane interactions generate a slip boundary that significantly minimizes viscous friction, affording high axial velocity. Finally, the water flow then exits through a wide outlet, where the sudden expansion dissipates residual pressure and prevents back-pressure build-up, further reducing overall hydraulic resistance. At the same time, the increased membrane hydrophilicity (**Fig. 3**e) and surface roughness (Supplementary **Fig. 12**) provide additional interaction sites, while hydroxyl groups in CDN add more polar sites and fortify water–membrane interactions, thereby reducing transport resistance, allowing water molecules to spread more easily, and increasing membrane permeance. Although the COF-CDN-15 membrane exhibits a decrease in non-normalized water flux due to increased thickness and partial misalignment of nanochannels (Supplementary **Fig. 17**), its thickness-normalized flux continues to increase. This is because, under equivalent thickness, the high CDN content introduces a higher density of hourglass-shaped nanochannels, which enhances water transport efficiency. Therefore, despite structural irregularities at high loading (COF-CDN-15), the membrane still demonstrates high water flux when the effect of thickness is normalized. This hourglass-shaped structural configuration facilitates fast and low-resistance water transport, which compensates for the increased path length from membrane thickness. As a result, the membrane maintains high water flux, while the narrow, charged throat ensures effective ion rejection. This demonstrates that rational nanochannel design can successfully overcome the traditional linear relation between membrane thickness and transport resistance. In the revised manuscript, we have added relevant discussion about this, which could be found below.

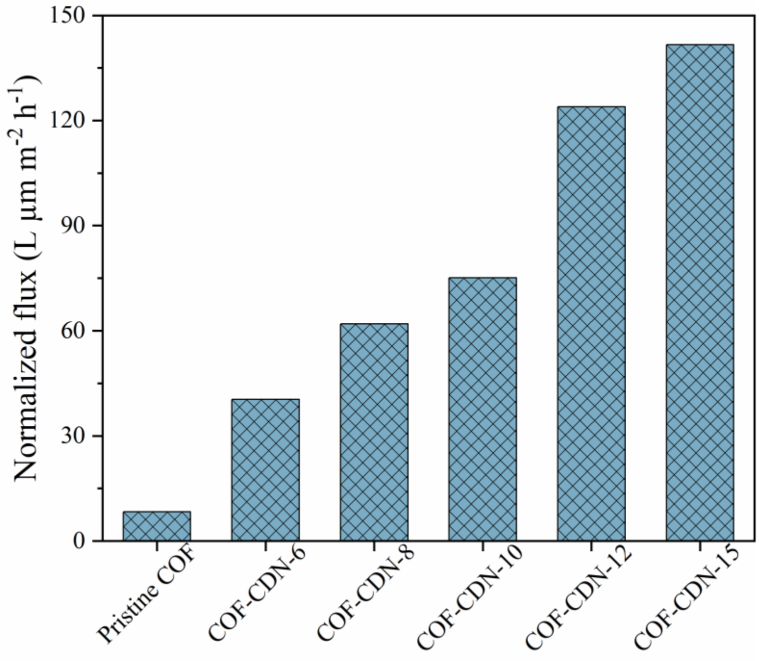

Figure R1-2. Thickness-normalized water flux of different membranes.

Page 12, Lines 254-258

"The hydrophilic conical entrance facilitated the entry of water, while the hydrophobic spout and the homo-nanochannels ensured low friction and fast water transport[18]." **was revised to** "The hydrophilic conical entrance facilitated the entry of water by lowering the critical intrusion pressure, while the gradually narrowing geometry guided flow and prevented turbulence. The hydrophobic spout, together with the homo-nanochannels, created a slip boundary that minimized viscous friction, thereby enabling low-resistance and fast water transport[18].".

Page 17, Lines 367-371

"This was because the separation layer of the COF-CDN-15 membrane became thicker (Supplementary **Fig. 9**), exposing less space for water permeation and leading to a decrease in membrane water flux (Supplementary **Fig. 17**)." **was revised to** "This was because the separation layer of the COF-CDN-15 membrane became thicker (Supplementary **Fig. 9**), and dense interfacial assembly tended to cause partial misalignment of the hourglass-shaped nanochannels, leading to a decrease in membrane water flux (Supplementary **Fig. 17**).".

Page 17, Lines 371-377

"The flux was normalized by multiplying it with the membrane thickness to eliminate the effects of thickness variations[46]. With the increase of CDN content, the formation of hourglass-shaped nanochannels was enhanced, thereby facilitating fast and low-resistance water transport, as confirmed by monotonically increasing thickness-normalized flux (Supplementary **Fig. 26**), despite the increased membrane thickness and partial misalignment of the hourglass-shaped nanochannels." **was added**.

Page S35

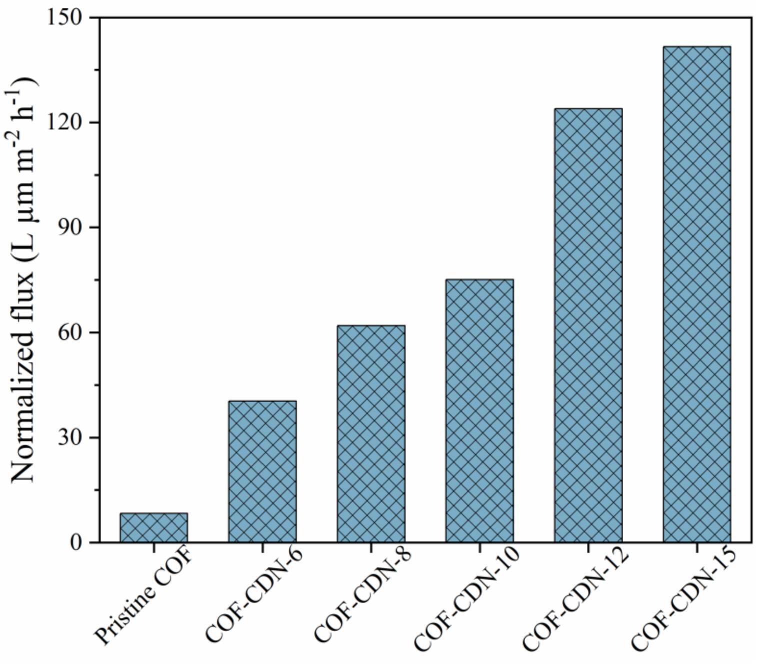

"

Supplementary Figure 26. Thickness-normalized water flux of different membranes.

Note: The flux was normalized by multiplying it by the membrane thickness to eliminate the effects of thickness variations. With the increase of CDN content, the formation of hourglass-shaped nanochannels was enhanced, accompanied by improved membrane hydrophilicity. These hourglass-shaped nanochannels effectively reduced the transport resistance, thereby facilitating fast and low-resistance water transport, as evidenced by monotonically increasing thickness-normalized flux. When an excessive amount of CDN was introduced (COF-CDN-15), dense interfacial assembly was likely to cause partial misalignment of the hourglass-shaped nanochannels. The misalignment

of the nanochannels disrupted the continuity of the water transport pathways, leading to larger transport resistance and lower membrane separation performance, as reflected by the initial increase and subsequent decrease in the water flux (Supplementary **Fig. 17**)." **was added**.

Comment R1-3: Following the above, the modelling of the channel size in the two states of COF-CDN membranes does not sufficiently explain the observed enhancements in water flux and salt rejection compared to the COF membrane alone. To provide stronger support for the desalination performance, it would be more informative to include a detailed analysis of the interactions between functional groups and water/salt molecules as they pass through the hourglass channels formed by COF-CDN with varying properties across the channels. Additionally, molecular dynamics (MD) simulations of water and salt transport across these membranes over time could offer more direct insights. The change in thickness between COF membranes and COF-CDN membranes should be factored in. Current modelling of the change in pore size of amino-cyclodextrin at different pH, which is known and previously reported, adds limited value.

Response R1-3: Based on the reviewer's valuable guidance, we carried out MD simulations that explicitly trace the transport of water and salt ions across the COF-CDN hourglass-shaped nanochannels at pH values of 3 and 11. These simulations capture the dynamic interactions between water/ions and the functional groups along the hourglass-shaped nanochannels, demonstrating the pH-responsive property. Stronger interactions between water/ions and the functional groups in the protonated hourglass-shaped nanochannels of COF-CDN hinder molecular transport, leading to the experimentally observed low water flux and high salt rejection under acidic conditions. In addition, we would like to emphasize that our experimental results support the dominant role of the hourglass-shaped nanochannels in facilitating water transport. As shown in the thickness-normalized flux data (Supplementary **Fig. 26**), increasing CDN content enhances the formation of hourglass-shaped nanochannels and improves membrane hydrophilicity. These features effectively reduce transport

resistance, thereby enabling fast and low-resistance water transport, as evidenced by monotonically increasing thickness-normalized flux. A detailed discussion is provided in our response to Comment R1-2 and Comment R1-11. Therefore, the current MD simulations focus on the hourglass-shaped nanochannels and do not account for the full membrane thickness. While this limitation may underestimate the total resistance, the simulated trends in water flux and ion rejection qualitatively agree with the experimental data, particularly regarding the pH-responsive behavior and the enhanced performance of COF-CDN-12. In the revised manuscript, we have added relevant discussion, which could be found below.

Page 16, Lines 350-358

"The MD simulation results (Supplementary **Fig. 24**) provided visualized evidence of water and salt transport through hourglass-shaped nanochannels. While water diffusion was reduced in the protonated state compared to the deprotonated state, no salt ion transport was detected under either condition. The interactions between water/ions and the functional groups as they passed through the hourglass-shaped nanochannels formed by COF-CDN were analyzed (Supplementary **Fig. 24**b-c). Stronger interactions (i.e., more negative values) in the protonated state hindered water/salt molecular transport, which accounted for the experimentally observed low water flux and high salt rejection under acidic conditions." **was added**.

Page S5

"**Molecular dynamics simulations**

All-atom molecular dynamics (MD) simulations were performed using the Gromacs-2023.6 software package[2] with the general AMBER force field[3] and RESP charges[4]. The simulated system was initialized as a relaxed liquid configuration at 298 K. Prior to the equilibrium MD simulations, energy minimization was conducted using a steepest descent algorithm with a convergence criterion set to a gradient of 100 kJ mol^{-1} nm^{-1}. Subsequently, the system underwent equilibration through a 3000 ps NVT simulation maintained at 298 K by employing a Nose´-Hoover thermostat[5]. Periodic

boundary conditions were applied in all three spatial dimensions. Long-range electrostatic interactions were calculated using the Particle Mesh Ewald (PME) method[6,7] with a relative tolerance of 1×10^{-6} and a real-space cut-off distance of 1 nm. The van der Waals interactions were truncated at the same cut-off. Bond lengths involving hydrogen atoms were constrained using the LINCS algorithm[8]. A leap-frog integrator[9] with a 2×10^{-3} ps time step was used to advance the simulation. Additionally, a virtual wall was implemented along the z-direction to prevent water molecules from crossing the boundaries of the simulation box." **was added**.

Page S31-32

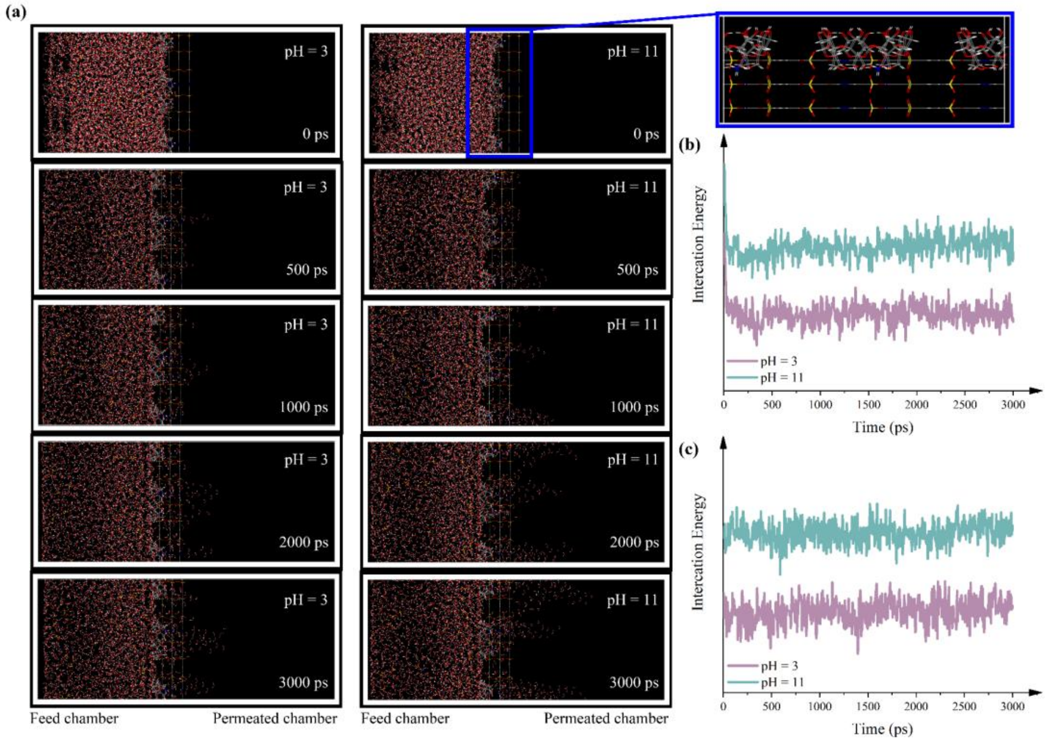

"

Supplementary Figure 24. (a) Simulation snapshots of Na_2SO_4 solution transporting through the hourglass-shaped nanochannels at 0 ps, 500 ps, 1000 ps, 2000 ps and 3000 ps. Interaction energy between the membranes and (b) water or (c) Na_2SO_4 solution.

Note: To validate these transport characteristics, all-atom MD simulations were performed to explicitly track the movement of water molecules and ions across the COF-CDN membrane separating the feed and permeate reservoirs. The results showed that, under neutral or alkaline conditions (i.e., with deprotonated hourglass-shaped nanochannels), water molecules were able to rapidly diffuse through the hourglass-

shaped nanochannels, with a large number of molecules traversing from the feed side to the permeate side within a short simulation period. In contrast, under acidic conditions (i.e., with protonated hourglass-shaped nanochannels), only a few water molecules were observed to pass through the channel during the same timeframe (Supplementary **Fig. 24**a), consistent with the experimentally observed low water flux and high salt rejection under acidic conditions.

Moreover, no salt ions were observed to permeate the membrane within the simulation timescale under either condition, which was consistent with the experimentally observed high salt rejection. The interactions between functional groups and water/salt molecules as they passed through the hourglass-shaped nanochannels formed by COF-CDN were analyzed (Supplementary **Fig. 24**b-c). The results revealed that both water and Na_2SO_4 exhibited stronger interaction energy (i.e., more negative values) with the protonated hourglass-shaped nanochannels. These stronger interactions hindered the passage of water molecules and salt ions through the hourglass-shaped nanochannels, thereby contributing to the experimentally observed lower water flux and higher salt rejection under acidic conditions." **was added**.

Comment R1-4: Generally, the increase in the roughness and amino groups on the surface could lead to more significant fouling, while increased hydrophilicity would reduce the surface interaction with the foulants. It is suggested that fouling experiments should be performed to determine these effects.

Response R1-4: Thank the reviewer for the valuable guidance. We agree that increased surface roughness and the presence of amino groups can, in some cases, promote fouling due to enhanced surface interactions. Hence, we added fouling experiments using humic acid (HA) and bovine serum albumin (BSA) as model foulants. As shown in Supplementary **Fig. 18**, the COF-CDN-12 membrane exhibits higher flux recovery rates (FRR: 94.1% for HA and 95.8% for BSA) compared with the pristine COF membrane (FRR: 92.7% for HA and 92.3% for BSA), indicating improved antifouling performance. Because the introduction of CDN not only introduces amino groups but also hydroxyl groups to improve the membrane's surface hydrophilicity, forming a

stable hydration layer through hydrogen bonding between the hydrophilic groups on CDN and water molecules, which acted as an effective antifouling barrier (*J. Am. Chem. Soc. 2021, 143(40), 16786–16795*). Additionally, the increased surface roughness provides more binding sites for water molecules, further strengthening the hydration layer (*Nat. Commun., 2024, 15, 6157*). These results confirm that the positive effects of enhanced hydrophilicity outweigh the potential fouling risks associated with increased roughness and amino functionality. We have revised the relevant sections of the manuscript to address this concern and improve the overall clarity.

Page 13, Lines 263-267

"As shown in Supplementary **Fig. 18**, the flux recovery rate (FRR) exceeded 90% for both humic acid (HA) and bovine serum albumin (BSA), demonstrating excellent antifouling performance. Notably, the introduction of CDN further improved the antifouling properties, with the FRR of the COF-CDN-12 membrane reaching 94.1% (HA) and 95.8% (BSA)." **was added**.

Page S4

"**Antifouling properties of COF-CDN membranes**

The antifouling properties of COF-CDN membranes were investigated using HA and BSA solution (1000 ppm). Initially, to ensure stable permeance, water was filtered through the membrane for 0.5 h at 2.5 bar, followed by another 0.5 h of water filtration at 2.0 bar, and the initial water flux was recorded as J_{w1} (L m^{-2} h^{-1}). Subsequently, a model foulant solution (HA or BSA) was filtered through the membrane for 1.0 h at 2.0 bar, and the resulting flux was recorded as J_p (L m^{-2} h^{-1}). Finally, the membrane that had been subjected to the foulant filtration test was cleaned with deionized water for 0.5 h, followed by another 0.5 h of water filtration at 2.0 bar, with the resulting flux recorded as J_{w2} (L m^{-2} h^{-1}). The antifouling properties were evaluated using the flux recovery rate (*FRR*), defined as follows:

$$FRR = \frac{J_{W2}}{J_{W1}} \times 100\% \quad (1)$$

" **was added**.

Page S24

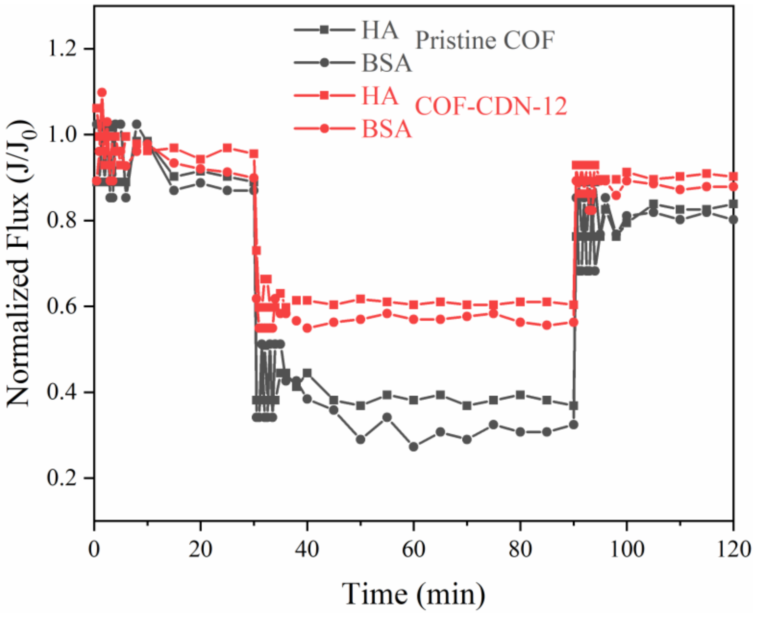

"

Supplementary Figure 18. Time-dependent normalized flux of the pristine COF and COF-CDN-12 membranes using different model foulant solutions.

Note: Antifouling properties of the pristine COF and COF-CDN-12 membranes were evaluated using the flux recovery rate (FRR) after filtering model foulant solutions of humic acid (HA) or bovine serum albumin (BSA) (Supplementary **Fig. 18**). FRR of the pristine membrane was 92.7% (HA) and 92.3% (BSA), with the introduction of CDN to membrane (COF-CDN-12), the antifouling properties of the membranes improved, FRR of the COF-CDN-12 membrane was 94.1% (HA) and 95.8% (BSA). Notably, the introduction of CDN further improved the antifouling properties, which was attributed to the improved surface hydrophilicity and the formation of a stable hydration layer through strong hydrogen bonding between the hydrophilic groups on CDN and water molecules. This hydration layer acted as an effective antifouling barrier by preventing foulant adsorption[12], while the increased surface roughness further reinforced it by enlarging the membrane surface area and providing additional water-binding sites[13]." **was added**.

Comment R1-5: The capillary effect induced by the differences in hydrophilicity in

Line 197 is unclear. Is this effect supported experimentally or consistent with the findings from the literature?

Response R1-5: Thank the reviewer for the valuable guidance. In Lines 200-201 we refer to the capillary effect induced by the asymmetric wettability of the hourglass-shaped nanochannels, which consist of a hydrophilic entrance and a hydrophobic spout. This structural asymmetry facilitates directional water transport by reducing the energy barrier for entering into the hydrophilic entrance and promoting rapid drainage through the hydrophobic constriction, consistent with the reported in literature (*Angew. Chem. Int. Ed., 2025, 64, e202418896*, *Nat. Nanotechnol., 2022, 17, 188–193*). We have changed the relevant sections of the manuscript to improve the overall clarity.

Page 9, Lines 200-201

"Furthermore, the hourglass-shaped nanochannels, which consisted of the hydrophilic entrance and the hydrophobic spout, facilitated capillary effect, reduced flow resistance" **was revised to** "Furthermore, the hourglass-shaped nanochannels, which consisted of the hydrophilic entrance and the hydrophobic spout, reduced flow resistance[24].".

Comment R1-6: The FT-IR analysis, water contact angle, and zeta potential measurements conducted using different sets of samples with varying CDN loadings. In FT-IR, the CDN loading is 4, 7, 10, 13, 16, while it is 8, 10, 12, 15 for water contact angle, and 6, 12, 15 for zeta potential. The same range of samples should be used to aid comparison. Additionally, please check if "COF-CD" in Fig 3c should read "COF-CDN".

Response R1-6: Thank the reviewer for the valuable guidance. We acknowledge that using a consistent range of CDN loadings for FT-IR analysis, water contact angle, and zeta potential measurements would facilitate direct comparison. In the revised manuscript, we have added the dynamic contact angle data for COF-CDN-6, as well as the zeta potential data for COF-CDN-8 and COF-CDN-10. Additionally, we have carefully rechecked **Fig. 3**c and discovered that the originally included image was

unrelated, and we replaced it with the correct image corresponding to the COF-CDN membrane. This correction does not affect the results or conclusions of the study. **Fig. 3** has also been revised to improve clarity in the revised manuscript.

Page 11, Line 217, Fig. 3 has been revised to:

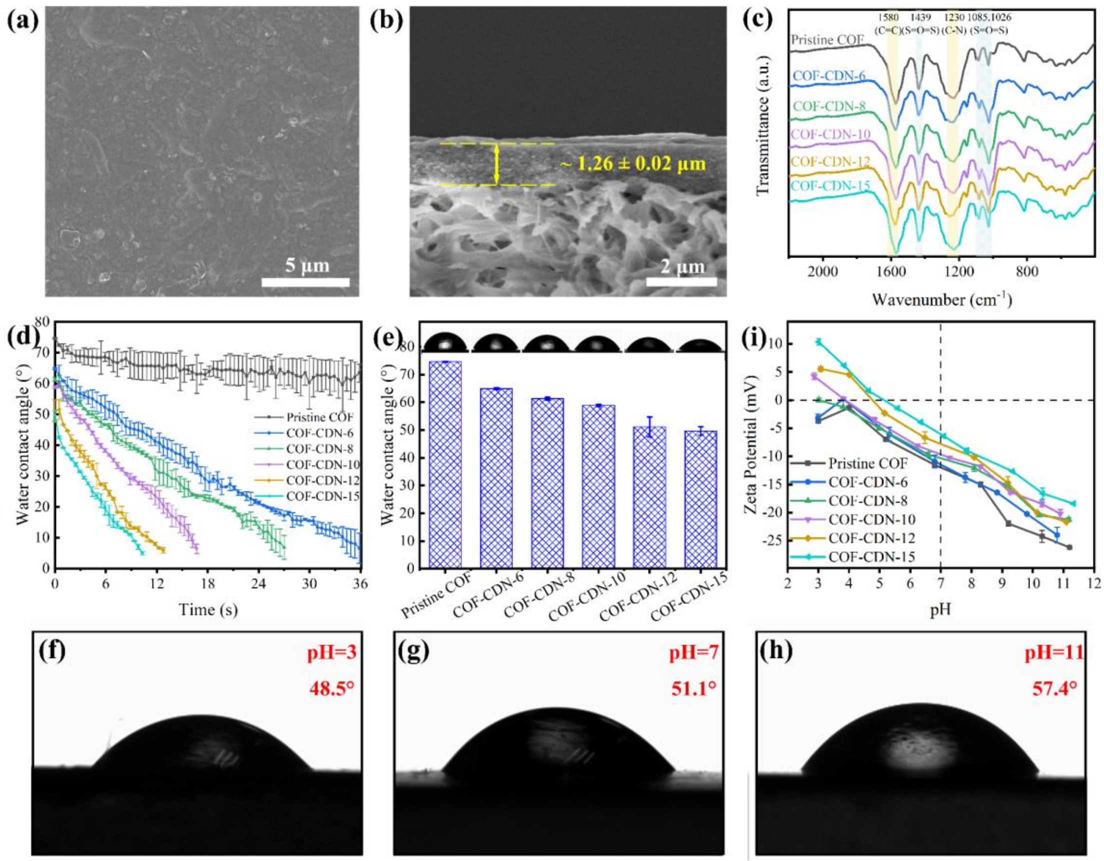

Comment R1-7: It is obvious that the salt rejection decreases at each cycle in the crossflow filtration test. The authors should revise line 348 to reflect this.

Response R1-7: Thank the reviewer for the valuable guidance. We are sorry for the mistake, and we have revised the relevant sections in the revised manuscript to enhance the accuracy of our description.

As shown in **Fig. 6**b and **Supplementary Fig. 28**b-d, a slight decline in rejection is observed at pH 3 or pH 11 during the cycling test, in which the pH value is shifted between 3 to 11. This can be attributed to partial retention of salt ions within the membrane pores (*Chem. Soc. Rev., 2022, 51, 2224-2254*, *Nat. Commun., 2022, 13,*

7339). Specifically, when the pH shifts from 3 to 11, the pores of COF-CDN membranes enter an “open” state, allowing salt ions to pass through more easily. Some of these salt ions become retained within the membrane pores. When the pH returns from 11 to 3, these residual salt ions interfere the complete re-protonation of COF-CDN membranes, resulting in a slight decline in rejection during the subsequent cycle.

Page 19, Lines 405-407

"Furthermore, after three cycles of pH value shifting from 3 to 11, the COF-CDN-12 membrane maintained consistent rejection for Li_2SO_4, Na_2SO_4, LiCl, NaCl, with no significant decrease in performance observed at either pH value of 3 or 11, as shown in **Fig. 6**b and Supplementary **Fig. 22**." **was revised to** "In contrast, as shown in Fig. 6b and Supplementary **Fig. 28**b-d, rejection for Li_2SO_4, Na_2SO_4, LiCl, NaCl exhibited a slight decline at either pH value of 3 or 11, due to partial retention of salt ions within the membrane pores[47,48]."

Page 19, Lines 407-411

"This result demonstrated the reversible and reliable pH-responsive property of the COF-CDN-12 membrane, confirming its robust structural integrity, which was crucial for maintaining stable separation under fluctuating pH values." **was revised to** "The combination of water flux stability and the limited rejection decrease demonstrated the reversible and reliable pH-responsive property of the COF-CDN-12 membrane, confirming its robust structural integrity, which was crucial for maintaining stable separation under fluctuating pH values."

Page S37

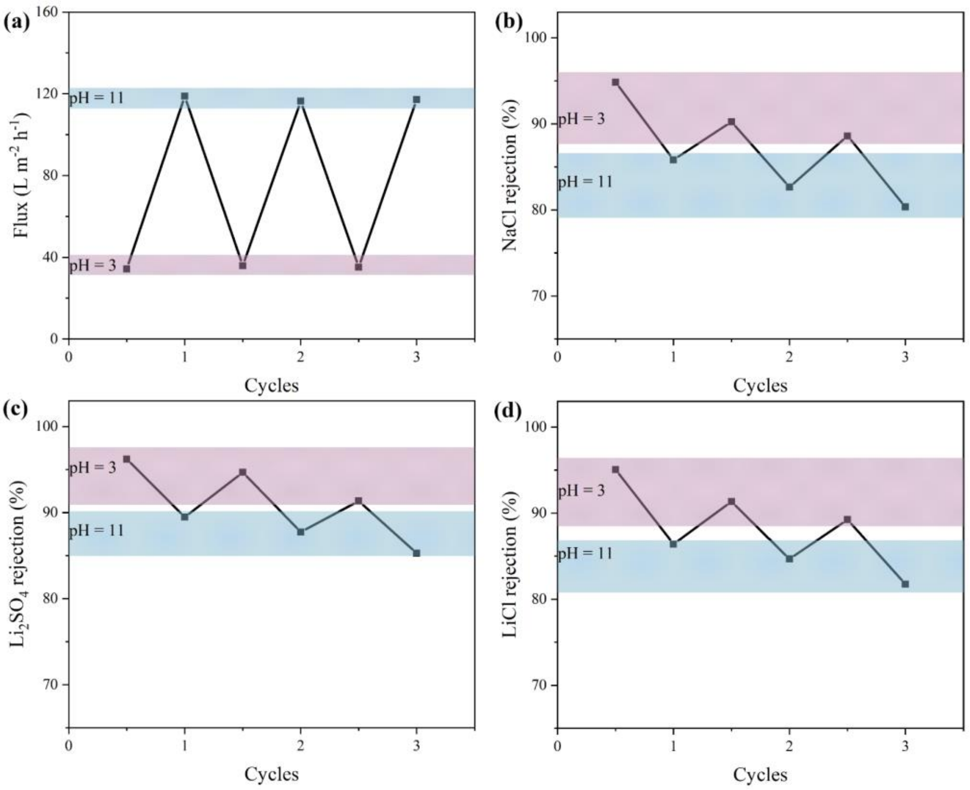

"

Supplementary Figure 28. (a) Flux, (b) NaCl, (c) Li_2SO_4 and (d) LiCl rejection of COF-CDN-12 membrane when the pH shifted from 3 to 11 after 3 cycles.

Note: As shown in Supplementary **Fig. 28**a, the COF-CDN-12 membrane maintained consistent water flux, with no significant decrease in performance observed at either pH value of 3 or 11. As shown in **Fig. 6**b and Supplementary **Fig. 28**b-d, a slight decline in rejection was observed at pH 3 or pH 11 during the cycling test, in which the pH value was shifted between 3 to 11. This was attributed to partial retention of salt ions within the membrane pores. Specifically, when the pH shifted from 3 to 11, the pores of COF-CDN membranes entered an “open” state, allowing salt ions to pass through more easily. Some of these salt ions were retained within the membrane pores. When the pH returned from 11 to 3, these residual salt ions interfered with the complete re-protonation of COF-CDN membranes, resulting in a slight decline in rejection during the subsequent cycle." **was added**.

Comment R1-8: The membrane desalination performance in realistic conditions (relevant pressure, mixed salt solutions) should be evaluated.

Response R1-8: Thank the reviewer for the valuable guidance. The membrane desalination performance in realistic conditions is very important, hence, we performed separation tests using a mixed salt solution of NaCl and $MgCl_2$ (each at a concentration of 1000 ppm). The rejections obtained from the mixed salt experiments are consistent with those observed in single salt solutions, indicating the membrane with effective ion rejection performance even in the mixed salt solution. These results have been added in the revised manuscript (Supplementary **Fig. 19**) to support the applicability of our membrane under realistic conditions. Moreover, recent advancements in desalination technologies aim to reduce these pressures and improve energy efficiency (*Nano Lett., 2023, 23(23), 10884–10891, Environ. Sci. Technol. 2018, 52, 4, 2242–2250*). The membrane is designed to operate efficiently under low-pressure conditions (*Nat. Rev. Methods Primers, 2024, 4, 10*). In our experiments, a pressure of 2.0 bar was used as the driving force, under which we achieved a water flux of 98 $L \cdot m^{-2} \cdot h^{-1}$, demonstrating excellent permeability and energy efficiency.

Page 13, Lines 271-274

"The membrane exhibited consistent ion rejection performance in a mixed salt solution (NaCl and $MgCl_2$, 1000 ppm each), comparable to that observed in single salt solutions (Supplementary **Fig. 19**)." **was added**.

Page S26

"
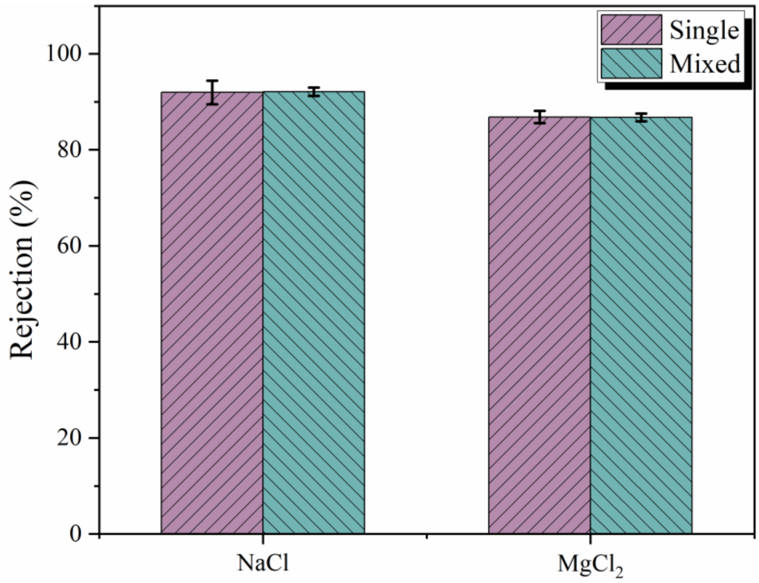

Supplementary Figure 19. Rejection of the COF-CDN-12 membrane in single and mixed salt solutions.

Note: To further simulate realistic desalination conditions, we performed separation tests using a mixed salt solution of NaCl and $MgCl_2$ (each at a concentration of 1000 ppm). The rejections obtained from the mixed salt experiments were consistent with those observed in single salt solutions, indicating the membrane effective ion rejection performance even in the mixed salt solution." **was added**.

Comment R1-9: Line 295-300: The explanation of ion transmembrane behavior using the Donnan effect in this section may not be entirely accurate. After pH adjustment, the functional groups on the membrane surface change from $-NH_2$ to $-NH_3^+$, resulting in a positively charged membrane surface. This surface charge should affect both anions and cations: for cations, higher-valent cations such as Mg^{2+} would experience greater electrostatic repulsion; for anions, higher-valent anions such as SO_4^{2-} would exhibit stronger attraction. Due to the strong electrostatic attraction of divalent anions by the membrane, compared to monovalent anions, a stronger negatively charged anion concentration layer may form on the membrane surface, which would further repel anions in the solution. To achieve a more comprehensive interpretation, both cation and anion interactions should be considered simultaneously.

Response R1-9: Thank the reviewer for the valuable guidance. We agree that the initial

interpretation based solely on the Donnan exclusion effect was incomplete. As the reviewer pointed out, after protonation (-NH_2 to -NH_3^+), the positively charged membrane surface not only reject cations but can also attract anions, particularly divalent anions like SO_4^{2-}. This attraction could lead to the formation of a negatively charged anion concentration layer at the membrane surface, which may subsequently enhance the rejection of additional anions from the solution. To provide a more comprehensive understanding, we performed DFT calculations that simultaneously consider cation and anion interactions in both protonated and deprotonated states, as shown in **Figure R1-9**, to support the when interpretation of ion rejection. The calculated overall binding energy (Li_2SO_4 > Na_2SO_4 > $MgSO_4$ > LiCl > NaCl > $MgCl_2$) reflects the combined contributions of both anion and cation affinities toward the membrane, indicating that ion rejection is controlled by interactions involving both cations and anions, rather than only by Donnan exclusion.

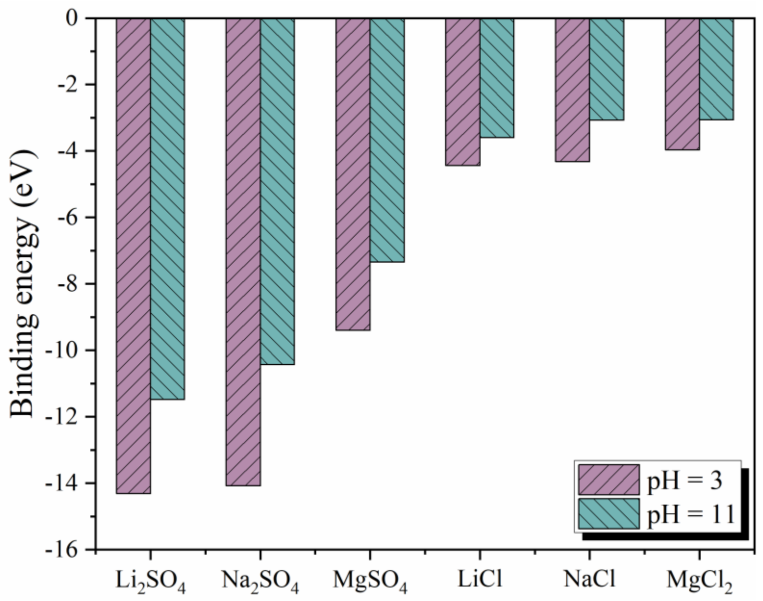

Figure R1-9 Binding energy between salt molecule and CDN at different pH values calculated by DFT

Page 15, Lines 311-318

"This effect was especially pronounced for multivalent anions, such as SO_4^{2-}, which experienced greater electrostatic repulsion than monovalent anions, such as Cl^-, resulting in consistently higher rejection for Na_2SO_4 compared to NaCl." **was revised**

to "This electrostatic phenomenon was particularly significant for multivalent anions such as SO_4^{2-}, which experienced stronger repulsive forces compared to monovalent anions like Cl^-. The initial attraction of divalent anions to the positively charged membrane surface was proposed to result in the formation of a concentrated counterion layer, which subsequently amplified the repulsion of additional incoming anions from the solution. This mechanism was considered to contribute to the consistently higher rejection of Na_2SO_4 relative to NaCl under acidic conditions.".

Page 16, Lines 347-350

"Meanwhile, DFT calculations considering both cation and anion interactions under protonated and deprotonated states indicated that ion rejection was controlled by interactions involving both cations and anions, rather than only by Donnan exclusion (Supplementary **Fig. 23**)." **was added**.

Page S5

"**Density functional theory calculation**

Quantum chemical calculations were performed using the Gaussian 09W software package[1] based on density functional theory (DFT). All molecular geometries were fully optimized at the hybrid B3LYP functional level employing the 6-31G (d, p) basis set, without imposing symmetry constraints. The optimized geometries were confirmed as global minima by frequency calculations, which indicated the absence of imaginary frequencies. Additionally, dispersion interactions were accounted for using Grimme's D3 correction method (B3LYP+D3). The basis set superposition error (BSSE) was evaluated and corrected to accurately estimate intermolecular interaction energies." **was added**.

Page S30

"
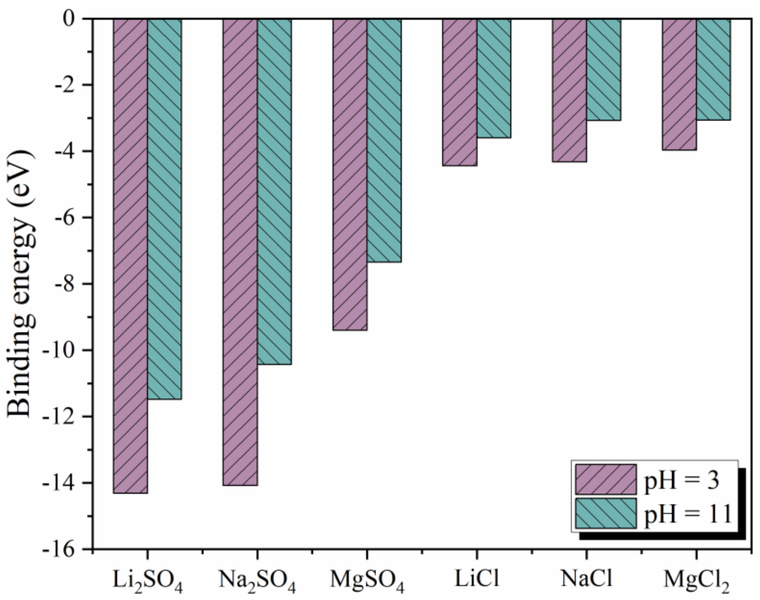

Supplementary Figure 23. Binding energy between salt and CDN at different pH values calculated by DFT.

Note: Meanwhile, DFT calculations were conducted considering both cation and anion interactions under protonated and deprotonated states. The overall binding energy sequence (Li_2SO_4 > Na_2SO_4 > $MgSO_4$ > LiCl > NaCl > $MgCl_2$) was calculated, reflecting the combined contributions of anion and cation affinities toward the membrane. These results indicated that ion rejection was controlled by interactions involving both cations and anions, rather than only by Donnan exclusion. This behavior was attributed to the fact that, after protonation ($-NH_2$ to $-NH_3^+$), the membrane surface became positively charged, which not only repelled cations but also attracted anions, particularly divalent anions such as SO_4^{2-}. The electrostatic attraction of anions could lead to the formation of a negatively charged anion concentration layer at the membrane surface, which might subsequently enhance the rejection of additional anions from the solution." **was added**.

Page S33

"At lower pH values, the amino groups on CDN were protonated, which imparted a positive charge to the membrane surface. The positively charged membrane surface influenced ion distribution within the nanochannels, reducing the passage of ions. In addition, the protonation caused the CDN cavity to shrink, with the hydrophilic conical

entrance size of 16.1 Å and the hydrophobic spot size of 4.7 Å, causing the pores of COF-CDN membranes to either decrease in effective size or enter a "closed" state." **was revised to** "After protonation (-NH_2 to -NH_3^+), the membrane surface became positively charged, which not only rejected cations but also attracted anions. This attraction could lead to the formation of a negatively charged anion concentration layer at the membrane surface, which could subsequently enhance the rejection of additional anions from the solution. In addition, under acidic conditions, the amino groups in CDN were protonated, causing the CDN cavity to shrink, with the hydrophilic conical entrance size of 16.1 Å and the hydrophobic spot size of 4.7 Å. Consequently, the pore of COF-CDN membranes either decreased in effective size or enter a "closed" state.".

Comment R1-10: Line 236-247: The discussion of ion rejection in this section with the related Supporting Information Figure 17 requires further verification. According to the membrane pore size and hydrated ion diameters provided in the manuscript, ions should permeate the membrane through a dehydration mechanism. Based on this mechanism, sodium ions (Na^+) should exhibit the highest rejection rate due to their dehydrated diameter, followed by magnesium ions (Mg^{2+}), and lithium ions (Li^+). However, the data presented in the article indicate the highest rejection rate for Li^+. If hydrated diameters alone governed rejection rates, Mg^{2+} (with the largest hydrated size) should show the highest rejection, followed by Li^+ and then Na^+ (smallest hydrated size). This discrepancy may arise from unaccounted factors, such as ion dehydration energy due to varying surface properties across the ion channels.

Response R1-10: Thank the reviewer for the valuable guidance. The observed rejection sequence displays only a weak correlation with the fully hydrated ionic diameters, indicating that the cations undergo partial, rather than complete, dehydration within the hourglass-shaped nanochannels. Li^+ and Na^+ retain a substantial fraction of their hydration shells, and their larger hydrated diameters therefore result in the highest exclusion for Li^+, followed by Na^+. By contrast, Mg^{2+}—although it possesses the largest hydrated diameter in solution—loses a greater proportion of its hydration water in the hydrophobic constriction, acquires a smaller effective diameter, and consequently

traverses the membrane more readily, resulting in the lowest rejection among the examined salts.

Moreover, differences in the interaction between CDN and the individual cations also influence the observed rejection pattern. To quantify this effect, we performed DFT calculations on both the protonated and deprotonated states of CDN, evaluating their binding energies with salt ions. The binding energies between CDN and the cations consistently follow the order $Li^+ > Na^+ > Mg^{2+}$ (**Fig. 4**d) at both pH 3 and pH 11, whereas for anions, the order is $SO_4^{2-} > Cl^-$ (**Fig. 4**e). The resulting binding energy effects are presented in detail in responses R1-9 and R1-12.

Page 15, Lines 329-346

"To further account for the energy barriers introduced by CDN-ion interactions, DFT calculations were conducted to quantify the binding energy between the membrane and various salt ion. As shown in **Fig. 4**d-e, the influence of pH on membrane performance became particularly evident when comparing the two states. Under acidic conditions (pH = 3), the amino groups on CDN were protonated, thereby fortifying ion–membrane interactions. The calculated binding energies under pH 3 were –3.06 eV for Li^+, –2.95 eV for Na^+, –1.22 eV for Mg^{2+}, –8.19 eV for SO_4^{2-}, and –1.38 eV for Cl^-. The enhanced affinity raised the energy barrier for ion passage and contributed to improving ion rejection. Conversely, under alkaline conditions (pH = 11), the amino groups were deprotonated, which weakened these interactions and effectively lowered the steric and electrostatic resistance within the nanochannels. As a result, ions were more readily transported through the membrane. The calculated binding energies under pH 11 were –2.62 eV for Li^+, –2.09 eV for Na^+, –1.10 eV for Mg^{2+}, –6.24 eV for SO_4^{2-}, and –0.98 eV for Cl^-. Moreover, the binding energies between CDN and the cations consistently followed the order $Li^+ > Na^+ > Mg^{2+}$ (**Fig. 4**d) at both pH 3 and pH 11, whereas for anions, the order was $SO_4^{2-} > Cl^-$ (**Fig. 4**e). This binding hierarchy aligns well with the experimentally observed rejection sequence, indicating that ions exhibiting stronger affinity toward the membrane are more effectively retained." **was added**.

Page S22

"The observed rejection sequence displayed only a weak correlation with the fully hydrated ionic diameters, indicating that the cations undergo partial, rather than complete, dehydration within the hourglass-shaped nanochannels. Li^+ and Na^+ retained a substantial fraction of their hydration shells, and their larger hydrated diameters therefore result in the highest exclusion for Li^+, followed by Na^+. By contrast, Mg^{2+}—although it possessed the largest hydrated diameter in solution—lost a greater proportion of its hydration water in the hydrophobic constriction, acquired a smaller effective diameter, and consequently traversed the membrane more readily, resulting in the lowest rejection among the examined salts." **was added**.

Comment R1-11: Line 319-322: The discussion provided regarding the decreased pure water flux of the COF-CDN-15 membrane, attributed primarily to increased membrane thickness, may overlook additional factors. Previous data in the article show significant variations in membrane thickness. To isolate thickness effects, we recommend normalizing the flux by membrane thickness to compare the material performance under equivalent thickness conditions. A quick calculation based on the results in the manuscript reveal that the water flux of thickness-corrected COF-CDN-15 membranes remains lower than that of other membranes. While the data confirms COF-CDN-15's superior hydrophilicity compared to other membranes, this enhancement alone cannot fully account for the observed flux reduction. It may be worth exploring whether other factors, such as decreases in membrane pore size or increased thickness (both adding hydraulic resistance), contribute to this phenomenon.

Response R1-11: Thank the reviewer for the valuable guidance. As shown in Supplementary **Fig. 17**, the flux exhibits an initial increase followed by a decrease at higher CDN loadings (COF-CDN-15). We attribute this behavior to two main factors: (i) a substantial increase in membrane thickness, which elevated the resistance; and (ii) densification of the membrane structure along with dense interfacial assembly of CDN, which likely induced partial misalignment of the hourglass-shaped nanochannels. The misalignment of the nanochannels disrupted the continuity of water transport pathways,

leading to larger transport resistance and lower membrane separation performance. To eliminate the effect of thickness, we have normalized by multiplying it with membrane thickness (*ACS Nano, 2022, 16, 2407−2418*, *Nat. Commun., 2021, 12, 507*). As shown in **Figure R1-11**, with the increase of CDN content, the normalized flux still increased with higher CDN content. This is because, under equivalent thickness, the high CDN content introduces a higher density of hourglass-shaped nanochannels, which enhances water transport efficiency. Therefore, despite structural irregularities at high loading (COF-CDN-15), the membrane still demonstrates high water flux when the effect of thickness is normalized. In the revised manuscript and the supplementary information, we have added relevant discussion, which could be found below.

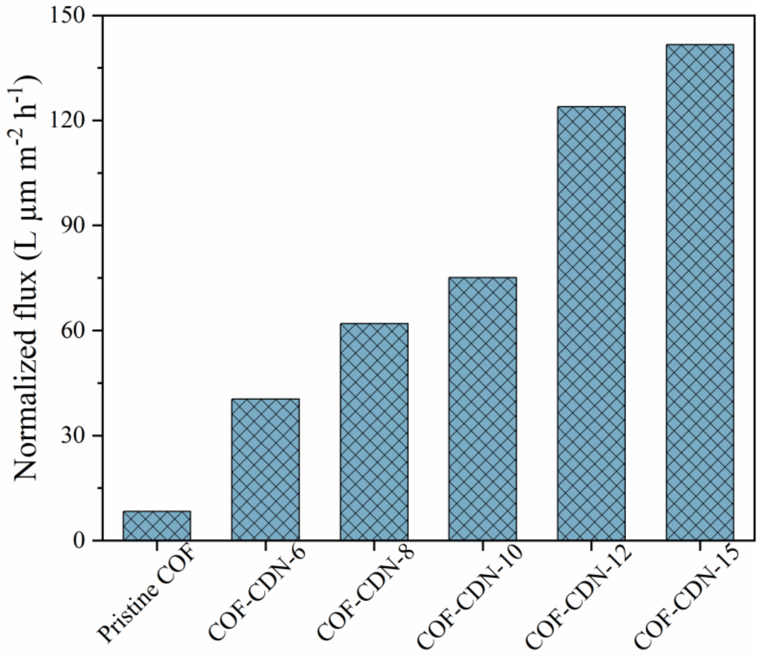

Figure R1-11. Thickness-normalized water flux of different membranes.

Page 17, Lines 367-371

"This was because the separation layer of the COF-CDN-15 membrane became thicker (Supplementary **Fig. 9**), exposing less space for water permeation and leading to a decrease in membrane water flux (Supplementary **Fig. 17**)." **was revised to** "This was because the separation layer of the COF-CDN-15 membrane became thicker (Supplementary **Fig. 9**), and dense interfacial assembly tended to cause partial misalignment of the hourglass-shaped nanochannels, leading to a decrease in membrane

water flux (Supplementary **Fig. 17**).".

Page 17, Lines 371-377

"The flux was normalized by multiplying it with the membrane thickness to eliminate the effects of thickness variations[46]. With increasing CDN content, the formation of hourglass-shaped nanochannels was enhanced, thereby facilitating fast and low-resistance water transport, as confirmed by monotonically increasing thickness-normalized flux (Supplementary **Fig. 26**), despite the increased membrane thickness and partial misalignment of the hourglass-shaped nanochannels." **was added**.

Page S35

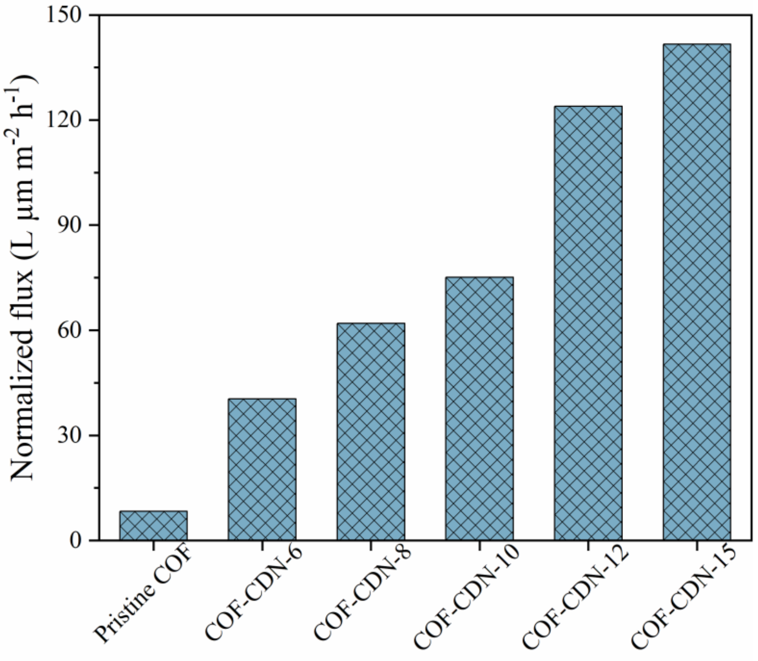

"

Supplementary Figure 26. Thickness-normalized water flux of different membranes. **Note:** The flux was normalized by multiplying it with the membrane thickness to eliminate the effects of thickness variations. With the increase of CDN content, the formation of hourglass-shaped nanochannels was enhanced, accompanied by improved membrane hydrophilicity. These hourglass-shaped nanochannels effectively reduced the transport resistance, thereby facilitating fast and low-resistance water transport, as evidenced by the monotonically increasing thickness-normalized flux. When an

excessive amount of CDN was introduced (COF-CDN-15), dense interfacial assembly was likely to cause partial misalignment of the hourglass-shaped nanochannels. The misalignment of the nanochannels disrupted the continuity of the water transport pathways, leading to larger transport resistance and lower membrane separation performance, as reflected by the initial increase and subsequent decrease in the water flux (Supplementary **Fig. 17**)." **was added**.

Comment R1-12: Overall, to provide a more accurate interpretation of the excellent desalination performance, computational simulation on the changes to the energy barrier and ion transport rate induced by CDN along with recently reported electrochemical characterization techniques for molecule/ion transport may provide more useful information.

Response R1-12: Thank the reviewer for the valuable guidance. In order to better understand the energy barriers introduced by CDN–ion interactions and their dependence on pH, we conduct DFT calculations to quantify the binding energies between the membrane and various salt ions. Specifically, we simulate both the protonated and deprotonated states of CDN to examine how pH-responsive behavior influences ion–membrane interactions and transport. As shown in **Figure R1-12**, the influence of pH on membrane performance becomes particularly evident when comparing the two states. Under acidic conditions (pH = 3), the amino groups on CDN are protonated, which fortifies the ion–membrane interactions. The calculated binding energies under pH 3 are –3.06 eV for Li^+, –2.95 eV for Na^+, –1.22 eV for Mg^{2+}, –8.19 eV for SO_4^{2-}, and –1.38 eV for Cl^-. The enhanced affinity increases the energy barrier for ion transport and contributes to improving ion rejection. In contrast, under alkaline conditions (pH = 11), deprotonation of the amino groups weakens these interactions, effectively lowering both steric and electrostatic resistance within the nanochannels and allowing ions to pass through more readily. Correspondingly, the calculated binding energies under pH 11 are –2.62 eV for Li^+, –2.09 eV for Na^+, –1.10 eV for Mg^{2+}, –6.24 eV for SO_4^{2-}, and –0.98 eV for Cl^-. These values reflect the diminished interaction strength in the deprotonated state and explain the decreased ion rejection performance

under alkaline conditions.

In addition, we fully agree that electrochemical characterization techniques are powerful tools for investigating molecule/ion transport in electrochemical membranes or devices. However, our study focuses on pressure-driven desalination membranes, where the primary separation mechanism relies on size exclusion and interaction-based rejection rather than electrochemical processes. Therefore, electrochemical characterization techniques were not considered in this work. Instead, we evaluated ion transport behavior through a combination of experimental tests (e.g., water flux and salt rejection under different pH conditions) and DFT calculations to analyze ion–membrane interactions.

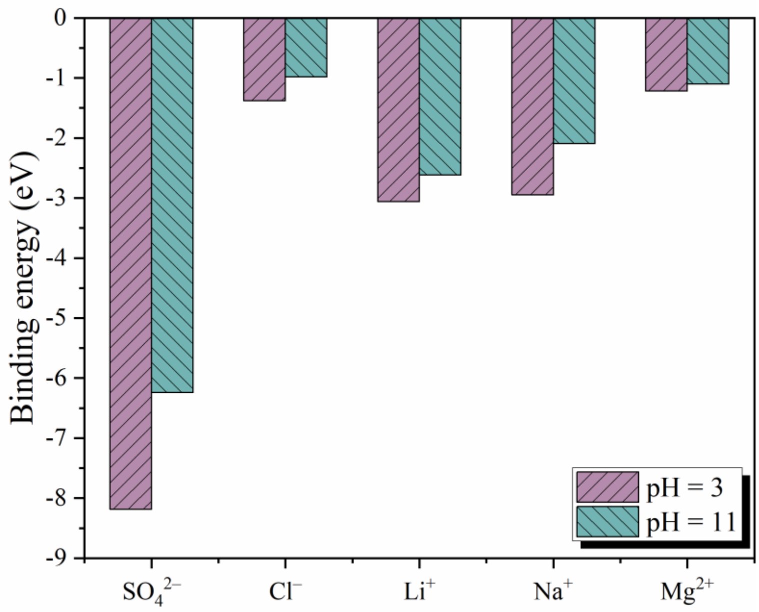

Figure R1-12. Binding energy between ions and CDN at different pH values calculated by DFT.

Page 15, Lines 329-346

"To further account for the energy barriers introduced by CDN-ion interactions, DFT calculations were conducted to quantify the binding energy between the membrane and various salt ion. As shown in **Fig. 4**d-e, the influence of pH on membrane performance became particularly evident when comparing the two states. Under acidic conditions (pH = 3), the amino groups on CDN were protonated, thereby fortifying ion–membrane interactions. The calculated binding energies under pH 3 were –3.06 eV for Li^+, –2.95

eV for Na^+, –1.22 eV for Mg^{2+}, –8.19 eV for SO_4^{2-}, and –1.38 eV for Cl^-. The enhanced affinity raised the energy barrier for ion passage and contributed to improving ion rejection. Conversely, under alkaline conditions (pH = 11), the amino groups were deprotonated, which weakened these interactions and effectively lowered the steric and electrostatic resistance within the nanochannels. As a result, ions were more readily transported through the membrane. The calculated binding energies under pH 11 were –2.62 eV for Li^+, –2.09 eV for Na^+, –1.10 eV for Mg^{2+}, –6.24 eV for SO_4^{2-}, and –0.98 eV for Cl^-. Moreover, the binding energies between CDN and the cations consistently followed the order $Li^+ > Na^+ > Mg^{2+}$ (**Fig. 4**d) at both pH 3 and pH 11, whereas for anions, the order was $SO_4^{2-} > Cl^-$ (**Fig. 4**e). This binding hierarchy aligns well with the experimentally observed rejection sequence, indicating that ions exhibiting stronger affinity toward the membrane were more effectively retained." **was added**.

Page S5

"**Density functional theory calculation**

Quantum chemical calculations were performed using the Gaussian 09W software package[1] based on density functional theory (DFT). All molecular geometries were fully optimized at the hybrid B3LYP functional level employing the 6-31G (d, p) basis set, without imposing symmetry constraints. The optimized geometries were confirmed as global minima by frequency calculations, which indicated the absence of imaginary frequencies. Additionally, dispersion interactions were accounted for using Grimme's D3 correction method (B3LYP+D3). The basis set superposition error (BSSE) was evaluated and corrected to accurately estimate intermolecular interaction energies." **was added**.

Comment R1-13: Please check Supplementary Figure 22 as both b and c are figures for LiCl rejection.

Response R1-13: Thank the reviewer for the valuable guidance. We are sorry for the mistake, and we have carefully checked the images. One LiCl rejection data image has been replaced with the correct Li_2SO_4 rejection data.

Page S37, Supplementary Figure 28 has been revised to:

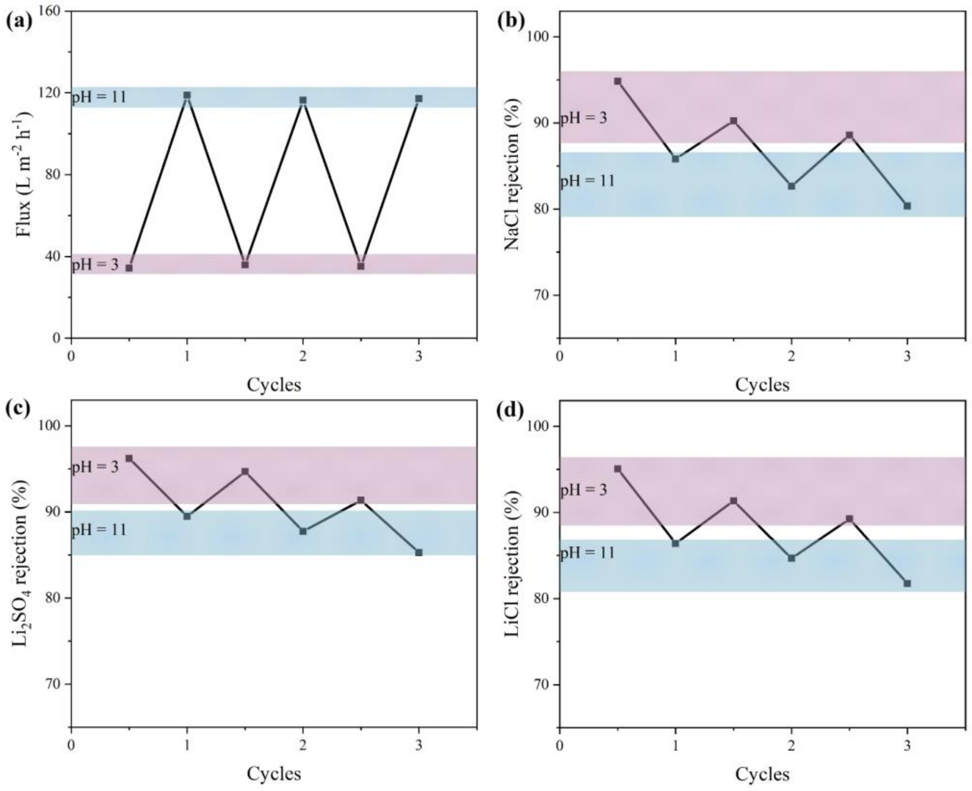

Comment R1-14: Please check and confirm whether the labels (Tp and $PaSO_3H$) in Supplementary Figure 1 correspond to their respective FT-IR spectra.

Response R1-14: Thank the reviewer for the valuable guidance. The labels (Tp and $PaSO_3H$) in Supplementary **Fig. 2** have been checked and confirmed to correspond to their respective FT-IR spectra. Supplementary **Fig.** 2 has been updated by adding peak assignments and corresponding wavenumber labels to enhance clarity in the revised manuscript and the supplementary information.

Page 5, Lines 91-93

"The FT-IR spectra (Supplementary **Fig. 2**) of the TpPa-SO_3H COF nanosheets revealed the disappearance of peaks corresponding to the amine groups at 3300–3400 cm^{-1} and the aldehyde groups at 2890 and 1650 cm^{-1}." **was revised to** "The FT-IR spectra (Supplementary **Fig. 2**) of the TpPa-SO_3H COF nanosheets revealed the disappearance of peaks corresponding to the amino groups at 3300–3450 cm^{-1} and the aldehyde groups at 2890 and 1650 cm^{-1}."

Page S6, Supplementary Fig. 2 has been revised to:

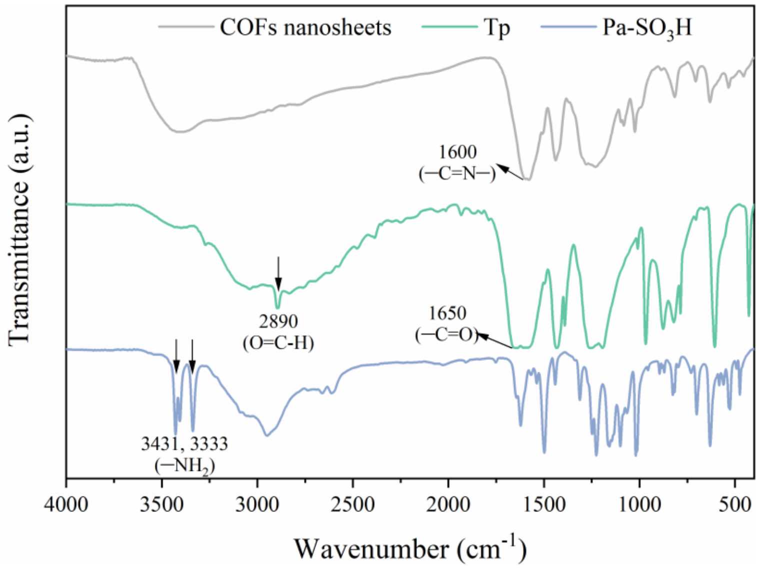

Comment R1-15: In Supplementary Figure 13, the data for COF-CDN-6-A and COF-CDN-6 in Figure (a), as well as COF-CDN-6 and COF-CDN-6-A in Figure (c), appear to be very similar.

Response R1-15: We sincerely appreciate the reviewer's meticulous attention to the details in Supplementary **Fig. 13**. FT-IR spectra for COF-CDN-6 and COF-CDN-6-A (treated with 1 M HCl for 24 hours) appear similar, which aligns with our experimental design and conclusions. Specifically, the membrane was soaked in 1 M NaOH and HCl for 24 hours to assess their chemical stability. The similarity in FT-IR spectra confirms that the membrane's chemical structure remains intact, demonstrating the excellent chemical stability of membrane under different pH environmental conditions. In the revised supplementary information, we have added a note addressing this issue, and the relevant details are provided below to avoid any confusion.

Page S18

"**Note:** After the treatment, FT-IR spectra indicated that the functional groups remained intact, thereby demonstrating the excellent chemical stability of membrane under different pH environmental conditions." **was added**.

Comment R1-16: It was not clear how the wettability test at different pH levels was performed.

Response R1-16: Thank the reviewer for the valuable guidance. The membranes after treatment in solutions at pH = 3, 7, and 11 for 24 h were used to investigate the effect of different pH values on membrane wettability. The information has been added in the revised supplementary information.

Page S4

"The membranes after treatment in solutions at pH = 3, 7, and 11 for 24 h were used to investigate the effect of different pH values on membrane wettability." **was added**.

Comment R1-17: The details of the crossflow velocity and membrane area used for crossflow filtration experiments are missing and should be provided.

Response R1-17: Thank the reviewer for the valuable guidance. During the cross-flow filtration experiment, the flow velocity was maintained at 72 mL h^{-1}, and the effective filtration area was 2.01×10^{-4} m^2, which has been added in the revised manuscript.

Page 22, Lines 478-480

"During the experiment, the flow velocity was maintained at 72 mL h^{-1}, and the effective filtration area was 2.01×10^{-4} m^2." **was added**.

Comment R1-18: The water flux data corresponding to Fig. 6b should be provided.

Response R1-18: Thank the reviewer for the valuable guidance. We have now provided the water flux of COF-CDN-12 membrane when the pH shifted from 3 to 11 after 3 cycles, as shown in **Figure R1-18**. After three cycles of pH value shifting from 3 to 11, the COF-CDN-12 membrane maintains consistent water flux, with no significant decrease in performance observed at either pH value of 3 or 11, confirming its robust structural integrity, which is crucial for maintaining stable separation under fluctuating pH values.

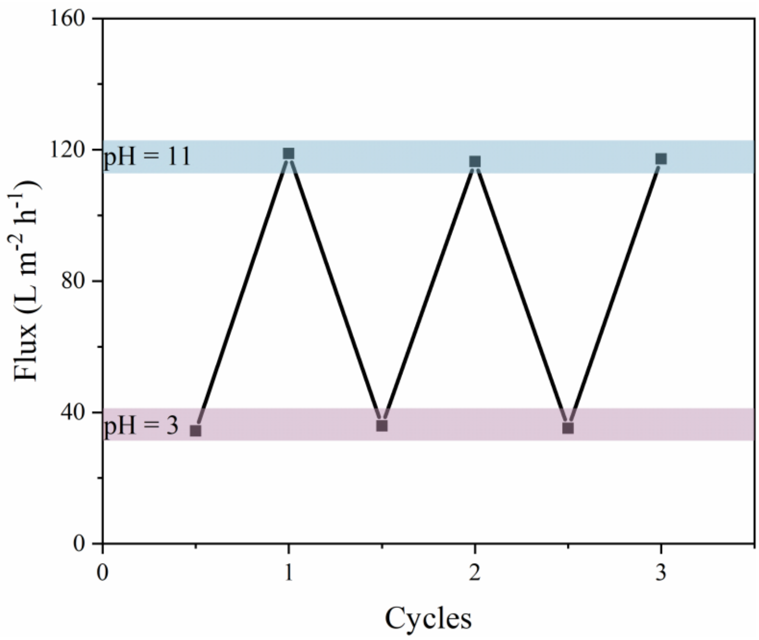

Figure R1-18. Flux of COF-CDN-12 membrane when the pH shifted from 3 to 11 after 3 cycles.

Page 18, Lines 403-405

"Furthermore, after three cycles of pH value shifting from 3 to 11, the COF-CDN-12 membrane maintained consistent water flux, with no significant decrease in performance observed at either pH value of 3 or 11, as shown in Supplementary **Fig. 28**a." **was added**.

Page S37

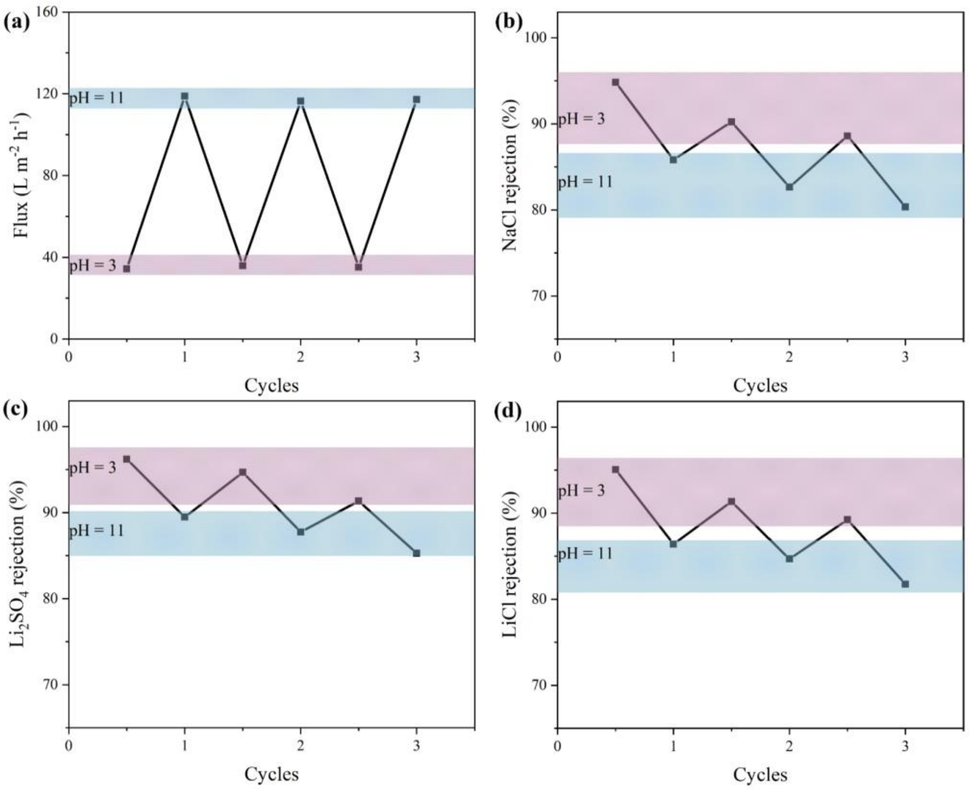

"

Supplementary Figure 28. (a) Flux, (b) NaCl, (c) Li_2SO_4 and (d) LiCl rejection of COF-CDN-12 membrane when the pH shifted from 3 to 11 after 3 cycles.

Note: As shown in Supplementary **Fig. 28**a, the COF-CDN-12 membrane maintained consistent water flux, with no significant decrease in performance observed at either pH value of 3 or 11." **was added**.

Comment R1-19: Could you authors comment on the scalability of COF-CDN membrane fabrication process?

Response R1-19: Thank the reviewer for the valuable guidance. In this study, the amount of COF we used is in the milligram range, which is significantly lower compared to traditional polymer membranes. Moreover, the amino monomer we use is already quite affordable, and with the advance of R&D of COF materials, the price of COF monomers is expected to decrease further, thereby reducing the cost of COF membranes. We employ a two-phase synthesis method that affords high-quality COF nanosheets as highly concentrated dispersions in excellent yield, and the procedure can be readily scaled up by simply increasing the reaction volume proportionally.

Additionally, we can optimize the preparation techniques, such as increasing the lateral size of COF nanosheets or incorporating additives to reduce COF consumption, ultimately lowering the membrane cost and facilitating industrial-scale production (*J. Am. Chem. Soc., 147(10), 8063-8082*). The deposition of COF nanosheets onto a substrate via vacuum filtration is compatible with continuous production processes. This simple and low-cost method allows for uniform membrane fabrication over a large area. Optimizing the preparation techniques greatly contributes to reducing the overall cost of COF membranes, thereby enhancing their commercial potential. Overall, the entire COF-CDN membrane fabrication process involves solution-based and vacuum-assisted steps that are inherently scalable. With appropriate engineering optimization, the process holds great potential for practical application.

Comment R1-20: The overall quality of the manuscript's writing needs improvement to ensure clarity and readability.

Response R1-20: Thank the reviewer for the valuable guidance. We have conducted a thorough, correcting grammatical errors, removing redundant phrasing, and improving sentence flow. All changes have been highlighted in yellow in the revised manuscript and the supplementary information to facilitate the reviewer's inspection.

Reviewer #2: The manuscript by Wei et al. presents a well-executed study on the development of a covalent organic framework (COF) membrane with hourglass-shaped hetero-nanochannels for high-performance desalination. By incorporating amino-cyclodextrin nanoparticles (CDN) onto COF membranes through sequential assembly, the authors successfully engineer nanochannels featuring a hydrophilic conical entrance and a hydrophobic spout, which enhance water transport while maintaining effective ion rejection. The introduction of pH-responsive functionality further enables dynamic regulation of channel size and charge, optimizing membrane performance. Moreover, the membrane demonstrates excellent stability over a seven-day operational period. The authors should be commended for their thorough characterization of the membrane. However, several minor revisions are required to improve the manuscript:

Response: We would like to thank the reviewer for the highly positive remarks and valuable guidance. We have thoroughly revised the manuscript and the supplementary information to address the concerns raised, making the necessary improvements and additions.

Comment R2-1: Figure Quality: Several figures, including Figures 1, 2, and 5, are of low resolution and appear blurry when zoomed in for details. Additionally, Figure 3(a) and Supplementary Information (SI) Figure 7 are overly light, making it difficult to discern membrane morphology. Higher-quality images should be provided to enhance clarity.

Response R2-1: Thank the reviewer for the valuable guidance. We have provided higher-quality versions of **Fig. 1**, **Fig. 2**, and **Fig. 5** to enhance clarity, and we have adjusted the brightness and contrast of **Fig. 3**a and Supplementary **Fig. 7** in the revised manuscript and the supplementary information.

Page 5, Line 81, Fig. 1 has been revised to:

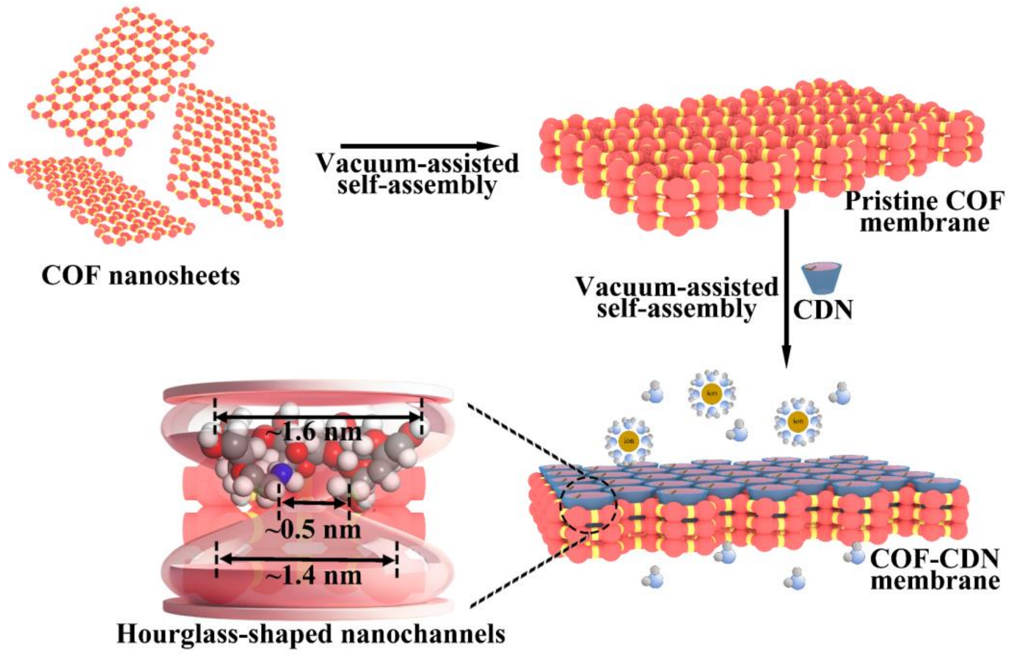

Page 7, Line 130, Fig. 2 has been revised to:

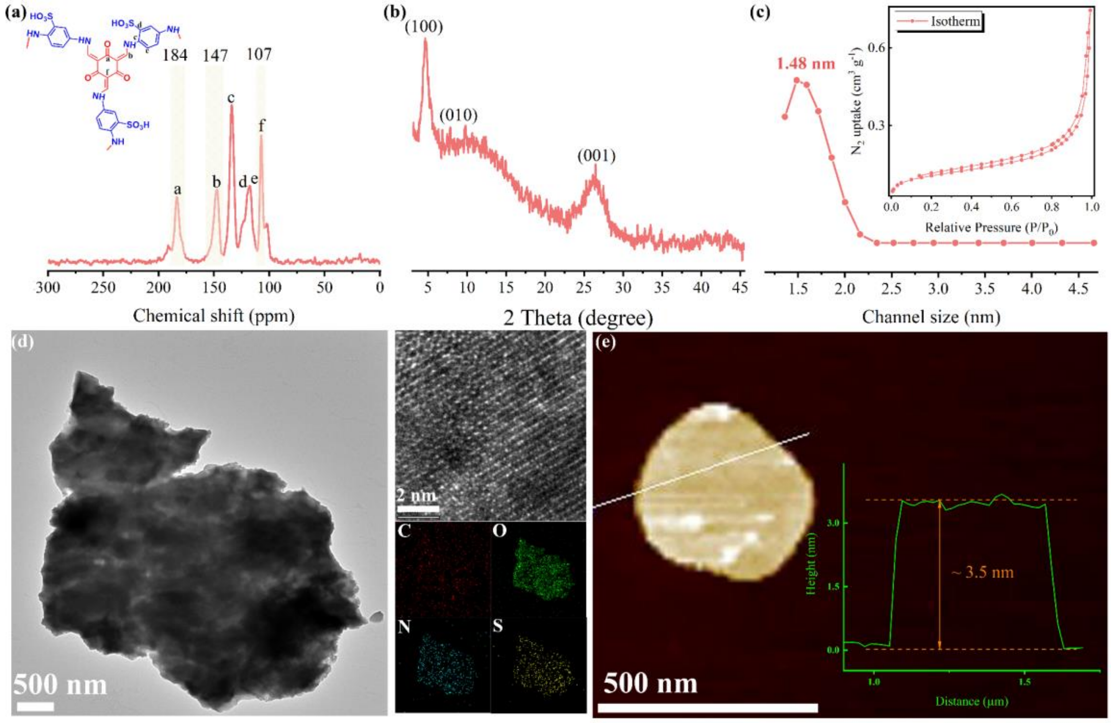

Page 11, Line 217, Fig. 3 has been revised to:

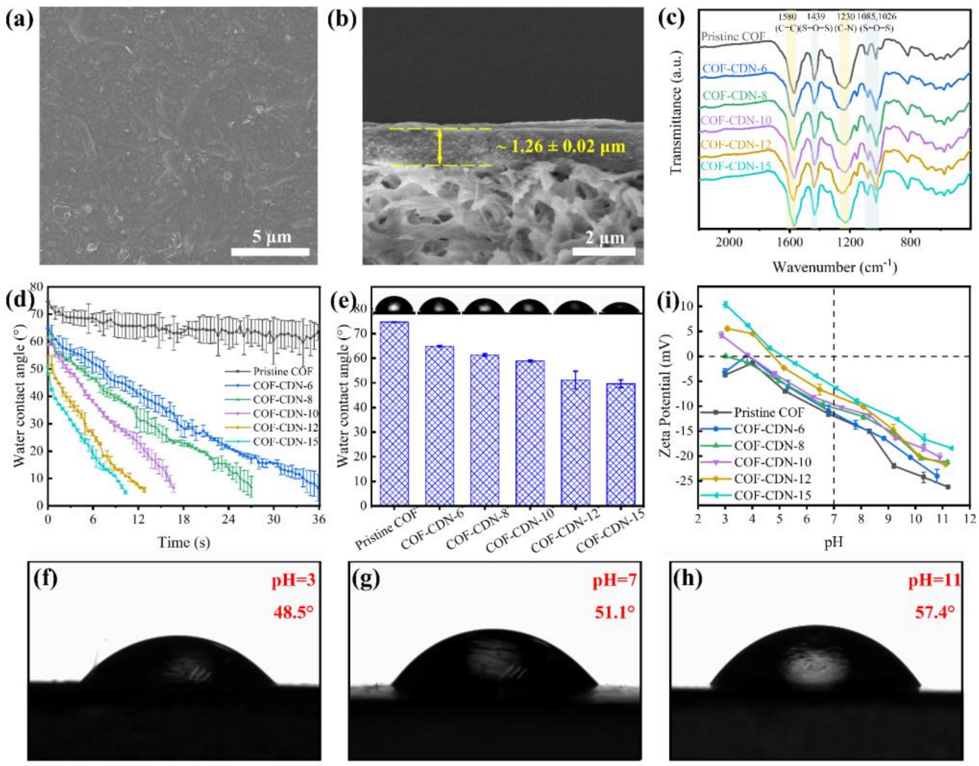

Page 18, Line 382, Fig. 5 has been revised to:

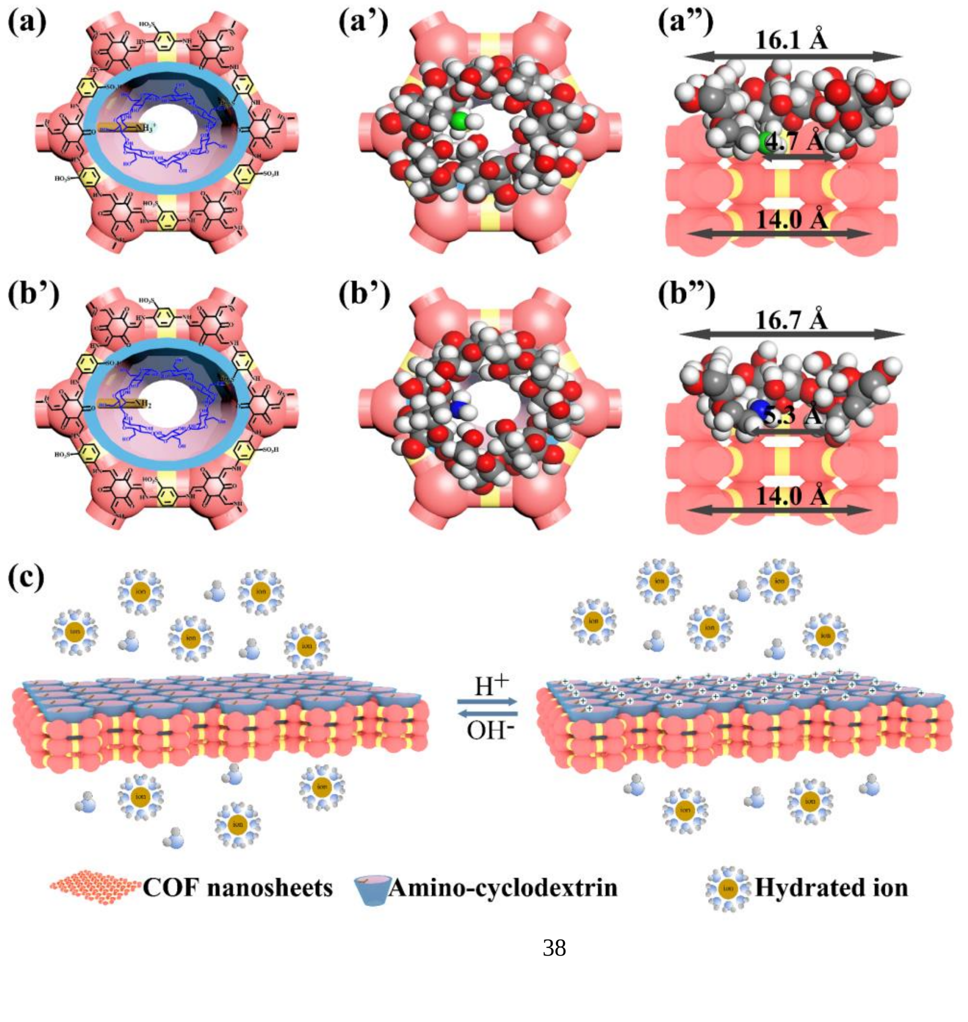

Page S12, Supplementary Figure 7 has been revised to:

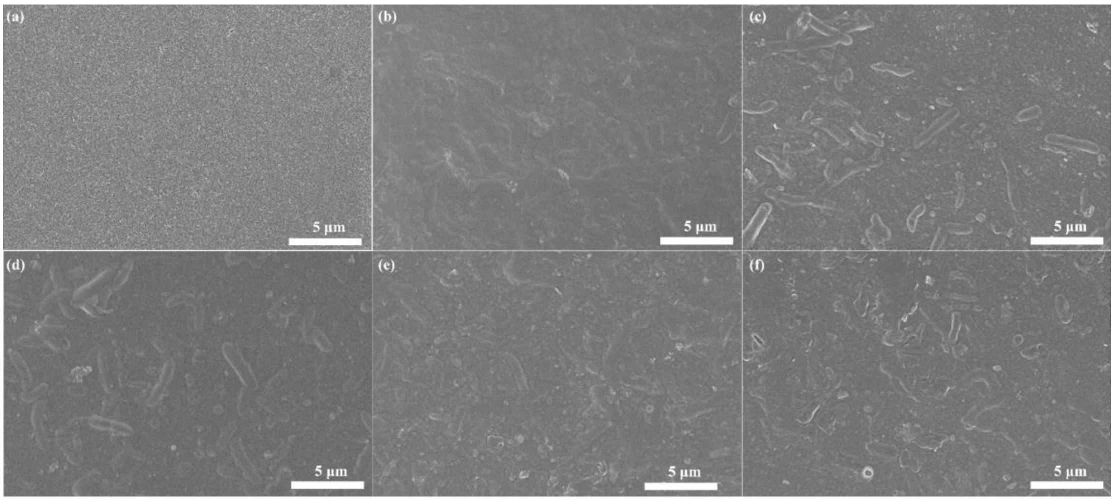

Comment R2-2: Technical Clarification (Lines 296–299): The claim that the positively charged membrane repels anions requires further clarification, as this is not consistent with expected electrostatic interactions. More insight is needed to explain why the membrane exhibits a higher rejection rate in acidic environments.

Response R2-2: Thank the reviewer for the valuable guidance. The observed electrostatic behavior is particularly relevant for multivalent anions such as SO_4^{2-}, which are more strongly influenced by electrostatic interactions than monovalent anions like Cl^-. While the positively charged membrane surface may initially attract divalent anions, this attraction can result in the formation of a condensed counterion layer near the membrane interface. This locally concentrated layer of anions enhances electrostatic screening and leads to secondary repulsive interactions that inhibit further anion transport from the solution. This mechanism helps explain the consistently higher rejection of Na_2SO_4 compared to NaCl under acidic conditions. Under neutral or alkaline conditions, where the amino groups are deprotonated and the membrane carries less positive surface charge, such an effect is significantly diminished, resulting in reduced electrostatic modulation and correspondingly lower rejection performance for multivalent salts. To provide a more comprehensive understanding, we performed DFT calculations that simultaneously consider cation and anion interactions in both protonated and deprotonated states, as shown in **Figure R1-9**, to support the when interpretation of ion rejection. The calculated overall binding energy ($Li_2SO_4 > Na_2SO_4 >$

$MgSO_4$ > LiCl > NaCl > $MgCl_2$) reflects the combined contributions of both anion and cation affinities toward the membrane, indicating that ion rejection is governed not only by the Donnan effect, but also by the CDN–ion interactions within the membrane.

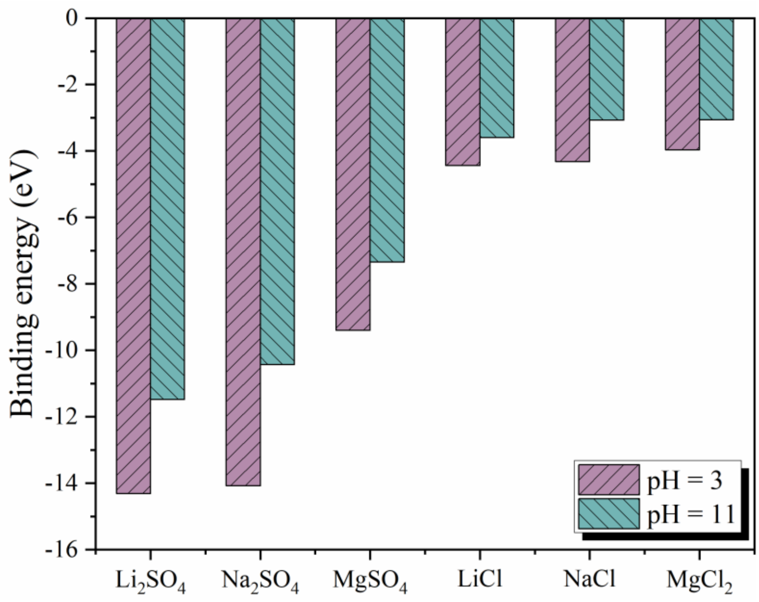

Figure R2-2 Binding energy between salt molecule and CDN at different pH values calculated by DFT

Page 15, Lines 311-318

"This effect was especially pronounced for multivalent anions, such as SO_4^{2-}, which experienced greater electrostatic repulsion than monovalent anions, such as Cl^-, resulting in consistently higher rejection for Na_2SO_4 compared to NaCl." **was revised to** "This electrostatic phenomenon was particularly significant for multivalent anions such as SO_4^{2-}, which experienced stronger repulsive forces compared to monovalent anions like Cl^-. The initial attraction of divalent anions to the positively charged membrane surface was proposed to result in the formation of a concentrated counterion layer, which subsequently amplified the repulsion of additional incoming anions from the solution. This mechanism was considered to contribute to the consistently higher rejection of Na_2SO_4 relative to NaCl under acidic conditions.".

Page 16, Lines 347-350

"Meanwhile, DFT calculations considering both cation and anion interactions under

protonated and deprotonated states indicated that ion rejection was controlled by interactions involving both cations and anions, rather than only by Donnan exclusion (Supplementary **Fig. 23**)." **was added**.

Page S5

"**Density functional theory calculation**

Quantum chemical calculations were performed using the Gaussian 09W software package[1] based on density functional theory (DFT). All molecular geometries were fully optimized at the hybrid B3LYP functional level employing the 6-31G (d, p) basis set, without imposing symmetry constraints. The optimized geometries were confirmed as global minima by frequency calculations, which indicated the absence of imaginary frequencies. Additionally, dispersion interactions were accounted for using Grimme's D3 correction method (B3LYP+D3). The basis set superposition error (BSSE) was evaluated and corrected to accurately estimate intermolecular interaction energies." **was added**.

Page S30

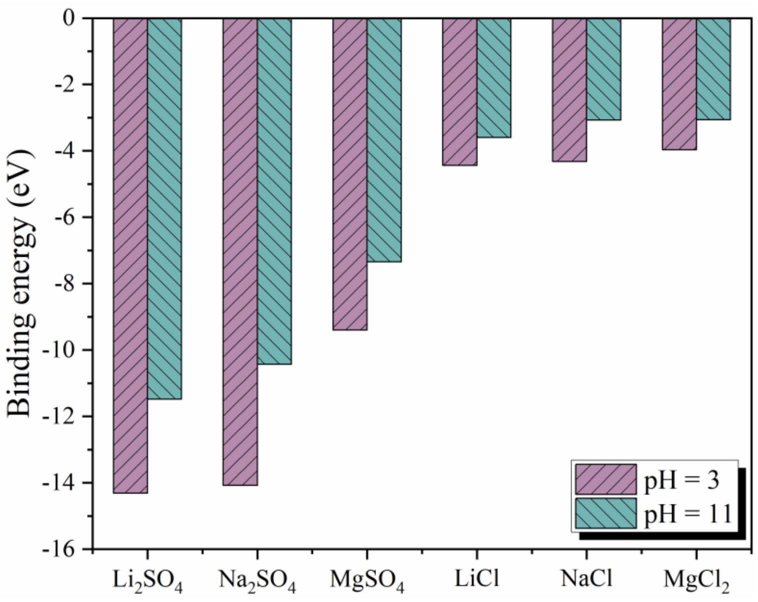

"

Supplementary Figure 23. Binding energy between salt and CDN at different pH values calculated by DFT.

Note: Meanwhile, DFT calculations were conducted considering both cation and anion

interactions under protonated and deprotonated states. The overall binding energy sequence (Li_2SO_4 > Na_2SO_4 > $MgSO_4$ > LiCl > NaCl > $MgCl_2$) was calculated, reflecting the combined contributions of anion and cation affinities toward the membrane. These results indicated that ion rejection was governed not only by the Donnan effect but also by the CDN–ion interactions within the membrane. This behavior was attributed to the fact that, after protonation ($-NH_2$ to $-NH_3^+$), the membrane surface became positively charged, which not only repelled cations but also attracted anions, particularly divalent anions such as SO_4^{2-}. The electrostatic attraction of anions could lead to the formation of a negatively charged anion concentration layer at the membrane surface, which might subsequently enhance the rejection of additional anions from the solution." **was added**.

Page S33

"At lower pH values, the amino groups on CDN were protonated, which imparted a positive charge to the membrane surface. The positively charged membrane surface influenced ion distribution within the nanochannels, reducing the passage of ions. In addition, the protonation caused the CDN cavity to shrink, with the hydrophilic conical entrance size of 16.1 Å and the hydrophobic spot size of 4.7 Å, causing the pores of COF-CDN membranes to either decrease in effective size or enter a "closed" state." **was revised to** "After protonation ($-NH_2$ to $-NH_3^+$), the membrane surface became positively charged, which not only rejected cations but also attracted anions. This attraction could lead to the formation of a negatively charged anion concentration layer at the membrane surface, which could subsequently enhance the rejection of additional anions from the solution. In addition, under acidic conditions, the amino groups in CDN were protonated, causing the CDN cavity to shrink, with the hydrophilic conical entrance size of 16.1 Å and the hydrophobic spot size of 4.7 Å. Consequently, the pore of COF-CDN membranes either decreased in effective size or enter a "closed" state.".

Comment R2-3: Data Interpretation and Characterization (Lines 350–354, Figure 6(b), and SI Figure 22): The manuscript states that rejection shows "no significant decrease,"

whereas the data clearly indicate a decline. A 1% difference is substantial in the context of desalination membranes, where even small variations can impact performance. This discrepancy should be corrected, and further explanation is required regarding the observed decline in rejection rate during pH shift cycles. Additional membrane characterization would be beneficial in elucidating this behavior.

Response R2-3: Thank the reviewer for the valuable guidance. We are sorry for the mistake, and we have made the relevant sections in the revised manuscript and the supplementary information to enhance the accuracy of our description.

As shown in **Fig. 6**b and Supplementary **Fig.28**b-d, a slight decline in rejection is observed at pH values of 3 or pH 11 during the cycling test, in which the pH value is shifted between 3 to 11. This can be attributed to partial retention of salt ions within the membrane pores (*Chem. Soc. Rev., 2022, 51, 2224-2254*, *Nat. Commun., 2022, 13, 7339*). Specifically, when the pH shifts from 3 to 11, the pores of COF-CDN membranes enter an "open" state, allowing salt ions to pass through more easily. Some of these salt ions become retained within the membrane pores. When the pH returns from 11 to 3, these residual salt ions interfere with the complete re-protonation of COF-CDN membranes, resulting in a slight decline in rejection during the subsequent cycle.

While we agree that additional membrane characterization can offer insights into transport behavior, we believe that a more direct way to verify the origin of the observed performance during pH cycling is to decouple the effect of residual ions. Therefore, instead of conducting additional membrane characterizations, we performed a control experiment by conducting pH cycling in water without added salt ions.

To further verify that the slight performance attenuation was caused by residual salt ions, we conducted pH cycling experiments using water, as shown in **Figure R2-3**. In the absence of salt ions, the water flux remains nearly unchanged throughout the cycles, confirming that the observed decline in rejection is primarily due to ion accumulation on the membrane surface. The combination of water flux stability and the limited rejection decrease demonstrate the reversible and reliable pH-responsive property of the COF-CDN-12 membrane, confirming its robust structural integrity, which is crucial for maintaining stable separation under fluctuating pH values.

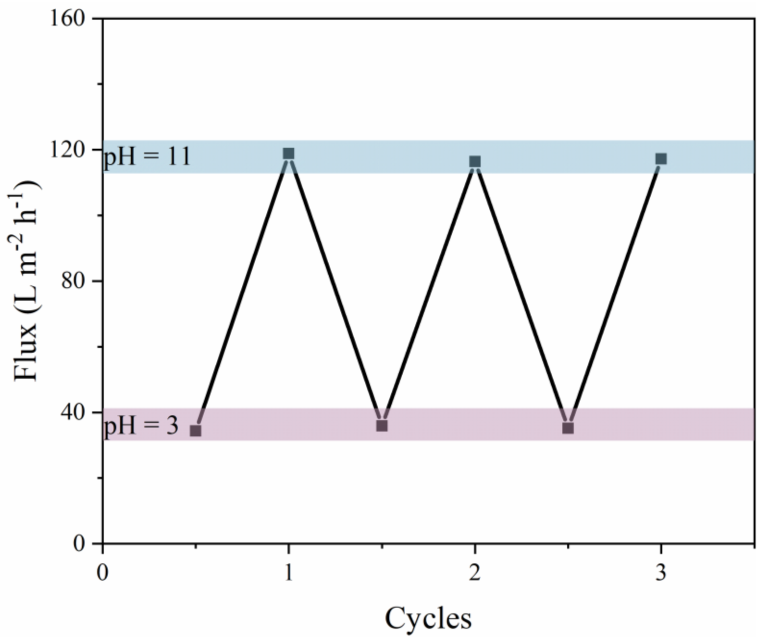

Figure R2-3. Flux of COF-CDN-12 membrane when the pH shifted from 3 to 11 after 3 cycles.

Page 19, Lines 402-405

"Furthermore, after three cycles of pH value shifting from 3 to 11, the COF-CDN-12 membrane maintained consistent water flux, with no significant decrease in performance observed at either pH value of 3 or 11, as shown in Supplementary **Fig. 23**a." **was added**.

Page 19, Lines 405-407

"Furthermore, after three cycles of pH value shifting from 3 to 11, the COF-CDN-12 membrane maintained consistent rejection for Li_2SO_4, Na_2SO_4, LiCl, NaCl, with no significant decrease in performance observed at either pH value of 3 or 11, as shown in **Fig. 6**b and Supplementary **Fig. 22**." **was revised to** "In contrast, as shown in **Fig. 6**b and Supplementary **Fig. 28**b-d, rejection for Li_2SO_4, Na_2SO_4, LiCl, NaCl exhibited a slight decline at either pH value of 3 or 11, due to partial retention of salt ions within the membrane pores[47,48].".

Page 19, Lines 407-411

"This result demonstrated the reversible and reliable pH-responsive property of the COF-CDN-12 membrane, confirming its robust structural integrity, which was crucial for maintaining stable separation under fluctuating pH values." **was revised to** "The combination of water flux stability and the limited rejection decrease demonstrated the reversible and reliable pH-responsive property of the COF-CDN-12 membrane, confirming its robust structural integrity, which was crucial for maintaining stable separation under fluctuating pH values.".

Page S37

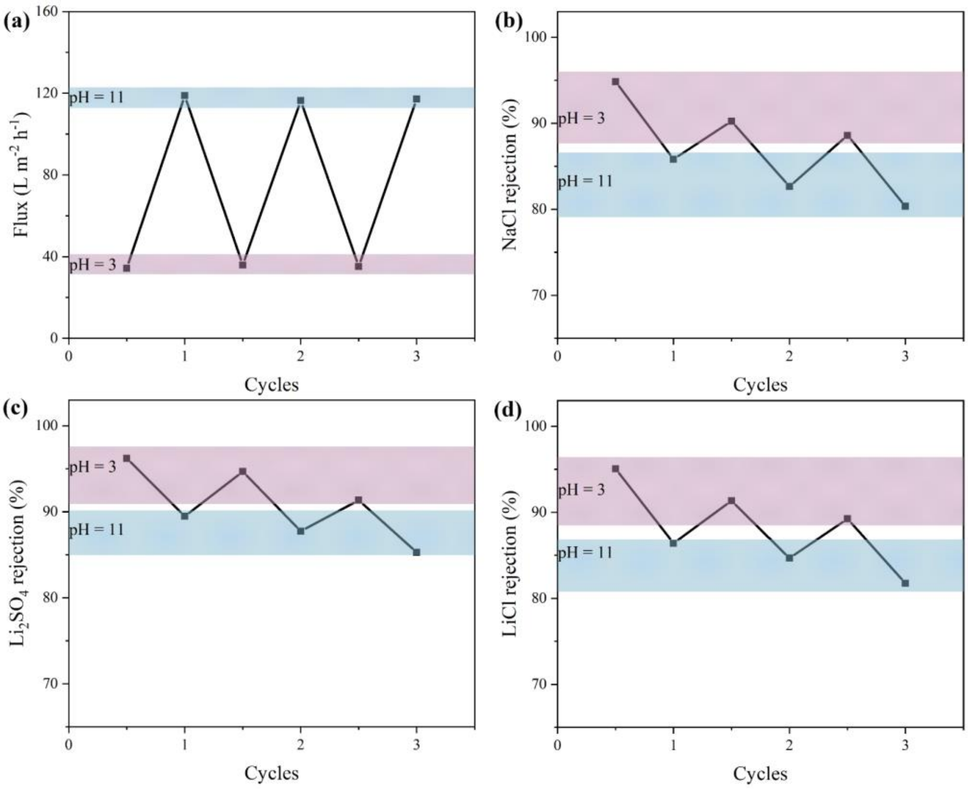

"

Supplementary Figure 28. (a) Flux, (b) NaCl, (c) Li_2SO_4 and (d) LiCl rejection of COF-CDN-12 membrane when the pH shifted from 3 to 11 after 3 cycles.

Note: As shown in Supplementary **Fig. 28**a, the COF-CDN-12 membrane maintained consistent water flux, with no significant decrease in performance observed at either pH value of 3 or 11. As shown in **Fig. 6**b and Supplementary **Fig. 28**b-d, a slight decline in rejection was observed at pH 3 or pH 11 during the cycling test, in which the pH value was shifted between 3 to 11. This was attributed to partial retention of salt ions

within the membrane pores. Specifically, when the pH shifted from 3 to 11, the pores of COF-CDN membranes entered an “open” state, allowing salt ions to pass through more easily. Some of these salt ions were retained within the membrane pores. When the pH returned from 11 to 3, these residual salt ions interfered with the complete re-protonation of COF-CDN membranes, resulting in a slight decline in rejection during the subsequent cycle." **was added**.

Reviewer #3: I co-reviewed this manuscript with one of the reviewers who provided the listed reports. This is part of the Nature Communications initiative to facilitate training in peer review and to provide appropriate recognition for Early Career Researchers who co-review manuscripts.

Response: We would like to thank the reviewer for the highly positive remarks and valuable guidance. We appreciate your co-review efforts, and we have addressed all comments and concerns, including those from your co-reviewer, in the revised manuscript and the supplementary information.

Reviewer #4: In this work, the authors designed and constructed hetero-nanochannels by incorporating amino-cyclodextrin nanoparticles (CDN) into the mouth of COF membrane to pursue ultrafast desalination. The resulting nanochannels consist of a wide hydrophilic conical entrance and a narrow hydrophobic spout onto cylindrical nanochannels. The COF membrane with such hetero-nanochannels achieves high desalination performance and significantly breaks the trade-off between membrane permeability and selectivity. This work is quite innovative and interesting, stimulating the thinking about the design of advanced membrane structures. This manuscript is well organized, and well-written. Some minor issues should be addressed before the manuscript is accepted.

Response: We would like to thank the reviewer for these highly positive remarks and valuable guidance. We have thoroughly revised the manuscript and the supplementary information to address the concerns raised, making the necessary improvements and additions.

Comment R4-1: The membrane name (COF-CD 4, COF-CD 7...) in Fig. 3c is different from that (COF-CDN-4, COF-CDN-7...) used elsewhere in this manuscript, which should be unified and corrected.

Response R4-1: Thank the reviewer for the valuable guidance. We are sorry for the mistake, and **Fig. 3c** has been carefully checked and corrected in the revised manuscript.

Page 11, Line 217, Fig. 3c has been revised to:

(c)

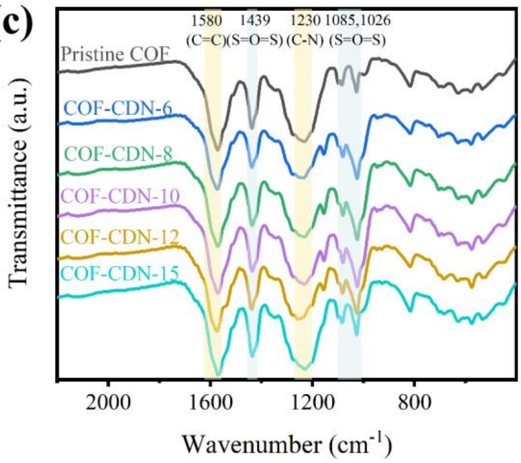

Comment R4-2: The quality of Fig. 4 needs to be improved, as the color and pattern appear pixelated or blurry in the provided image.

Response R4-2: Thank the reviewer for the valuable guidance. The quality of **Fig. 4** has been improved to enhance clarity in the revised manuscript.

Page 13, Line 275, Fig. 4 has been revised to:

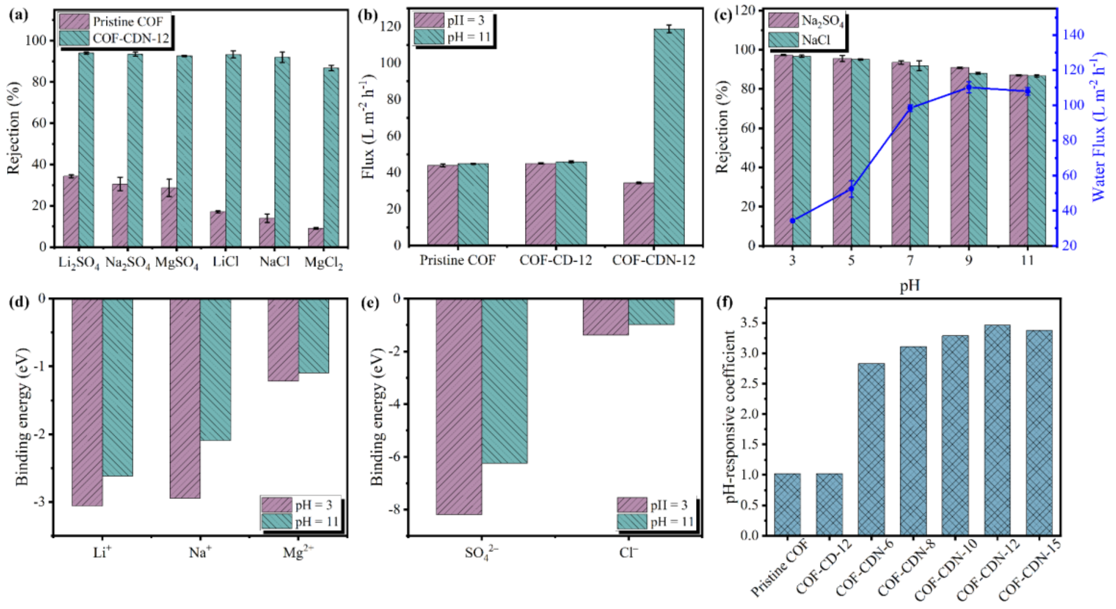

Comment R4-3: The experimental details for the activation (degassing) of the COF nanosheets in the nitrogen adsorption-desorption experiment should be added.

Response R4-3: Thank the reviewer for the valuable guidance. The samples were activated by degassing at 120°C for 8 h, and the pore size distributions were derived from the sorption data using non-local density functional theory (NLDFT). We have added experimental details for the COF nanosheets in the nitrogen adsorption-desorption experiment. The analysis details are provided in the revised supplementary as follows:

Page S4

"The samples were activated by degassing at 120°C for 8 h, and the pore size distributions were derived from the sorption data using non-local density functional theory (NLDFT)." **was added**.

Comment R4-4: A slight deviation is observed between the simulated and experimental XRD patterns of the COF powder, particularly in the (100) peak position. The explanations should be provided.

Response R4-4: Thank the reviewer for the valuable guidance. Indeed, there is a slight deviation between the simulated and experimental XRD patterns of the COF powder. This deviation is consistent with previous literature (*Chem. Mater., 2016, 28, 1489-1494*). Ideally, AA stacking in COF powder would produce ultrashort, vertically aligned transport channels through interconnected pores, resulting in significantly enhanced permeance. However, in our work and reported literature, the presence of AB stacking results in slight deviations from the simulated XRD patterns. In the revised supplementary information, we have added a discussion on this matter, which could be found below.

Page S7

"The slight deviation from the ideal crystal structure was consistent with previous literature[10]." **was added**.

Comment R4-5: As a comparison, the rejection data of COF-CD-12 should be offered, and explanations about the effect of the amino group on the rejection should be provided.

Response R4-5: Thank the reviewer for the valuable guidance. We have added the rejection data of the COF-CD-12 membrane and provided additional discussion regarding the effect of the amino group on the rejection in its pH-responsive property. The analysis details have been provided in the revised supplementary information as follows:

Page 14, Lines 295-298

"Similarly, the COF-CD-12 membrane by installing CD without amino groups onto the mouth of COF membrane did not exhibit pH-responsive property in water flux and rejection for Na_2SO_4 (**Fig. 4**b)." **was revised to** "Similarly, the COF-CD-12 membrane by installing CD without amino groups onto the mouth of COF membrane did not

exhibit pH-responsive property in water flux and rejection for Na_2SO_4 and NaCl (**Fig. 4**b and Supplementary **Fig. 22**).

Page S29

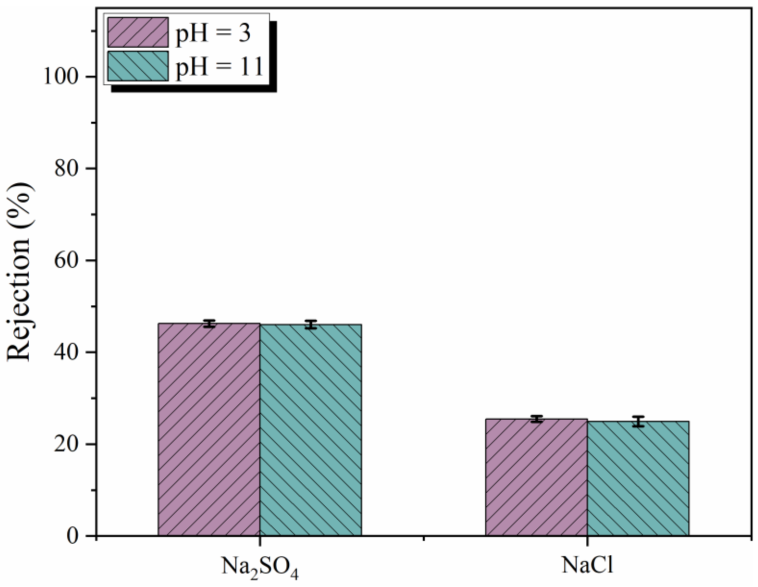

"

Supplementary Figure 22. Rejection of COF-CD-12 membrane at pH values of 3 and 11.

Note: For the COF-CD-12 membrane by installing CD without amino groups onto the mouth of COF membrane, the rejection of 46% for Na_2SO_4 and 25% for NaCl remained unchanged at pH values of 3 and 11, indicating that the COF-CD-12 membrane lacked pH-responsive property. These results demonstrated that the amino group plays a key role in the pH-responsive property by dynamically regulating of the effective size and charge." **was added**.

Response to comments

Reviewer #1: The authors have addressed all concerns raised by the reviewers satisfactorily. I recommend that the paper be accepted for publication.

Response: We would like to thank the reviewer for the highly positive remarks and the strong support of the publication of this work.

Reviewer #2: In this manuscript, the authors the synthesize and characterize a COF-based membranes and test it for water flux and salt rejection. There are a few issues with this manuscript:

Response: We would like to thank the reviewer for the highly positive remarks and valuable guidance.

Comment R2-1: The salt rejection values are not great, and correspond to a NF-type membrane. It is not clear what such a membrane is useful for. It is too low for seawater (and was not evaluated under seawater conditions), and low salt rejecting membranes are abundantly available from many commercial vendors. Not clear where such a membrane fits into the existing landscape, nor what is the point of using it (assuming it can be manufactured at scale, which is not explored here).

Response R2-1: Thank the reviewer for the valuable guidance. The COF-CDN membrane developed in this work features hetero-nanochannel structures with pH-responsive property, enabling dynamic regulation of effective pore size and surface charge and allowing tunable flux and selective separation under varying pH conditions. To further assess its potential under more realistic conditions, we conduct a two-stage filtration test using a mixed salt solution containing NaCl and $MgCl_2$ (at a concentration of 1000 ppm). After two successive filtrations, the salt concentration in the final permeate decreases from 1000 ppm to approximately 3 ppm with total salt rejection of 99.7%, meanwhile, our COF-CDN membrane possesses a water flux of 98 L m^{-2} h^{-1} measured at 2.0 bar. This result demonstrates that the COF-CDN membrane can achieve deep desalination with high water flux when operated in a staged configuration, indicating its promise for practical application. The hetero-nanochannel design not only

facilitates adaptive mass transport control, but also presents a promising strategy to break the trade-off between membrane permeability and selectivity in various separations. Furthermore, the membrane exhibits excellent antifouling properties (Supplementary **Fig. 18**), consistent rejection performance in mixed salt solutions (Supplementary **Fig. 19**), significant chlorine tolerance (Supplementary **Fig. 20-21**), and stability (**Fig. 6**), supporting its potential utility in advanced filtration processes. In the revised manuscript, we have clarified the target application scenario, stating that the membrane targets pH-responsive applications requiring dynamic control, such as smart filtration of molecules and ions.

In addition, we acknowledge that all current experiments are carried out at lab-scale using synthetic salt solutions. While these results provide strong proof-of-concept for the membrane structure–function relationship and potential utility, further studies involving more complex feed compositions and pilot-scale systems remain necessary to further validate in real-world application. Moreover, scalability is an important consideration. While a detailed scale-up analysis is beyond the current scope, we would like to point out that the membrane is fabricated under mild conditions without specialized equipment. This method is inherently scalable and therefore holds good potential for future development. In the revised manuscript, we have added relevant discussion about this, which could be found below.

Page 13, Lines 274-281

"Building upon this, a two-stage filtration test was performed to further evaluate its potential under more realistic conditions. After two successive filtrations, the salt concentration in the final permeate was reduced from 1000 ppm to approximately 3 ppm with total salt rejection of 99.7%, meanwhile, our COF-CDN membrane possesses a water flux of 98 L m^{-2} h^{-1} measured at 2.0 bar. This result demonstrates that the COF-CDN membrane achieves deep desalination with high water flux when operated in a staged configuration, indicating its promise for practical application." **was added**.

Page 19, Lines 437-439

"These findings indicate the applicability of the COF-CDN membrane in separation processes requiring dynamic control, such as smart filtration." **was added**.

Page 21, Lines 482-484

"The entire process proceeds under ambient temperature and modest vacuum pressure without specialized equipment, making it readily adaptable to larger substrates." **was added**.

Comment R2-2: The flux data is unclear. At one point, the authors state that they normalize the flux by thickness. However, in other figures (e.g., Figure 4) the flux is listed as not normalized, although the values are very similar to the normalized values. Which is it? Also, flux should be expressed as permeability (e.g., LMH/bar) in these kinds of studies.

Response R2-2: Thank the reviewer for the valuable guidance. Indeed, we apply thickness normalization of flux to eliminate the effect of thickness and compare membrane performance under equivalent thickness conditions. However, we emphasize that this normalization is used only as an analytical tool, specifically for interpreting the role of hourglass-shaped nanochannels and hydrophilicity in overcoming thickness-induced resistance (Supplementary **Fig. 26**). In all performance evaluations throughout the manuscript, including **Fig. 4**, we consistently use non-normalized flux values ($L\ h^{-1}\ m^{-2}$) under a constant operating pressure of 2.0 bar, which is the standard metric for practical membrane comparison. To avoid any misunderstanding, we have clarified this in the revised manuscript and the supplementary information, and have added explicit note of the operating pressure (2.0 bar) at each location where flux is discussed. This ensures clarity and consistency in data interpretation.

Page 22, Lines 501-506

"In this study, all water flux were reported as non-normalized flux ($L\ h^{-1}\ m^{-2}$) under a constant pressure of 2.0 bar, unless otherwise specified. Normalized flux was calculated

and used solely for performance interpretation in specific sections, such as **Fig. 6**a for the evaluation of stability, Supplementary **Fig. 18** for the evaluation of antifouling properties, and Supplementary **Fig. 28** for the elimination of membrane thickness variation effects." **was added**.

Comment R2-3: There is no mention of chlorine tolerance. This is an important point, and really the only reason such a material may be useful (assuming it has some tolerance). This was called out by a reviewer but was not addressed, suggesting there is an issue?

Response R2-3: We thank the reviewer for pointing this out again and fully understand the importance of chlorine tolerance for membranes. In the initial revision, we did not include additional chlorine tolerance experiments because a previous reviewer had positively acknowledged this potential feature, stating: "Nevertheless, apart from the enhanced desalination performance, another notable strength of this work is the potential chlorine resistance of the proposed membrane design, which could offer significant advantages over conventional polyamide-based membranes." Based on that recognition, we did not conduct further validation at that stage. This time, we have conducted additional experiments to directly evaluate the chlorine tolerance of the COF-CDN membrane. The membrane was soaked in 100 ppm sodium hypochlorite solution for 24 hours. After soaking, the membrane maintains both its structural integrity (as confirmed by FT-IR, Supplementary **Fig. 20**) and desalination performance (water flux and salt rejection are consistent with those before soaking, Supplementary **Fig. 21**). This is attributed to the chemically stable framework of COF nanosheets, which does not contain oxidation-prone amide groups typically found in polyamide membranes that are susceptible to chlorine attack (*Nat. Commun., 2023, 14, 5483*, *Nat. Rev. Methods Primers, 2024, 4, 10*). The π-conjugated aromatic framework of COF nanosheets provides oxidative robustness, which remain stable under hypochlorite conditions (*Angew. Chem. Int. Ed., 2022, 61, e202202912*, *J. Am. Chem. Soc., 2022, 14450, 23158-23167*). The FT-IR spectra show no evidence of bond cleavage or functional group degradation, supporting the structural integrity of both the COF and

the CDN. These results confirm that the COF-CDN membrane exhibits excellent chlorine tolerance.

Page 13, Lines 281-292

"After soaking in 100 ppm sodium hypochlorite solution for 24 hours, the membrane preserves its structural integrity (FT-IR, Supplementary **Fig. 20**) and maintains stable desalination performance (water flux and salt rejection are consistent with those before soaking, Supplementary **Fig. 21**). This was attributed to the chemically stable framework of COF nanosheets, which lacked oxidation-prone amide groups commonly found in polyamide membranes, that were susceptible to chlorine attack[41,42]. The π-conjugated aromatic framework of the COF nanosheets provided oxidative robustness, which remained stable under hypochlorite conditions[30,43]. The FT-IR spectra showed no signs of bond cleavage or functional group degradation, supporting the structural integrity of both the COF and the CDN. These results confirmed that the COF-CDN membrane exhibited excellent chlorine tolerance." **was added**.

Page S28

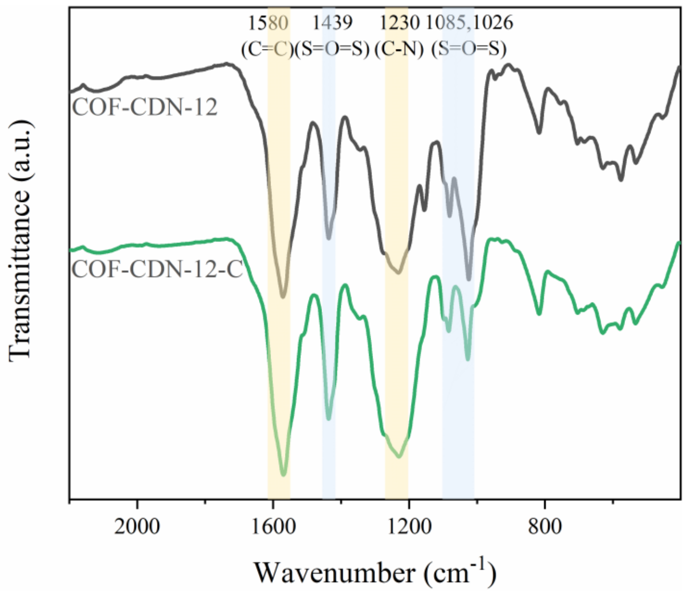

"

Supplementary Figure 20. FT-IR spectra of COF-CDN-12 membrane before (COF-CDN-12) and after (COF-CDN-12-C) treatment with 100 ppm sodium hypochlorite solution for 24 hours.

Note: After the treatment, FT-IR spectra indicated that the functional groups remained intact, thereby demonstrating the excellent chemical stability of membrane under oxidative conditions." **was added**.

Page S29

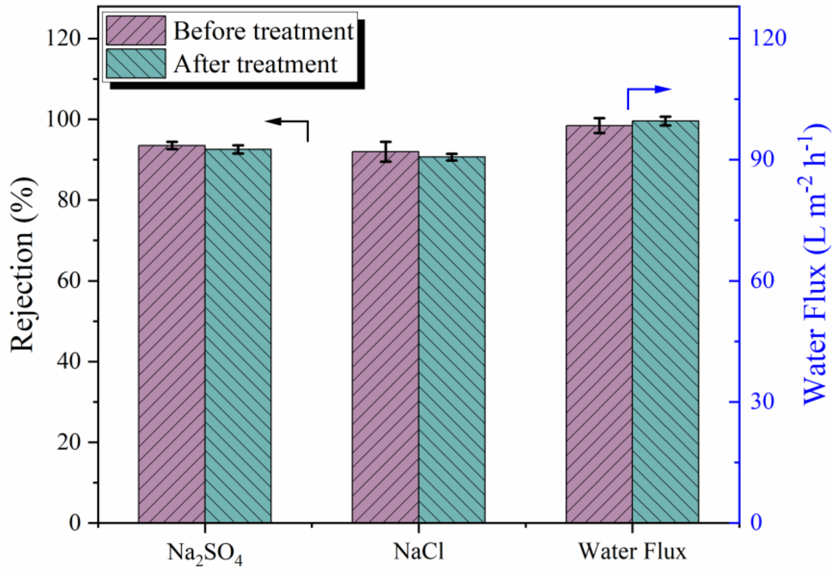

"

Supplementary Figure 21. Desalination performance of the COF-CDN-12 membrane before and after treatment with 100 ppm sodium hypochlorite solution for 24 hours.

Note: To further evaluate the chlorine tolerance, we conducted desalination tests after treatment with 100 ppm sodium hypochlorite solution for 24 hours. The water flux and salt rejection obtained after soaking were consistent with those measured before treatment, indicating that the membrane maintained its effective performance under chlorinated conditions." **was added**.

Comment R2-4: The data in Figure 6c is not clear. It is not reasonable to compare NaCl and Na_2SO_4 rejection-the latter will always have far higher rejection.

Response R2-4: Thank the reviewer for the valuable guidance. Our intention is not to directly compare the rejection of NaCl and Na_2SO_4 against each other. Instead, we aim to benchmark the performance of COF-CDN-12 membrane against representative state-of-the-art membranes using NaCl and Na_2SO_4 separately. Specifically, we compare the NaCl rejection of COF-CDN-12 membrane with the NaCl rejection of other membranes,

and similarly, compare the Na_2SO_4 rejection of COF-CDN-12 membrane with that of other membranes. To avoid misunderstanding, we revise **Fig. 6**c by clearly labeling the NaCl and Na_2SO_4 rejection of COF-CDN-12 membrane separately. The updated figure now more accurately reflects the cross-comparison with existing membrane performance for each individual salt, rather than implying a comparison between the two salts. We have also revised the relevant sections in the revised manuscript to enhance the accuracy of our description.

Page 20, Line 440, Fig. 6c has been revised to:

(c)

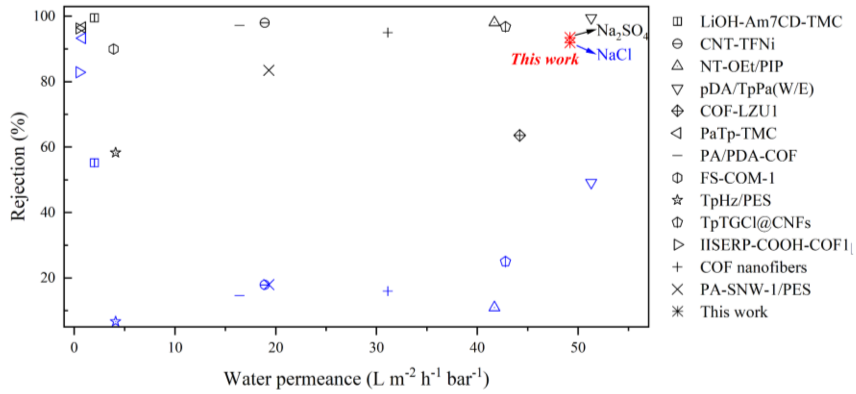

Page 20, Lines 444-447

"(c) the performance of COF-CDN-12 membrane, as compared to the state-of-the-art membranes (Supplementary **Table 2**). The black point indicates Na_2SO_4 rejection, and the blue point indicates NaCl rejection." **was revised to** "(c) the performance of COF-CDN-12 membrane, as compared to the state-of-the-art membranes (Supplementary **Table 2**). The red point represents this work, while the black and blue points indicate the rejection of Na_2SO_4 and NaCl, respectively.".

I guess I just don't "get" what the point of this paper is.

Response: We appreciate the reviewer's comment and would like to clarify the central contribution of our work. The key point of this study is to propose a design strategy for COF membranes incorporating hetero-nanochannels, constructed by installing amino-cyclodextrin (CDN) onto the mouth of COF membrane. This architecture affords

hourglass-shaped nanochannels, which allow the membrane to achieve both high water flux and salt rejection, addressing a long-standing trade-off in membrane technology. These hetero-nanochannels consist of the wide hydrophilic entrance for efficient water capture, the hydrophobic spout and the homo-nanochannels for fast water transport, and pH-responsive ability to dynamically regulate their effective size and charge for selective ion rejection. This hourglass-shaped nanochannels enables the membrane to adapt to changing separation requirements and offers performance advantages not easily achieved with homo-nanochannels COF or commercial nanofiltration membranes. Our study may therefore serve as an example for designing COF membranes with spatially heterogeneous nanochannels for efficient separations where both permeability and selectivity are required.

Reviewer #3: I co-reviewed this manuscript with one of the reviewers who provided the listed reports. This is part of the Nature Communications initiative to facilitate training in peer review and to provide appropriate recognition for Early Career Researchers who co-review manuscripts.

Response: We would like to thank the reviewer for the highly positive remarks and valuable guidance. We appreciate the co-review efforts and have addressed all comments, including those from your co-reviewer, in the revised manuscript and supplementary information.

Reviewer #4: The authors have adequately addressed my previous concerns. I believe the paper is now in a suitable form for publication.

Response: We would like to thank the reviewer for the highly positive remarks and the strong support of the publication of this work.

Response to comments

Reviewer #2: The authors have addressed my concerns.

Response: We would like to thank the reviewer for the highly positive remarks.

Reviewer #3: I co-reviewed this manuscript with one of the reviewers who provided the listed reports. This is part of the Nature Communications initiative to facilitate training in peer review and to provide appropriate recognition for Early Career Researchers who co-review manuscripts.

Response: We would like to thank the reviewer for the highly positive remarks. We appreciate the co-review efforts and have addressed all comments, including those from your co-reviewer, in the revised manuscript and supplementary information.
